# Dauricine Mitigates Hypoxia Through Targeting ESR1, PIK3CA, and MTOR: A Network Pharmacology and Molecular Dynamics Simulation Investigation

**DOI:** 10.3390/cimb48060550

**Published:** 2026-05-23

**Authors:** Zengxun Ni, Zineng Zhou, Feipeng Jia, Jingcheng Wu, Junhao Qiu, Kangrui Yuan, Zhicheng Jia

**Affiliations:** 1 College of Mechanical and Electronic Engineering, Nanjing Forestry University, Nanjing 210037, China; 2775660672@njfu.edu.cn (Z.N.);; 2 College of Food Science and Technology, Nanjing Agricultural University, Nanjing 210095, China; 3 The First School of Clinical Medicine, Nanjing Medical University, Nanjing 211166, China; 4 College of Chemical Engineering, Nanjing Forestry University, Nanjing 210037, China

**Keywords:** Dauricine, hypoxia, network pharmacology, molecular docking, molecular dynamics simulation

## Abstract

Hypoxia is a prevalent pathophysiological condition. Prolonged exposure to hypobaric hypoxia can lead to maladaptation, increasing the risk of chronic hypoxic diseases such as high-altitude polycythemia (HAPC). Dauricine, an alkaloid derived from the root of Menispermum dauricum DC, has been demonstrated to possess anti-hypoxic properties; however, its underlying molecular mechanisms remain elusive. In this study, a potential multi-target anti-hypoxic mechanism of dauricine was proposed and computationally evaluated using an integrated approach combining network pharmacology, molecular docking, and molecular dynamics simulations. Common targets between dauricine and hypoxia-related genes were identified through network pharmacology screening. A protein–protein interaction (PPI) network was constructed to identify core targets, followed by Gene Ontology (GO) functional enrichment and Kyoto Encyclopedia of Genes and Genomes (KEGG) pathway analyses. Molecular docking was subsequently employed to evaluate the binding affinities between dauricine and the candidate core targets, while molecular dynamics simulations were performed to assess the dynamic stability of the resulting complexes. Additionally, the drug-likeness and safety profiles of dauricine were assessed. The results suggest that dauricine may exert its anti-hypoxic effects by modulating candidate core targets, including ESR1, PIK3CA, and MTOR, and by acting on key signaling pathways such as PI3K-Akt, MAPK, and mTOR. This study provides a theoretical foundation for the further investigation of dauricine as a multi-target candidate for intervention in hypoxia and establishes a bioinformatics basis for subsequent experimental validation.

## 1. Introduction

Globally, over 80 million people reside permanently at altitudes exceeding 2500 m, with an additional 40 million traveling to these regions annually, thereby exposing themselves to chronic hypobaric hypoxia [1]. Clinical observations have demonstrated that prolonged exposure to such environments can induce neurological symptoms, including dizziness and ataxia. In severe cases, this exposure may even lead to arterial thrombosis in both the posterior and anterior cerebral circulations, subsequently resulting in ischemic brain injury [2]. Chronic mountain sickness represents a major health threat for populations long-term residing in high-altitude hypoxic environments, fundamentally arising from the body’s maladaptation and decompensation to chronic hypoxia [3]. Consequently, the development of novel strategies to effectively enhance hypoxic adaptation and mitigate hypoxic injury, thereby improving patients’ quality of life, holds profound significance for both clinical practice and global public health governance.

Traditional Chinese medicine (TCM), utilized as a form of complementary and alternative medicine in China for over two millennia, is also widely applied in the treatment of various oncological diseases [4]. Dauricine, a bisbenzylisoquinoline alkaloid extracted from the root of Menispermum dauricum DC (a plant belonging to the Menispermaceae family) [5], boasts a long history of medicinal use in China. It has been demonstrated to possess diverse pharmacological activities, including neuroprotection, anti-cancer effects, anti-arrhythmic properties, anti-inflammatory actions, anti-diabetic potential [6], and notable antihypertensive efficacy [7]. Recent investigations have revealed that dauricine mediates the accumulation of HIF-1α protein by modulating the activation state of the PI3K/AKT/mTOR signaling pathway [8], underscoring its significant potential in mitigating hypoxia.

Dauricine itself exhibits multi-target characteristics, rendering traditional single-target research paradigms insufficient to fully elucidate its comprehensive mechanism of action. Network pharmacology, an emerging strategy integrating systems biology, polypharmacology, and computational analysis [9], aligns well with the “multi-component, multi-target, multi-pathway” paradigm of TCM. It enables the systematic elucidation of complex interaction networks between drugs and diseases, rendering it particularly suitable for investigating bioactive components of TCM in the context of complex diseases [10]. Herein, by integrating network pharmacology, molecular docking, and molecular dynamics simulation techniques, we computationally explore a potential mechanistic framework through which dauricine enhances hypoxic adaptation. This study aims to establish a robust bioinformatics foundation for subsequent experimental validation and further pharmacological investigation.

## 2. Methods

### 2.1. Prediction of Active Compound Targets

The SMILES structure of dauricine was obtained from the PubChem database (https://pubchem.ncbi.nlm.nih.gov/, accessed on 29 January 2026) [11] and submitted to the SwissTargetPrediction platform (https://swisstargetprediction.ch/, accessed on 29 January 2026) for potential target prediction, with *Homo sapiens* selected as the target species. The top 100 predicted targets returned by the platform were retained as the initial candidate target pool, and no additional probability score cutoff was applied. It should be noted that the targets identified at this stage were used only as preliminary computational candidates for subsequent network pharmacology analysis and should not be interpreted as experimentally validated direct targets of dauricine.

### 2.2. Acquisition of Potential Hypoxia Adaptation-Related Targets

To systematically identify potential targets associated with hypoxia adaptation, hypoxia-related genes were retrieved from four public databases using “hypoxia” as the keyword, including PharmGKB (https://www.pharmgkb.org/, accessed on 29 January 2026), OMIM (https://omim.org, accessed on 29 January 2026), GeneCards (https://www.genecards.org, accessed on 29 January 2026), and DisGeNET (https://disgenet.com/, accessed on 29 January 2026). For the GeneCards database, genes with a relevance score ≥ 0.55 were retained. This threshold was used as an operational preliminary filtering criterion to exclude entries with very weak relevance while preserving sufficient coverage of hypoxia-related genes, considering that hypoxia is a complex pathophysiological condition involving multiple biological processes and signaling pathways. This cutoff was applied only during the initial GeneCards-based disease-gene filtering step and was not used as the sole criterion for final core target identification. The retrieved genes from different databases were then pooled and deduplicated to generate an integrated hypoxia-associated gene set. This gene set was subsequently used for intersection analysis with the predicted dauricine targets using the jvenn online tool (https://jvenn.toulouse.inrae.fr/app/example.html, accessed on 29 January 2026), and a Venn diagram was generated to visualize the overlap between drug-related targets and hypoxia-related genes.

### 2.3. PPI Network Construction and Screening of Core Topological Targets

To explore potential targets of dauricine in the treatment of hypoxia, a cross-analysis was performed between dauricine targets and hypoxia-related targets. The intersecting genes between active compound targets and hypoxia adaptation-related targets were identified using the jvenn tool. These intersecting genes were then imported into the STRING database (https://cn.string-db.org/, accessed on 30 January 2026) for protein–protein interaction (PPI) network analysis [12], with *Homo sapiens* selected as the species and a minimum interaction score threshold set at 0.4; all other parameters were set to default values. The PPI results were visualized using Cytoscape 3.8.0. The importance of each node was scored using six topological algorithms (MCC, MNC, Degree, EPC, Radiality, and Stress) from the CytoHubba plugin. The top 10 targets from each algorithm were visualized, and their intersection was defined as core topological targets for subsequent analysis.

### 2.4. GO and KEGG Enrichment Analyses

Gene Ontology (GO) enrichment analysis is a statistical method based on the hypergeometric distribution, while the Kyoto Encyclopedia of Genes and Genomes (KEGG) database integrates genomic, chemical, and systemic functional information. Enrichment analyses of GO terms and KEGG pathways are essential for elucidating the mechanisms of the traditional Chinese medicine component dauricine in relation to diseases.

In this study, GO functional enrichment analysis was performed on the intersecting genes using the DAVID database (https://david.ncifcrf.gov, accessed on 30 January 2026), with a significance threshold set at *p* value < 0.01. Through the analysis of biological processes (BPs), cellular components (CCs), and molecular functions (MFs), a total of 151 GO terms were significantly enriched under the aforementioned *p*-value threshold, including 37 MFs, 16 CCs, and 98 BPs. Concurrently, to elucidate relevant biological pathways, KEGG pathway enrichment analysis was conducted using the same database, with the species restricted to *Homo sapiens* [13]. Key enrichment results were visualized using the Microbioinformatics online platform (https://www.bioinformatics.com.cn, accessed on 31 January 2026) [14], and the associations between primary genes and functional terms were displayed using the CNSknowall platform (https://www.cnsknowall.com/, accessed on 31 January 2026).

### 2.5. GeneMANIA Functional Association Network Analysis of Core Targets

GeneMANIA (https://genemania.org, accessed on 31 January 2026) is a powerful tool for exploring functional associations among genes, enabling the prediction of additional genes related to the input set, many of which may serve as valuable therapeutic targets. To gain a more comprehensive understanding of the functional network of core genes, we expanded each core gene by adding the top 10 functionally related genes based on co-expression, physical interactions, and genetic associations. This approach constructed a more interconnected functional module, facilitating the accurate identification of potential therapeutic targets.

### 2.6. GO and KEGG Enrichment Analysis of GMFA Data

The genes obtained through GeneMANIA expansion were subjected to deduplication, yielding a unique set of 76 targets. To further elucidate the biological functions and pathways associated with this gene set, GO annotation and KEGG pathway enrichment analyses were performed using the DAVID database, and the resulting enrichment data were visualized using the Microbioinformatics online platform.

### 2.7. Construction of Compound-Target-Pathway-Disease Network and Screening of Core Compounds/Targets

The association data linking drug-components, component-targets, disease-targets, and pathway-targets, and pathways to targets were integrated and imported into Cytoscape 3.8.0 to construct a multi-dimensional biological network. Subsequently, topological parameter analysis was performed on the network using the CytoNCA module to evaluate the significance of each node within the network. Based on this assessment, core active components and core functional targets were identified.

### 2.8. Screening and iHypoxia Cross-Checking of Core Targets

The core targets identified through topological analysis and those derived from network-based functional screening were compared, and their intersection was defined as the final set of key targets involved in hypoxia adaptation. To further enhance the reliability of the predictions, these intersecting targets were further cross-checked using the iHypoxia database (https://ihypoxia.omicsbio.info/, accessed on 31 January 2026) [15]. This database systematically integrates data on protein expression dynamics under hypoxic conditions across multiple mammalian species, encompassing approximately 400,000 quantification events and 40,000 hypoxia-regulated proteins, thereby providing robust evidence for the hypoxia relevance of the identified targets.

### 2.9. Molecular Docking and ADME Evaluation

Molecular docking is a computational simulation method used to predict the binding modes and affinities between small molecules (drugs) and protein receptors (targets). In this study, this technique was employed to evaluate the potential binding affinity between dauricine and the core targets [16]. The three-dimensional structures of the target proteins were obtained from the RCSB PDB database (https://www.rcsb.org/, accessed on 2 February 2026). ESR1 was represented by the human estrogen receptor α ligand-binding domain structure (PDB ID: 2BJ4; X-ray diffraction; resolution: 2.00 Å), MTOR by the FKBP-rapamycin binding domain of mTOR/FRAP (PDB ID: 1AUE; X-ray diffraction; resolution: 2.33 Å), and PIK3CA by the human PI3K p110α C2 domain solution structure (PDB ID: 2ENQ; solution NMR; resolution not applicable). For PIK3CA, the best representative conformer, model 1, was used for receptor preparation [17]. Three-dimensional conformations of the small-molecule ligands were generated from their SMILES strings using the NCI/CADD online server (https://cactus.nci.nih.gov/, accessed on 2 February 2026). To ensure structural accuracy, the ligand geometries were cross-validated with the ChemSpider database (https://www.rsc.org/, accessed on 2 February 2026) and aligned with structures in PyMOL 2.6. Prior to docking, all receptor proteins were preprocessed in PyMOL 2.6 to remove water molecules, native ligands, and non-essential ions. Energy minimization of the small-molecule ligands was performed using the MMFF94 force field implemented in Chem3D 22.0.0 software (10,000 iterations; RMSD = 0.001) to obtain stable, low-energy conformations.

Semi-flexible molecular docking was subsequently carried out using AutoDock Vina 1.1.2. The docking regions were defined as domain-associated predicted binding pockets, and the detailed grid box parameters, including center coordinates, box dimensions, and grid spacing, are provided in Supplementary Tables S1.1 and S1.2. Native-ligand re-docking was considered for docking-protocol validation when an appropriate co-crystallized small-molecule ligand was available. However, this validation was not uniformly applicable to the three selected receptor structures because MTOR/1AUE and PIK3CA/2ENQ did not contain suitable co-crystallized small-molecule inhibitors for direct native-ligand re-docking. Therefore, the docking results in this study were interpreted as computationally predicted binding modes rather than experimentally validated binding events. For each ligand-receptor pair, 20 independent docking runs were executed, and the conformation with the lowest binding free energy was selected as the optimal binding pose [18]. The three complexes exhibiting the highest binding affinities were chosen, and their interaction patterns (e.g., hydrogen bonds, hydrophobic interactions) were visualized in three dimensions using PyMOL 2.6.

SwissADME is an online platform that analyzes physicochemical properties, pharmacokinetics, and drug-likeness of molecules by calculating relevant parameters [19]. The ADME properties of dauricine as the candidate active compound were evaluated using this platform.

### 2.10. Molecular Dynamics Simulation and MM/PBSA Binding Free Energy Calculation

Molecular dynamics simulations were used to further assess and refine the predicted binding modes between these small molecules and protein receptors, thereby providing a more precise understanding at the molecular level [20]. To gain deeper insights into the dynamic binding behavior and interaction stability between core active compounds and their target proteins, molecular dynamics (MD) simulations were performed on the three complexes exhibiting the highest binding affinities from molecular docking. The simulations were initiated using the processed conformations obtained in the previous step and carried out with the GROMACS 2025 software package [21]. The protein atoms were described by the AMBER99SB-ILDN force field [22,23], while the topology parameters for the small-molecule ligands were generated using the GAFF2 force field [24]. The topology files of the protein and ligand were merged to construct the complete complex system, which was then placed in a cubic periodic box with a minimum distance of 1.2 nm between the complex and the box boundaries. The system was solvated with the TIP3P water model [25], and Na^+^/Cl^−^ ions were added to neutralize the net charge and achieve an ionic concentration of 0.15 M, thereby mimicking physiological conditions.

Prior to the production run, energy minimization was conducted using the steepest descent method for 1000 steps to eliminate unfavorable atomic contacts. The system was then equilibrated under the NVT ensemble for 1 ns at 310 K using a V-rescale thermostat (coupling constant = 0.1 ps), followed by an additional 1 ns equilibration under the NPT ensemble at 310 K and 1 bar pressure (using a Berendsen pressure bath with a coupling constant of 0.2 ps). Subsequently, a 100 ns production MD simulation was performed under the NPT ensemble, with temperature and pressure maintained by the V-rescale thermostat (coupling constant 0.1 ps) and a Parrinello-Rahman pressure bath (coupling constant 0.1 ps), respectively. An integration time step of 2 fs was used, and trajectories were saved every 10 ps for subsequent analysis. Key structural parameters, including root-mean-square deviation (RMSD), radius of gyration (Rg), solvent-accessible surface area (SASA), and the number of hydrogen bonds, were calculated from the simulation trajectories [26]. Data analysis and visualization were performed using Origin software (version 10.1.5.132).

To quantitatively evaluate the binding strength between the small molecules and target proteins, the binding free energy for each complex was calculated using the MM/PBSA method. This approach is widely employed to assess protein-ligand interaction affinities, with more negative values indicating more stable binding. The calculations were based on the 100 ns MD trajectories and performed according to the standard protocol [27]. The total binding free energy was decomposed into van der Waals energy, electrostatic energy, polar solvation energy, and non-polar solvation energy components.

## 3. Results

### 3.1. Identification of Common Targets Between Predicted Drug Targets and Hypoxia-Related Genes

**Figure 1:**
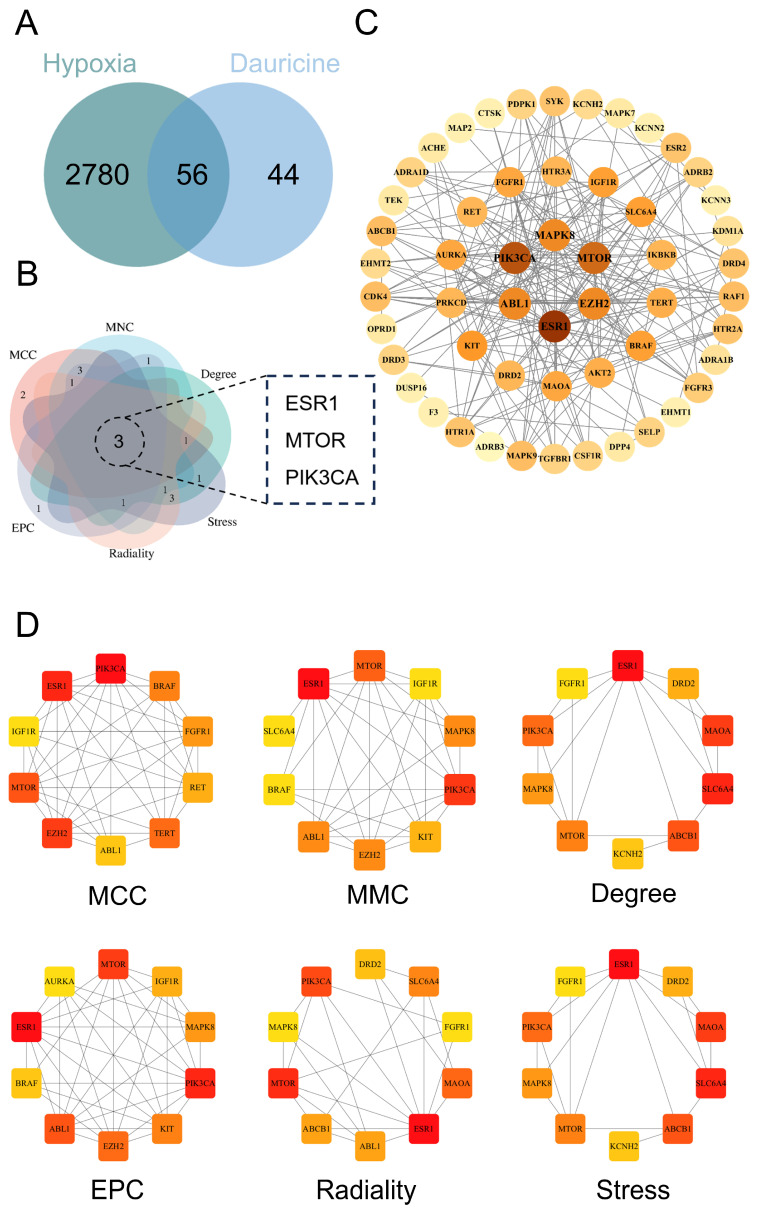
Screening of common targets and core targets. (**A**) Common targets between drug components and disease-related targets. (**B**) Identification of core targets using six algorithms based on the CytoHubba. (**C**) PPI network of common targets. (**D**) The top 10 core targets identified through screening with six algorithms.

Using the SMILES string of the compound dauricine, potential protein targets were predicted via the SwissTargetPrediction platform. A total of 100 candidate targets were obtained. Subsequently, hypoxia-related genes were retrieved from multiple disease databases using “hypoxia” as the keyword. These databases included PharmGKB, OMIM, GeneCards (with a relevance score ≥ 0.55), and DisGeNET. In total, 2836 hypoxia-associated genes were collected. For this purpose, the GeneCards database serves as a comprehensive online human gene compendium. It integrates and links information on human genes, diseases, proteins, and cellular functions. To identify common targets, an intersection analysis was performed using the online tool jvenn. The results revealed 56 overlapping genes between the predicted dauricine targets and the hypoxia-related gene set (Figure 1A, Table 1).

It should be noted that AKT1, although recognized as a canonical component of the PI3K-Akt pathway and an important regulator of hypoxia-related responses, was not retained in the final overlapping target set because it was not predicted as a potential dauricine-associated target under the current SwissTargetPrediction screening conditions. Therefore, AKT2, rather than AKT1, appeared in the 56 common targets.

### 3.2. The Identification of Core Targets in PPI Network

Based on these 56 common targets, a PPI network was constructed using the STRING database, with the species set to “*Homo sapiens*” and a confidence score ≥ 0.4, yielding 55 nodes and 226 edges, with an average node degree of 8.22 (Figure 1C). We employed 6 algorithms from Cytohubba plug-in within Cytoscape 3.8.0 software to further filter the core targets from PPI network. As shown in Figure 1D, we selected the top 10 targets from the 6 algorithms and acquired their overlapping targets using a Venn diagram. In Figure 1C, nodes with distinct features represent protein targets. Both node size and color intensity are positively correlated with the degree of connectivity (degree) in the PPI network: larger and darker nodes indicate more interactions with other proteins, a more central topological position, and a stronger functional hub role. The degree values were derived from topological analysis using CytoNCA. In the visualization layout, degree values decrease progressively from the inner to the outer nodes. Similarly, in Figure 1D, the gradient of node colors from light yellow to dark red reflects the distribution of degree values.

Ultimately, the intersection of the core topological targets and core functional targets yielded three final core targets: ESR1, PIK3CA, and MTOR (Figure 1B). The hypoxia relevance of these candidate targets was further cross-checked using the iHypoxia database. ESR1, MTOR, and PIK3CA all showed hypoxia-responsive evidence, mainly from high-throughput transcriptomic datasets, with additional low-throughput or genomic adaptation records for MTOR and PIK3CA. Their expression changes were not uniformly directional but varied across cell types, tissues, hypoxic conditions, and experimental platforms. These results support their relevance to hypoxia biology while indicating a context-dependent response pattern.

### 3.3. GO and KEGG Enrichment and Subsequent Network Analysis

**Figure 2:**
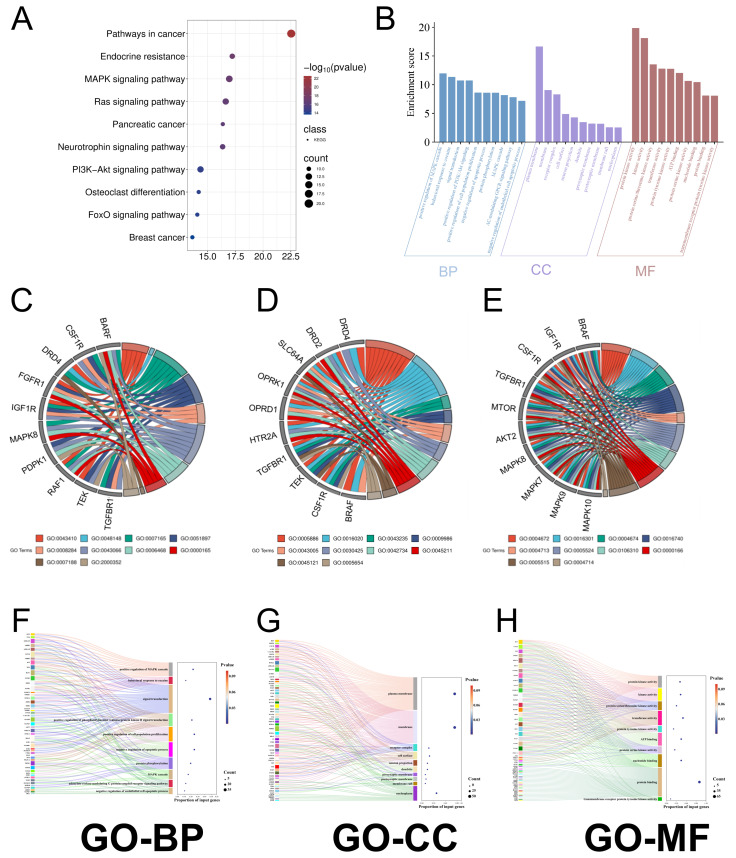
GO and KEGG enrichment analysis of 56 common target genes. (**A**) Top 10 KEGG pathways based on *p*-value. (**B**) Top 10 terms in BP, CC, and MF based on *p*-value. (**C**–**E**) Genes involved in the regulation of BP, CC, and MF. (**F**–**H**) Key targets associated with the top 10 entries in BP, CC, and MF, and the number of shared genes per entry.

**Figure 3:**
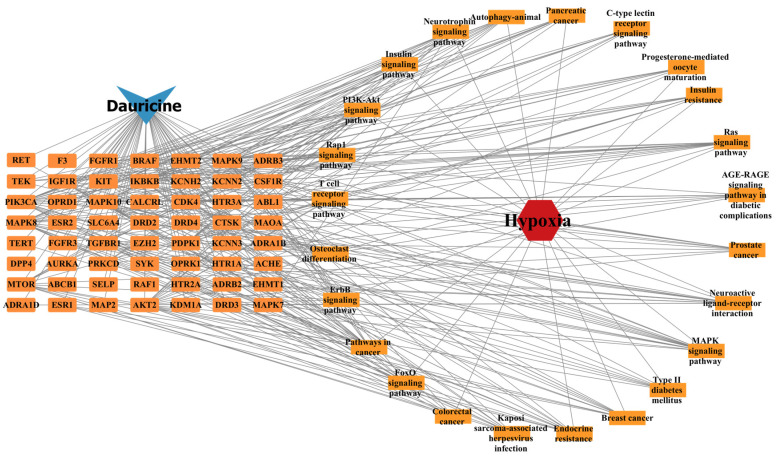
Active component-target-pathway-disease network.

GO and KEGG enrichment analyses of the 56 targets revealed their significant involvement in biological processes, cellular components, and molecular functions. KEGG analysis highlighted 180 significantly enriched pathways, among which the MAPK and PI3K-Akt signaling pathways were predominant in hypoxia adaptation (Figure 2). Notably, these genes were mainly localized to the plasma membrane and associated structures, as well as the nucleoplasm. In terms of molecular function, they were primarily involved in protein kinase activity and nucleotide binding, which are essential for hypoxic signal transduction. A component-target-pathway-disease network was then constructed and subjected to topological analysis using CytoNCA (Figure 3). In this network, core targets are represented by dark orange nodes arranged in a matrix on the left, while significantly enriched pathways are depicted as light orange nodes arranged in a circular pattern on the right. These findings collectively suggest that dauricine exerts its therapeutic effects by modulating multiple biological functions and signaling pathways.

### 3.4. GeneMANIA Functional Association (GMFA) Network Analysis of Three Core Targets

**Figure 4:**
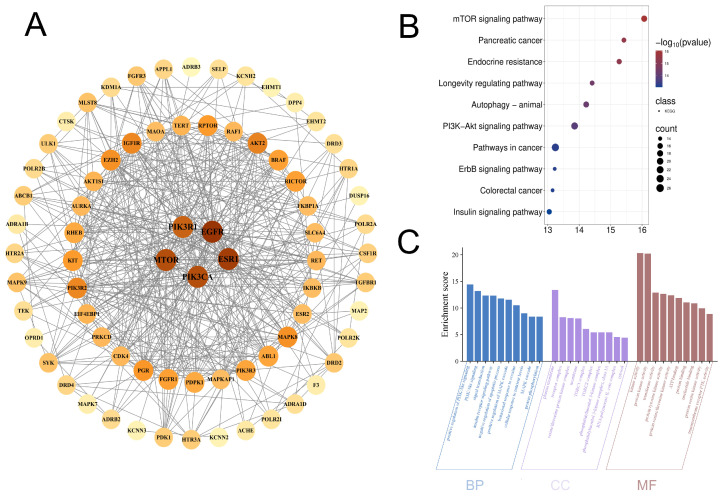

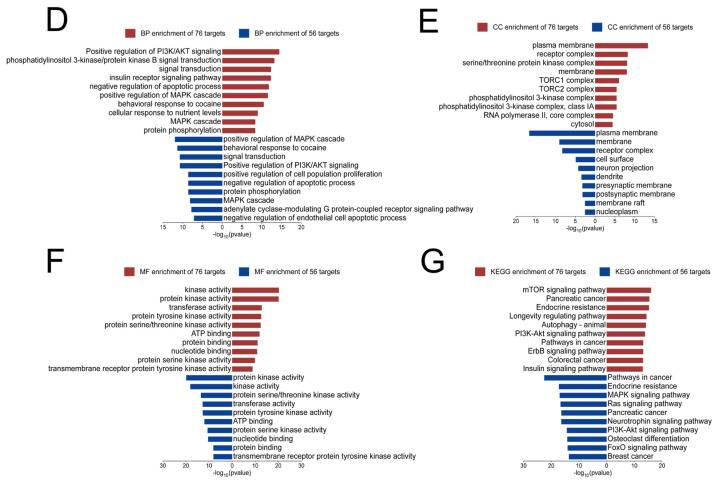
GO and KEGG enrichment analysis of the expanded core target dataset. (**A**) The PPI network of the expanded core target dataset. (**B**) The top 10 KEGG pathways based on *p*-value. (**C**) The top 10 entries in BP, CC, and MF based on *p*-value. (**D**–**F**) The comparison of BP, CC, and MF between the 35 hub targets and the expanded target dataset. (**G**) The comparison of KEGG enrichment analysis between the 35 hub targets and the expanded target dataset.

To obtain a more comprehensive functional profile, a GeneMANIA-based functional association network was generated for the three core genes, expanding to the top 10 related genes for each input gene, resulting in a combined set of 76 targets (Figure 4A,C–G). Subsequent GO and KEGG analyses of this expanded set reinforced enrichment in similar biological themes and pathways, particularly the mTOR and PI3K-Akt signaling pathways (Figure 4B), with detailed pathway diagrams provided in Supplementary Figures S1–S3.

### 3.5. ADME and Toxicity Evaluation of Dauricine

Before molecular docking analysis, the drug-likeness and toxicity profiles of dauricine were evaluated to assess its preliminary pharmacokinetic suitability as a candidate compound. Lipinski’s Rule of Five, a classical criterion for estimating oral bioavailability, requires that a compound violate no more than one of its five principles. As shown in Table 2, dauricine has a molecular weight of 624.32 Da, which marginally exceeds the recommended upper limit of 500 Da. However, its lipophilicity, hydrogen bond donors, and hydrogen bond acceptors complied with the remaining Lipinski criteria, indicating that its hydrophile-lipophile balance and polar group distribution remain within a generally acceptable range for membrane permeability. In addition, toxicity prediction showed no predicted drug-induced liver injury, carcinogenicity, hERG inhibition, mitochondrial toxicity, oxidative stress liability, endocrine disruption, or nephrotoxicity (Table 3). Overall, these results support the preliminary drug-likeness and safety profile of dauricine, although further experimental pharmacokinetic and toxicological validation is still required.

### 3.6. Molecular Docking Analysis of Dauricine with Core Hypoxia-Related Targets

**Figure 5:**
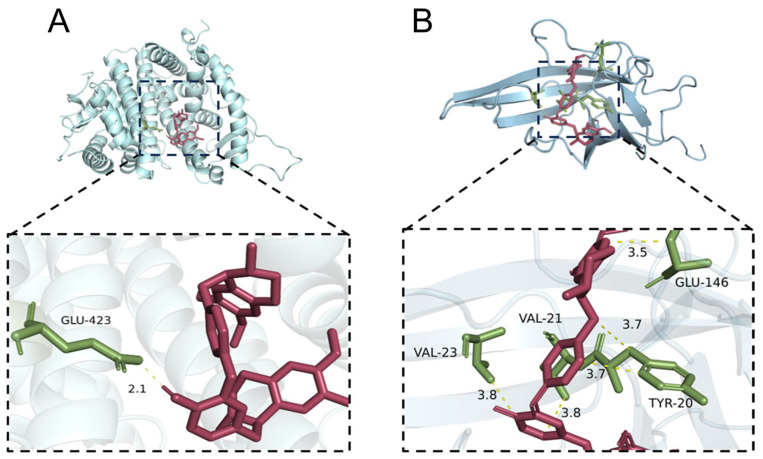

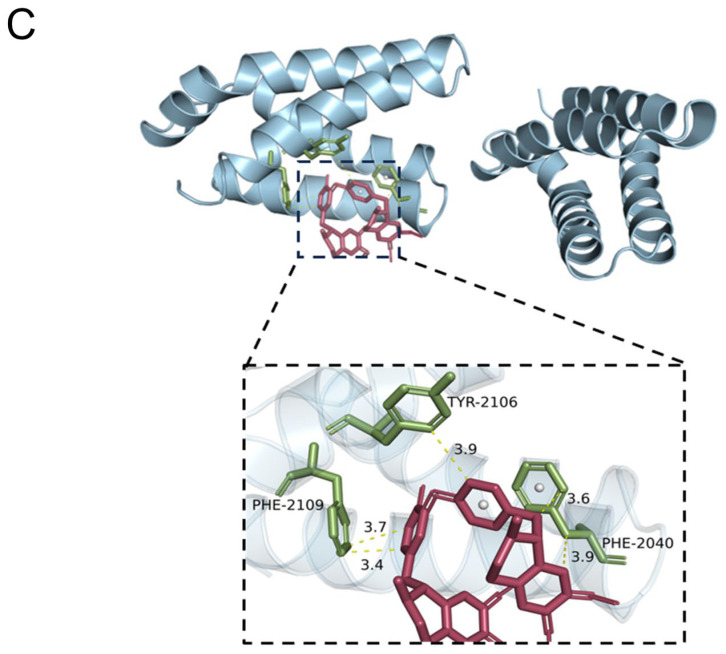
Molecular docking visualization results of dauricine with the selected target structures. (**A**) Predicted docking pose of dauricine in the ESR1 ligand-binding-domain-associated pocket. (**B**) Predicted docking pose of dauricine in the PIK3CA C2-domain-associated pocket. (**C**) Predicted docking pose of dauricine in the MTOR FRB-domain-associated pocket.

To evaluate the binding potential between the active compound dauricine and the core hypoxia-related targets identified through network pharmacology, molecular docking studies were performed. According to established criteria for evaluating binding energy, the stability of receptor-ligand binding is inversely correlated with the binding energy value; lower binding energies correspond to more stable conformations. Generally, a binding energy below −5.0 kcal/mol is considered the threshold for favorable receptor-ligand binding stability [28]. As shown in Table 4, dauricine exhibited binding energies significantly below this threshold for all three core targets: −7.2 kcal/mol for ESR1, −6.8 kcal/mol for MTOR, and −7.0 kcal/mol for PIK3CA (Figure 5). These results suggest that dauricine can bind to the predicted domain-associated binding pockets of the selected target structures, forming energetically favorable docking conformations. Notably, the complexes formed with ESR1 and PIK3CA displayed more favorable predicted binding energies, suggesting stronger computationally estimated binding potential for these two targets. To further characterize the predicted binding modes, residue-level protein–ligand contact analyses were performed for the three docking complexes. The interacting pocket residues, closest protein and ligand atoms, minimum heavy-atom distances, and predicted contact types are summarized in Supplementary Tables S2–S4. It should be noted that the distance labels shown in Figure 5 correspond to visualized interaction distances generated during docking visualization, whereas Supplementary Tables S2–S4 report calculated minimum heavy-atom distances from the best-scoring docking poses; therefore, minor numerical differences between the figure labels and Supplementary Tables are expected and do not affect the interpretation of residue-level contacts. These analyses provide residue-level support for the predicted binding poses of dauricine within the ESR1 ligand-binding-domain-associated pocket, the MTOR FRB-domain-associated pocket, and the PIK3CA C2-domain-associated pocket.

### 3.7. Assessment of Complex System Stability via Molecular Dynamics Simulations

To further assess the predicted binding modes observed in molecular docking, 100 ns molecular dynamics simulations were performed on the three top-scoring complexes (ESR1/dauricine, MTOR/dauricine, and PIK3CA/dauricine) to evaluate their dynamic behavior and binding stability under physiological conditions.

**Figure 6:**
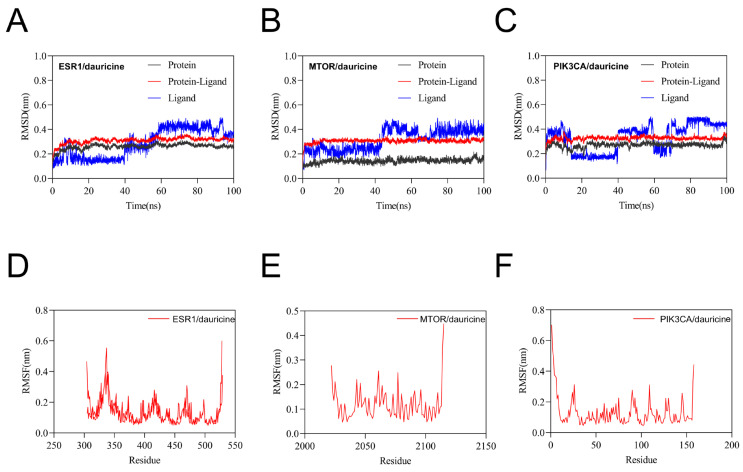

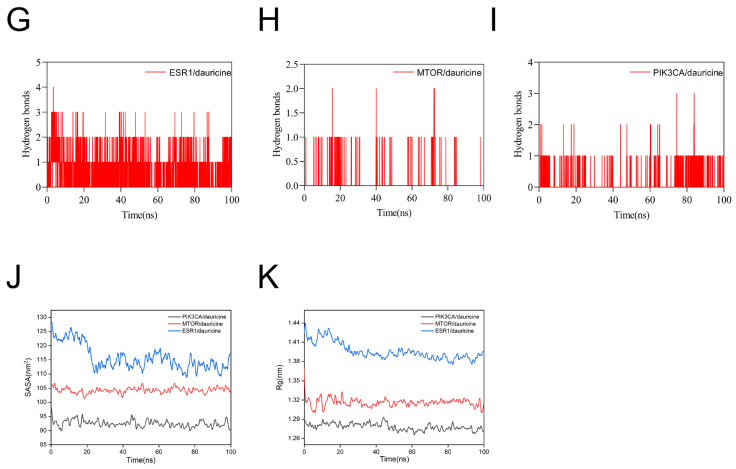
MD simulation results of the dauricine-target complex. (**A**–**C**) Root-mean-square deviation (RMSD) analysis of ESR1/dauricine, MTOR/dauricine, and PIK3CA/dauricine complexes during molecular dynamics simulations. (**D**–**F**) Root-mean-square fluctuation (RMSF) of protein residues in the ESR1/dauricine, MTOR/dauricine, and PIK3CA/dauricine complexes, reflecting the flexibility of specific regions. The y-axis represents the RMSF value (in nm), indicating the average fluctuation amplitude of each residue’s atomic positions during the simulation. (**G**–**I**) Number of hydrogen bonds formed between dauricine and ESR1, MTOR, and PIK3CA during the MD simulation, reflecting the hydrogen-bonding interaction patterns of the ligand-protein complexes. The y-axis represents the number of hydrogen bonds formed between the ligand and the protein over the course of the simulation. (**J**) Solvent-accessible surface area (SASA). The y-axis reflects the surface area of the protein exposed to the solvent. (**K**) Radius of gyration (Rg) of the complex, which measures protein compactness—lower values indicate tighter protein folding.

**Figure 7:**
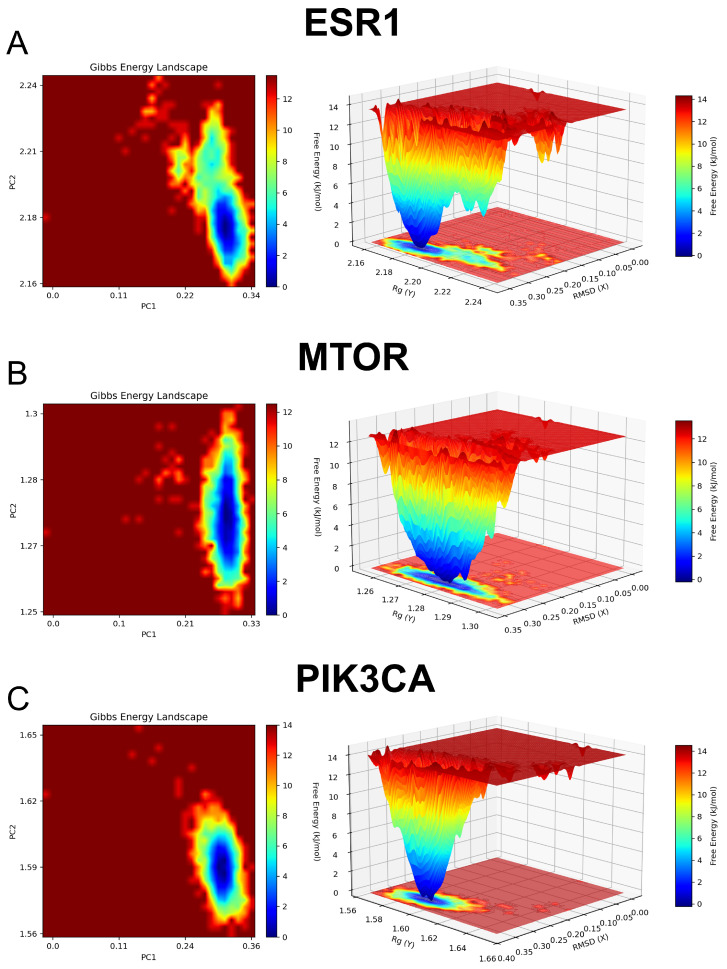
MD simulation results of dauricine-target complex. (**A**–**C**) Gibbs free energy landscapes of ESR1, MTOR and PIK3CA in complex with dauricine. The dark blue regions represent energy troughs, corresponding to high-probability conformational clusters that indicate stable states of the protein-ligand system. The yellow/red regions represent energy plateaus or saddle points, indicating transition states or energy barriers that separate distinct conformational clusters and reflect the kinetic bottlenecks of conformational transitions.

Root-mean-square deviation (RMSD) values are used to assess whether the simulation system has reached a stable state, with RMSD values within 1 nm indicating the relative stability of the protein-ligand interaction under physiological conditions [29]. RMSD analysis of the backbone atoms (Figure 6A–C) demonstrated that all three complexes reached stable equilibrium within 10 ns, with subsequent RMSD values remaining within a narrow fluctuation range of 0.2–0.3 nm. This indicates that the protein-ligand complexes converged to equilibrium conformations without significant conformational drift, suggesting relatively stable binding. Root-mean-square fluctuation (RMSF) profiles (Figure 6D–F) revealed that the majority of residues exhibited RMSF values below 0.2 nm, indicating overall rigidity of the protein backbones, with local flexibility observed only in loop regions. The radius of gyration (Rg) and solvent-accessible surface area (SASA) remained generally consistent throughout the simulations (Figure 6J,K), with a decreasing trend observed after 10 ns, confirming that dauricine binding maintained the compact folding state of the proteins and stability of the solvent environment. Molecular docking and molecular dynamics simulations provided multi-faceted computational support for the predicted interactions between dauricine and its core targets. Hydrogen bond analysis (Figure 6G–I) showed different interaction patterns among the three complexes. Dauricine formed more frequent hydrogen bonds with ESR1 and PIK3CA, whereas only 0–1 hydrogen bonds were observed for the MTOR/dauricine complex during most of the simulation, indicating relatively weak and transient hydrogen-bond interactions in this system. Therefore, the stability of the MTOR/dauricine complex is unlikely to be primarily driven by hydrogen bonding, but may instead be mainly supported by non-hydrogen-bond interactions, particularly van der Waals and hydrophobic contacts, as further suggested by the MM/PBSA energy decomposition results. Gibbs free energy landscapes constructed based on principal component analysis (Figure 7A–C) displayed a single, deep global energy minimum (dark blue region) for all three complex systems, corresponding to a unique stable conformational cluster and indicating overall relative dynamic stability supported by multiple MD descriptors of the systems.

**Figure 8:**
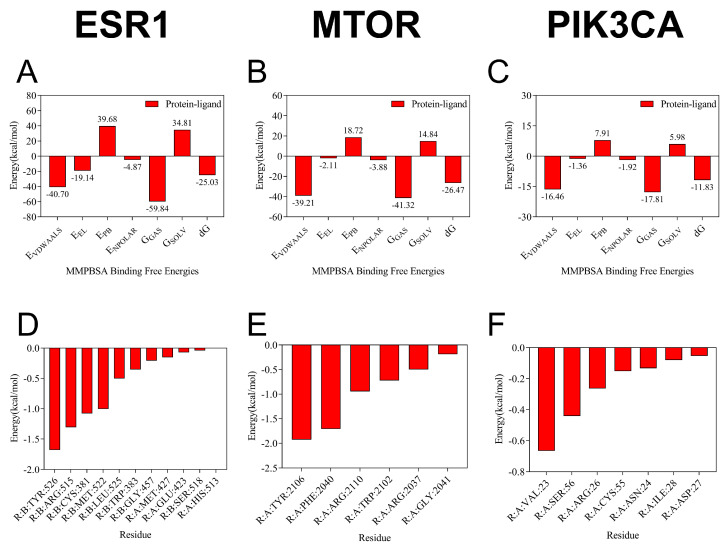
Molecular dynamics simulation results of dauricine-target complex. (**A**–**C**) Binding free energies and individual energy contributions for dauricine with ESR1, MTOR, and PIK3CA calculated using the MM/PBSA method. Negative values indicate favorable interactions. (**D**–**F**) Residue dissociation energies of dauricine with ESR1, MTOR, and PIK3CA complexes. It reflects the energy contribution of interactions between proteins and ligands. The y-axis represents energy. Negative values indicate that the residues stabilize ligand binding.

Binding free energies calculated using the MM/PBSA method (Table 5) demonstrated favorable binding affinities for all complexes. It is well established that the more negative the binding free energy (ΔG°), the more stable the formed complex [30]. The total binding free energies (ΔG_Total) for ESR1/dauricine, MTOR/dauricine, and PIK3CA/dauricine were −25.03 kcal/mol, −26.47 kcal/mol, and −11.83 kcal/mol, respectively. Energy decomposition analysis (Figure 8A–C) revealed that van der Waals interactions (ΔG_vdw) served as the primary driving force for binding, contributing the most favorable energy across all systems. Non-polar solvation energy (ΔG_enpol) also substantially contributed to binding, while polar solvation energy (ΔG_epb) exhibited unfavorable contributions that were numerically compensated by van der Waals and electrostatic interactions.

Key residue contribution analysis (Figure 8D–F) further demonstrated that van der Waals interactions constitute the major binding driving force. Specifically, in the ESR1/dauricine and MTOR/dauricine complexes, residues TYR-526 and TYR-2106 contributed significantly to stabilization (−1.68 and −1.92 kcal/mol, respectively). In the PIK3CA/dauricine complex, residues VAL-23, SER-56, and ARG-26 exerted major stabilizing roles. These residues provide persistent anchoring forces for the ligand through hydrophobic contacts or polar side chain interactions, thereby maintaining overall binding stability during periods of dynamic hydrogen bond fluctuations.

Collectively, these dynamics-derived results suggest that the predicted interactions between dauricine and ESR1, MTOR, and PIK3CA are supported by favorable binding affinity, dynamic behavior, energy distribution, and residue-level contributions, further supporting the potential of dauricine as a candidate compound for modulating hypoxia adaptation.

## 4. Discussion

Hypoxia serves as a common pathological foundation for numerous diseases and has consistently remained a core focus in both mechanistic exploration and novel drug development. Among the critical pathways involved, the PI3K-AKT signaling axis functions as a central hub through which cells sense and adapt to hypoxic environments, participating extensively in diverse pathophysiological processes ranging from metabolic reprogramming to the regulation of cell survival. In a hypoxic cell model of non-small cell lung cancer (NSCLC), exposure to hypoxia was shown to activate the PI3K-AKT signaling pathway, subsequently inducing cellular autophagy. Knockdown of the upstream regulatory factor EIF2AK3 suppressed PI3K-AKT phosphorylation [31]. Concurrently, the MAPK signaling pathway constitutes a core transduction cascade in cellular responses to hypoxic stress. Renal ischemia–reperfusion injury (IRI) represents a major cause of acute kidney injury. In a rat model of renal IRI, ischemia combined with hypoxia and subsequent reoxygenation markedly activated the MAPK signaling pathway, leading to upregulated expression of NADPH oxidase, enhanced production of reactive oxygen species (ROS), and increased release of inflammatory cytokines, thereby exacerbating renal tubular damage [32]. Natural products derived from herbs and plants have long served as invaluable sources for drug discovery. These compounds are distinguished by their multi-pathway, multi-target mechanisms of action and favorable safety profiles, rendering them suitable for prolonged therapeutic use [33]. As a natural compound derived from traditional Chinese medicine, dauricine exhibits favorable pharmacokinetic properties and multi-target regulatory capabilities, demonstrating considerable potential for applications in oncology. However, its therapeutic value in hypoxia-related diseases remains largely unexplored.

The rapid advancement of bioinformatics has positioned network pharmacology as a powerful tool for the rapid screening of bioactive compounds and the investigation of their underlying pharmacological mechanisms. Its holistic and systematic approach, which aligns closely with the principles of traditional Chinese medicine (TCM) prescriptions, has led to its widespread adoption in TCM research [34]. In the present study, the approach was employed to elucidate the multi-target, multi-pathway molecular mechanisms underlying the intervention of dauricine in hypoxic disorders, using chronic mountain sickness as a representative condition. Through the construction of a comprehensive “compound–target–pathway–disease” multidimensional network, ESR1, PIK3CA, and MTOR were identified as core targets. Their relevance to hypoxia was subsequently cross-checked and supported by evidence from the iHypoxia database. Enrichment analyses further indicated that the PI3K-Akt, MAPK, and mTOR signaling pathways serve as key mediators of dauricine’s pharmacological activity.

The identification of ESR1 as a core target should be interpreted cautiously, as ESR1 has not yet been experimentally confirmed as a direct target of dauricine. Nevertheless, ERα signaling has been reported to directly interact with the HIF-1 pathway, indicating that ESR1 may be linked to hypoxia-responsive transcriptional regulation rather than representing an isolated estrogen-related signal [35]. More importantly, estrogen/ERα signaling is known to crosstalk with the PI3K/AKT and MAPK pathways, and estrogen-induced activation of the PI3K/AKT-HIF-1-VEGF axis has been reported in previous studies [36]. In pulmonary vascular cells, ERα-mediated effects have also been associated with Akt and MAPK signaling, providing a potential mechanistic bridge between ESR1 and hypoxia-related vascular remodeling [37]. Although dauricine does not possess an obvious estrogen-like scaffold, the flexible ligand-binding pocket of ERα can accommodate structurally diverse ligands [38]. In addition, dauricine has previously been shown to suppress HIF-1α accumulation and VEGF expression through interference with the PI3K/AKT/mTOR pathway [8]. Therefore, ESR1 should be regarded as a computationally supported candidate target rather than a confirmed primary anti-hypoxia target. Taken together, the ERα–HIF-1/PI3K-Akt/mTOR crosstalk is supported by existing literature, whereas the specific involvement of ESR1 in dauricine-mediated hypoxia adaptation should be considered a novel computational prediction that requires direct experimental validation.

The absence of AKT1 from the overlapping target list further highlights a methodological limitation of database-dependent network pharmacology. Biologically important pathway nodes may be excluded if they are not predicted as compound-associated targets by the selected target prediction platform. Therefore, the present findings should be interpreted as computationally prioritized candidates rather than a complete representation of all hypoxia-related regulatory nodes.

Molecular docking and molecular dynamics simulations provided multi-faceted computational support for the predicted interactions between dauricine and its core targets. Binding energy calculations demonstrated that dauricine exhibits favorable binding affinities toward ESR1, PIK3CA, and MTOR, all exceeding the established threshold of −5.0 kcal/mol. One hundred-nanosecond molecular dynamics simulations, coupled with MM/PBSA calculations, confirmed the overall relative dynamic stability of all complexes: each system reached equilibrium within 10 ns, maintained RMSD fluctuations within a narrow range of 0.2–0.3 nm, and exhibited Gibbs free energy landscapes featuring a single, deep energy minimum—collectively affirming conformational stability and kinetic robustness. Notably, although the MTOR/dauricine complex exhibited fewer and more transient hydrogen bonds than the ESR1/dauricine and PIK3CA/dauricine complexes, its favorable MM/PBSA binding free energy and dominant van der Waals contribution suggest that its binding stability may be mainly maintained by hydrophobic and van der Waals interactions rather than persistent hydrogen bonding.

The ADME and toxicity evaluation provided preliminary support for the drug-likeness and safety profile of dauricine. Although dauricine slightly exceeded the classical molecular weight criterion in Lipinski’s Rule of Five, the remaining physicochemical properties and toxicity predictions were generally favorable. These computational ADME results should be interpreted as preliminary indicators and require further experimental pharmacokinetic and toxicological validation.

Collectively, these findings provide a theoretical foundation for understanding the potential molecular mechanisms by which dauricine regulates hypoxia-related targets, encompassing binding affinity, dynamic behavior, energetic drivers, and residue-level contributions. Nevertheless, the absence of direct experimental validation remains a major limitation of this study. The present work was designed as a computational mechanism-oriented study combining network pharmacology, molecular docking, molecular dynamics simulations, and MM/PBSA binding free energy calculations to prioritize potential targets and mechanistic pathways of dauricine in hypoxia regulation. Therefore, the identified targets and pathways should be interpreted as computationally supported candidates rather than experimentally confirmed mechanisms. In addition, native-ligand re-docking validation was not uniformly applicable to all selected receptor structures because suitable co-crystallized small-molecule ligands were unavailable for MTOR/1AUE and PIK3CA/2ENQ; thus, the docking poses should be interpreted as predictive models requiring further biochemical validation. Future studies using hypoxic cell models and, where feasible, animal models are required to validate the protective effects of dauricine and to determine whether ESR1, PIK3CA, MTOR, and the PI3K-Akt, MAPK, and mTOR signaling pathways are functionally regulated under hypoxic conditions. Potential validation experiments may include cell viability assays, ROS detection, HIF-1α expression analysis, and measurement of AKT/MTOR phosphorylation levels.

In conclusion, this study proposes a computationally supported multi-target mechanism by which dauricine may participate in hypoxia regulation. Through network pharmacology screening, ESR1, PIK3CA, and MTOR were prioritized as candidate core targets, and enrichment analyses highlighted the PI3K-Akt, MAPK, and mTOR signaling pathways as major hypoxia-related pathways potentially associated with dauricine activity. ADME and toxicity prediction suggested a generally acceptable preliminary drug-likeness and safety profile. Molecular docking, residue-level contact analysis, molecular dynamics simulations, and MM/PBSA binding free energy analysis further supported the predicted binding potential and dynamic stability of the dauricine-target complexes. Overall, these findings provide a computational basis for considering dauricine as a potential multi-target candidate for hypoxia intervention, while emphasizing the need for direct experimental validation in defined hypoxic models.

## Figures and Tables

**Table 1:** 56 common targets identified as potential targets for hypoxia.

56 Possible Protein Targets for Hypoxia							
MTOR	IGF1R	RAF1	DRD4	ABL1	SLC6A4	EHMT2	ABCB1
RET	OPRD1	AKT2	PDPK1	MAOA	TGFBR1	KCNH2	F3
TEK	ESR2	BRAF	OPRK1	ADRA1B	PRKCD	CDK4	FGFR1
PIK3CA	FGFR3	IKBKB	ADRB2	ACHE	HTR3A	MAPK7	MAPK10
MAPK8	AURKA	CALCRL	DRD3	ADRA1D	CTSK	ADRB3	SYK
TERT	SELP	DRD2	MAPK9	HTR1A	KCNN3	CSF1R	HTR2A
DPP4	MAP2	EZH2	KCNN2	EHMT1	ESR1	KIT	KDM1A

**Table 2:** Drug-likeness properties of dauricine.

Compound	MW	HBA	HBD	MLogP	Lipinski’s Violations	Bioavailability Score	TPSA
Dauricine	624.32	8	1	3.46	1	0.55	72.86

**Table 3:** Predicted toxicity profile of dauricine.

Toxicity Category	Assessment Endpoint	Prediction	Value/Probability
Cardiotoxicity	hERG Inhibition	Inactive	0.899
Carcinogenicity	Carcinogenicity	Inactive	0.59
Acute Toxicity	Oral LD50 (Rat)	Low Toxicity	1365 mg/kg
Mechanism-Based Toxicity	Mitochondrial Toxicity (MMP)	Inactive	0.86
	Oxidative Stress (ARE Pathway)	Inactive	0.98
	Endocrine Disruption (AR, ER, AhR)	Inactive	>0.90
Other Organ Toxicity	Nephrotoxicity	Inactive	0.56

**Table 4:** Molecular docking binding energies of dauricine with core targets.

Targets	Ligand	Binding Affinity
ESR1	dauricine	−7.2
MTOR	dauricine	−6.8
PIK3CA	dauricine	−7.0

**Table 5:** Binding free energy of the three complex systems.

Energy Component	ESR1-Dauricine	MTOR-Dauricine	PIK3CA-Dauricine
ΔG_vdw_	−40.7	−39.21	−16.46
ΔG_eel_	−19.14	−2.11	−1.36
ΔG_epb_	39.68	18.72	7.91
ΔG_enpol_	−4.87	−3.88	−1.92
ΔG_Total_	−25.03	−26.47	−11.83

## Data Availability

The original contributions presented in this study are included in the article/Supplementary Material. Publicly available databases and online tools used in this study are described in the Section 2. Further inquiries can be directed to the corresponding authors.

## Supplementary Materials

The following supporting information can be downloaded at: https://www.mdpi.com/article/10.3390/cimb48060550/s1.

## References

[1] Ulloa N.A., Cook J. (2024). Altitude-Induced Pulmonary Hypertension.

[2] Zhou M., Liu X., Li L. (2023). Secondary polycythaemia from chronic hypoxia is a risk for cerebral thrombosis: A case report. BMC Neurol..

[3] Liu W.L., Zhang X.R., Liu J.H., Pu L.L., Ai L.L., Xu H.B., Wang G.R., Wang D., Song X.N., Zhang Y.N. (2025). An erythroid-biased FOShi hematopoietic multipotent progenitor subpopulation contributes to adaptation to chronic hypoxia. Cell Stem Cell.

[4] Liao Y.H., Li C.I., Lin C.C., Lin J.G., Chiang J.H., Li T.C. (2017). Traditional Chinese medicine as adjunctive therapy improves the long-term survival of lung cancer patients. J. Cancer Res. Clin. Oncol..

[5] Hou S.X., Dai Z.S., Wang F.J., Guo L.J., Hu C.J. (1982). Studies on the pharmacokinetics of dauricine in vivo. Acta Pharm. Sin..

[6] Chen K.Q., Wang S.Z., Lei H.B., Liu X. (2024). Dauricine: Review of Pharmacological Activity. Drug Des. Dev. Ther..

[7] Chen S.H., Hu C.J. (1981). Antihypertensive effect of dauricine and preliminary analysis of its mechanism. Chin. Tradit. Herb. Drugs.

[8] Tang X.D., Zhou X., Zhou K.Y. (2009). Dauricine inhibits insulin-like growth factor-I-induced hypoxia inducible factor 1α protein accumulation and vascular endothelial growth factor expression in human breast cancer cells. Acta Pharmacol. Sin..

[9] Zhao L., Zhang H., Li N., Chen J., Xu H., Wang Y., Liang Q. (2023). Network pharmacology, a promising approach to reveal the pharmacology mechanism of Chinese medicine formula. J. Ethnopharmacol..

[10] Hopkins A. (2008). Network pharmacology: The next paradigm in drug discovery. Nat. Chem. Biol..

[11] Kim S.H., Chen J., Cheng T.J., Gindulyte A., He J., He S.Q., Li Q.L., Shoemaker B.A., Thiessen P.A., Yu B. (2025). Pubchem 2025 Update. Nucleic Acids Res..

[12] Szklarczyk D., Gable A.L., Lyon D., Junge A., Wyder S., Huerta-Cepas J., Simonovic M., Doncheva N.T., Morris J.H., Bork P. (2019). STRING v11 Protein-Protein Association Networks with Increased Coverage, Supporting Functional Discovery in Genome-Wide Experimental Datasets. Nucleic Acids Res..

[13] Shan Z., Zhang H., He C., An Y., Huang Y., Fu W., Wang M., Du Y., Xie J., Yang Y. (2024). High-Protein Mulberry Leaves Improve Glucose and Lipid Metabolism via Activation of the PI3K/Akt/PPARα/CPT-1 Pathway. Int. J. Mol. Sci..

[14] Tang D., Chen M., Huang X., Zhang G., Zeng L., Zhang G., Wu S.J., Wang Y.S. (2023). Rplot: A free online platform for data visualization and graphing. PLoS ONE.

[15] Liu Z.X., Wang P., Zhang Q., Li S., Zhang Y., Guo Y., Jia C., Shao T., Li L., Cheng H. (2023). iHypoxia: An Integrative Database of Protein Expression Dynamics in Response to Hypoxia in Animals. Genom. Proteom. Bioinform..

[16] Yu Y., Zhou M., Long X., Yin S., Hu G., Yang X., Jian W., Yu R. (2023). Study on the mechanism of action of colchicine in the treatment of coronary artery disease based on network pharmacology and molecular docking technology. Front. Pharmacol..

[17] Burley S.K., Bhikadiya C., Bi C., Bittrich S., Chen L., Crichlow G.V., Christie C.H., Dalenberg K., Costanzo L.D., Duarte J.M. (2021). RCSB Protein Data Bank: Powerful New Tools for Exploring 3D Structures of Biological Macromolecules for Basic and Applied Research and Education in Fundamental Biology, Biomedicine, Biotechnology, Bioengineering and Energy Sciences. Nucleic Acids Res..

[18] Kim S., Thiessen P.A., Bolton E.E., Chen J., Fu G., Gindulyte A., Han L.Y., He J., He S.Q., Shoemaker B.A. (2016). PubChem Substance and Compound databases. Nucleic Acids Res..

[19] Daina A., Michielin O., Zoete V. (2017). SwissADME: A free web tool to evaluate pharmacokinetics, drug-likeness and medicinal chemistry friendliness of small molecules. Sci. Rep..

[20] Bai G., Pan Y., Zhang Y., Li Y., Wang J., Wang Y., Teng W., Jin G., Geng F., Cao J. (2023). Research advances of molecular docking and molecular dynamic simulation in recognizing interaction between muscle proteins and exogenous additives. Food Chem..

[21] Abraham M.J., Murtola T., Schulz R., Páll S., Smith J.C., Hess B., Lindahl E. (2015). Gromacs: High performance molecular simulations through multi-level parallelism from laptops to supercomputers. SoftwareX.

[22] Lindorff-Larsen K., Piana S., Palmo K., Maragakis P., Klepeis J.L., Dror R.O., Shaw D.E. (2010). Improved side-chain torsion potentials for the Amber ff99SB protein force field. Proteins.

[23] Hornak V., Abel R., Okur A., Strockbine B., Roitberg A., Simmerling C. (2006). Comparison of multiple Amber force fields and development of improved protein backbone parameters. Proteins.

[24] Wang J., Wolf R.M., Caldwell J.W., Kollman P.A., Case D.A. (2004). Development and testing of a general amber force field. J. Comput. Chem..

[25] William L.J., Jayaraman C., Jeffry D.M., Roger W.I., Michael L.K. (1983). Comparison of simple potential functions for simulating liquid water. J. Chem. Phys..

[26] Valdés-Tresanco M.S., Valdés-Tresanco M.E., Valiente P.A., Moreno E. (2021). gmx_MMPBSA: A New Tool to Perform End-State Free Energy Calculations with GROMACS. J. Chem. Theory Comput..

[27] Jayashree S., Thomas E.C., Piotr C., Peter A.K., David A. (1998). Continuum Solvent Studies of the Stability of DNA, RNA, and Phosphoramidate-DNA Helices. J. Am. Chem. Soc..

[28] Li Y., Li X.B., Cao X.J. (2022). Exploring the mechanism of Erxian Decoction in the treatment of premature ovarian failure based on network pharmacology and molecular docking. J. Hainan Med. Univ..

[29] Sarker P., Mitro A., Hoque H., Hasan N., Jewel G.N.A. (2023). Identification of potential novel therapeutic drug target against Elizabethkingia anophelis by integrative pan and subtractive genomic analysis: An in silico approach. Comput. Biol. Med..

[30] Marciniak A., Kotynia A., Szkatuła D., Krzyżak E. (2022). The 2-hydroxy-3-(4-aryl-1-piperazinyl) propyl Phthalimide Derivatives as Prodrugs—Spectroscopic and Theoretical Binding Studies with Plasma Proteins. Int. J. Mol. Sci..

[31] Fu J., Xu W., Wang G., Zeng L., Xian L., Wei Y., Zhang J. (2026). Hypoxia-triggered autophagy modulates cisplatin resistance in non-small cell lung Cancer via EIF2AK3-dependent PI3K/AKT signaling and mTOR-independent mechanisms. Cell Death Discov..

[32] Pei J., Zhang J., Yu C., Luo J., Wen S., Hua Y., Wei G. (2026). Selenoprotein GPX3 regulates NADPH oxidase expression by inhibiting the MAPK signaling pathway and thereby attenuating the inflammatory response in renal ischemia-reperfusion injury. Genes Dis..

[33] Bungãu S.G., Popa V.C. (2024). Between Religion and Science: Some Aspects: Concerning Illness and Healing in Antiquity. Transylv. Rev..

[34] Niu B., Xie X., Xiong X., Jiang J. (2022). Network Pharmacology-Based Analysis of the Anti-Hyperglycemic Active Ingredients of Roselle and Experimental Validation. Comput. Biol. Med..

[35] Yang J., AlTahan A., Jones D.T., Buffa F.M., Bridges E., Interiano R.B., Qu C., Vogt N., Li J.L., Baban D. (2015). Estrogen receptor-α directly regulates the hypoxia-inducible factor 1 pathway associated with antiestrogen response in breast cancer. Proc. Natl. Acad. Sci. USA.

[36] Kazi A.A., Molitoris K.H., Koos R.D. (2009). Estrogen Rapidly Activates the PI3K/AKTPathway and Hypoxia-Inducible Factor 1 and Induces Vascular Endothelial Growth Factor A Expressionin Luminal Epithelial Cells of the Rat Uterus. Biol. Reprod..

[37] Wright A.F., Ewart M.A., Mair K., Nilsen M., Dempsie Y., Loughlin L., Maclean M.R. (2015). Oestrogen receptor alpha in pulmonary hypertension. Cardiovasc. Res..

[38] Lee S., Barron M.G. (2017). Structure-Based Understanding of Binding Affinity and Mode of Estrogen Receptor α Agonists and Antagonists. PLoS ONE.

