# Supplementary material for: Dauricine Mitigates Hypoxia Through Targeting ESR1, PIK3CA, and MTOR: A Network Pharmacology and Molecular Dynamics Simulation Investigation
Source: Curr Issues Mol Biol. 2026 May 23;48(6):550. doi: 10.3390/cimb48060550 (PMC13297437; doi:10.3390/cimb48060550)
Supplement: Supplementary file 1 [file cimb-48-00550-s001.zip › cimb-4319076-supplementary/Supplementary File/Supplementary File--Additional Materials for Revision/Table/Supplementary Table S1.pdf]

Supplementary Table S1.1 Structural information of target proteins used for molecular docking.

Target	PDB ID	Method	Resolution	Chain / domain used
ESR1	2BJ4	X-ray diffraction	2.00 Å	Estrogen receptor α ligand-binding domain
MTOR	1AUE	X-ray diffraction	2.33 Å	MTOR/FRAP FKBP-rapamycin binding domain, FRB domain
PIK3CA	2ENQ	Solution NMR	Not applicable	PI3K p110α C2 domain

Note: This table summarizes the structural information of the receptor proteins used for molecular docking. ESR1 and MTOR were represented by X-ray crystallographic structures, whereas PIK3CA was represented by an NMR solution structure; therefore, crystallographic resolution is not applicable for PIK3CA.

Supplementary Table S1.2 Docking area and grid box parameters used for molecular docking.

Target	Docking area	Grid center x, y, z	Grid size x, y, z	Grid spacing
ESR1	Ligand-binding-domain-associated pocket	8.991, 46.765, 17.761	40, 40, 40 Å	1.0 Å
MTOR	FRB-domain-associated pocket	20.58, 3.821, 33.896	40, 40, 40 Å	1.0 Å
PIK3CA	C2-domain-associated pocket	2.217, -6.635, 0.092	40, 40, 40 Å	1.0 Å

Note: This table summarizes the docking area definitions and grid box parameters used for AutoDock Vina docking. Grid center coordinates, grid size, and grid spacing are reported in Å. The docking areas were defined as domain-associated predicted binding pockets based on the selected receptor structures.
