# Supplementary material for: Dauricine Mitigates Hypoxia Through Targeting ESR1, PIK3CA, and MTOR: A Network Pharmacology and Molecular Dynamics Simulation Investigation
Source: Curr Issues Mol Biol. 2026 May 23;48(6):550. doi: 10.3390/cimb48060550 (PMC13297437; doi:10.3390/cimb48060550)
Supplement: Supplementary file 1 [file cimb-48-00550-s001.zip › cimb-4319076-supplementary/Supplementary File/Supplementary File--Additional Materials for Revision/Table/Supplementary Table S2.pdf]

Supplementary Table S2. Predicted residue-level contacts in the ESR1–dauricine docking complex.

Interacting residue	Chain	Closest protein atom	Closest ligand atom	Minimum heavy-atom distance / Å	Predicted contact type
GLU423	A	OE1	O	2.90	Polar contact / potential hydrogen-bond-related contact
HIS516	A	CE1	O	2.76	Close polar-associated contact
CYS381	B	SG	C	3.20	van der Waals / hydrophobic contact
GLU523	A	OE1	C	3.30	van der Waals / polar-associated contact
LYS520	A	NZ	C	3.40	Close polar-associated contact
TRP383	B	NE1	C	3.48	Hydrophobic / aromatic-associated contact
MET522	B	CB	C	3.48	Hydrophobic / van der Waals contact
TYR526	B	CG	C	3.52	Hydrophobic / aromatic-associated contact
GLU380	B	O	C	3.60	van der Waals / polar-associated contact
ASN519	B	OD1	C	3.68	van der Waals / polar-associated contact
GLU523	B	N	C	3.71	van der Waals contact
LEU525	B	CB	C	3.74	Hydrophobic / van der Waals contact
SER456	B	O	C	3.76	van der Waals / polar-associated contact
ARG515	B	NE	C	3.77	Close polar-associated contact

Note: This table summarizes the predicted residue-level contacts between dauricine and ESR1 in the best-scoring docking pose. The binding pocket corresponds to the ESR1 ligand-binding-domain-associated pocket. Distances represent the minimum heavy-atom distances between dauricine and the corresponding ESR1 residues. Contact types were assigned based on spatial proximity and atom types and should be interpreted as predicted non-covalent contacts. The distance labels displayed in Figure 6 represent visualized interaction distances, whereas the distances listed in this table represent calculated minimum heavy-atom distances; therefore, the two values may not be numerically identical.
