# Supplementary material for: Dauricine Mitigates Hypoxia Through Targeting ESR1, PIK3CA, and MTOR: A Network Pharmacology and Molecular Dynamics Simulation Investigation
Source: Curr Issues Mol Biol. 2026 May 23;48(6):550. doi: 10.3390/cimb48060550 (PMC13297437; doi:10.3390/cimb48060550)
Supplement: Supplementary file 1 [file cimb-48-00550-s001.zip › cimb-4319076-supplementary/Supplementary File/Supplementary File--Additional Materials for Revision/Table/Supplementary Table S3.pdf]

Supplementary Table S3. Predicted residue-level contacts in the MTOR–dauricine docking complex.

Interacting residue	Chain	Closest protein atom	Closest ligand atom	Minimum heavy-atom distance /Å	Predicted contact type
SER2036	B	OG	O	2.91	Polar contact / potential hydrogen-bond-related contact
TRP2102	B	CZ3	O	2.98	Aromatic-associated / van der Waals contact
GLU2033	B	OE1	C	3.35	van der Waals / polar-associated contact
PHE2109	B	CZ	C	3.37	Hydrophobic / aromatic-associated contact
ARG2037	B	N	O	3.41	Polar contact / potential hydrogen-bond-related contact
TYR2106	B	CB	O	3.43	van der Waals / aromatic-associated contact
PHE2040	B	CD2	C	3.46	Hydrophobic / aromatic-associated contact
GLY2041	B	CA	O	3.48	van der Waals contact
LEU2032	B	O	C	3.89	van der Waals / hydrophobic contact

Note: This table summarizes the predicted residue-level contacts between dauricine and MTOR in the best-scoring docking pose. The binding pocket corresponds to the MTOR FRB-domain-associated pocket. Distances represent the minimum heavy-atom distances between dauricine and the corresponding MTOR residues. Contact types were assigned based on spatial proximity and atom types and should be interpreted as predicted non-covalent contacts. The distance labels displayed in Figure 6 represent visualized interaction distances, whereas the distances listed in this table represent calculated minimum heavy-atom distances; therefore, the two values may not be numerically identical.
