# Supplementary material for: Dauricine Mitigates Hypoxia Through Targeting ESR1, PIK3CA, and MTOR: A Network Pharmacology and Molecular Dynamics Simulation Investigation
Source: Curr Issues Mol Biol. 2026 May 23;48(6):550. doi: 10.3390/cimb48060550 (PMC13297437; doi:10.3390/cimb48060550)
Supplement: Supplementary file 1 [file cimb-48-00550-s001.zip › cimb-4319076-supplementary/Supplementary File/Supplementary File--Additional Materials for Revision/Table/Supplementary Table S4.pdf]

Supplementary Table S4. Predicted residue-level contacts in the PIK3CA–dauricine docking complex.

Interacting residue	Chain	Closest protein atom	Closest ligand atom	Minimum heavy-atom distance / Å	Predicted contact type
TYR20	A	OH	O	3.10	Polar contact / potential hydrogen-bond-related contact
ASN22	A	ND2	O	3.25	Polar contact / potential hydrogen-bond-related contact
ALA18	A	O	N	3.36	Polar-associated contact
VAL21	A	O	C	3.43	van der Waals / polar-associated contact
PRO148	A	CD	C	3.47	Hydrophobic / van der Waals contact
THR19	A	OG1	C	3.47	van der Waals / polar-associated contact
GLU146	A	CB	C	3.51	van der Waals / hydrophobic contact
PRO58	A	CD	C	3.53	Hydrophobic / van der Waals contact
VAL23	A	CG2	O	3.56	van der Waals / hydrophobic-associated contact
CYS55	A	O	C	3.73	van der Waals / polar-associated contact
THR147	A	N	C	3.79	van der Waals / polar-associated contact

Note: This table summarizes the predicted residue-level contacts between dauricine and PIK3CA in the best-scoring docking pose. The binding pocket corresponds to the PIK3CA C2-domain-associated pocket. Distances represent the minimum heavy-atom distances between dauricine and the corresponding PIK3CA residues. Contact types were assigned based on spatial proximity and atom types and should be interpreted as predicted non-covalent contacts. The distance labels displayed in Figure 6 represent visualized interaction distances, whereas the distances listed in this table represent calculated minimum heavy-atom distances; therefore, the two values may not be numerically identical.
