# Supplementary material for: Dauricine Mitigates Hypoxia Through Targeting ESR1, PIK3CA, and MTOR: A Network Pharmacology and Molecular Dynamics Simulation Investigation
Source: Curr Issues Mol Biol. 2026 May 23;48(6):550. doi: 10.3390/cimb48060550 (PMC13297437; doi:10.3390/cimb48060550)
Supplement: Supplementary file 1 [file cimb-48-00550-s001.zip › cimb-4319076-supplementary/Supplementary File/Supplementary File-Initial Submission/Molecular Docking/Document preparation-ESR1/vina_license.rtf]

AUTODOCK VINA SOFTWARE TRANSFER LICENSE AGREEMENT

THIS SOFTWARE LICENSE AGREEMENT ("Agreement") is made by and between The Scripps Research Institute ("TSRI"), having an address at 10550 N. Torrey Pines Road, La Jolla, CA 92037 and yourself ("Recipient")

RECITALS
A. TSRI is the owner of the Software (as defined below).
B. TSRI desires to grant to Recipient and Recipient desires to obtain from TSRI a non-exclusive license to use the Software solely in accordance with the terms and on the conditions set forth in this Agreement.

NOW, THEREFORE, the parties hereto agree as follows:
1. DEFINITIONS.
1.1 "Software" shall mean the Vina computer program in binary executable form. 

2. GRANT OF RIGHTS.
The License granted for Software under this Agreement authorizes Recipient on a nonexclusive basis to use one copy of the Software. Recipient may retain one additional copy of the Software for archival purposes. Recipient agrees to use the Software for internal non-commercial research purposes only, and shall not distribute or transfer the Software to anyone not under the Recipient Scientist's direct supervision or beyond the Recipient Scientist's laboratory.

3. DELIVERY.
3.1 Software. TSRI shall deliver to Recipient a master copy of the Software licensed hereunder in binary executable form, suitable for reproduction, in electronic files only.

4. MODIFICATIONS.
4.1 Other Modifications. Recipient may, from time to time, request that TSRI incorporate certain features, enhancements or modifications into the Software. TSRI may, in its sole discretion, undertake to incorporate such changes, which shall be the sole property of TSRI, and distribute the Software so modified to all or any of TSRI's licensees. Any modifications or derivative works based on the Software are considered part of the Software and ownership thereof is retained by TSRI.

5. COPIES AND RECORDS. Recipient agrees to maintain appropriate records of the number and location of all copies of the Software.

6. PROTECTION OF LICENSED SOFTWARE.
6.1 Proprietary Notices. Recipient agrees to respect and not to remove, obliterate, or cancel from view any copyright, trademark, confidentiality or other proprietary notice, mark, or legend appearing on any of the Software or output generated by the Software, and to reproduce and include same on each copy of the Software. 

6.2 Protection of Ownership. Recipient agrees to use its best efforts, consistent with the practices and procedures Recipient takes to protect Recipient's own most valuable proprietary information and materials, and will take all reasonable steps to protect the Software and any pertinent documentation and associated trade secrets against any unauthorized use, reproduction, disclosure or distribution.

7. CONFIDENTIALITY.
7.1 Acknowledgement. Recipient hereby acknowledges and agrees that the Software constitutes and contains valuable proprietary products and trade secrets of TSRI embodying substantial creative efforts and confidential information, ideas, and expressions. Accordingly, Recipient agrees to treat (and take precautions to ensure that its employees treat) the Software as confidential, not to disclose or permit to any third party or entity access to the Software or any portion thereof without the written permission of a duly authorized representative of TSRI.

7.2 Injunctive Relief. Recipient acknowledges that the unauthorized use, transfer or disclosure of the Software or copies thereof will (i) substantially diminish the value to TSRI of the trade secrets and other proprietary interests that are the subject of this Agreement; (ii) render TSRI's remedy at law for such unauthorized use, disclosure or transfer inadequate; and (iii) cause irreparable injury in a short period of time. If Recipient breaches any of its obligations with respect to the use or confidentiality of the
Software, TSRI shall be entitled to seek equitable relief to protect its interests therein, including, but not limited to, preliminary and permanent injunctive relief.

7.3 Survival. Recipient's obligations under this Article 7 will survive the termination of this Agreement or of any license granted under this Agreement for whatever reason.

8. WARRANTIES; SUPERIOR RIGHTS.
8.1 Government Rights. Recipient understands that the Software may have been developed under a funding agreement with the Government of the United States of America and, if so, that the Government may have certain rights relative thereto. This Agreement is explicitly made subject to the Government's rights under any such agreement and any applicable law or regulation, if any. To the extent that there
is a conflict between any such agreement, applicable law or regulation and this Agreement, the terms of such Government agreement, applicable law or regulation shall prevail.

8.2 Disclaimer of Warranties. ANY INFORMATION, MATERIALS OR SERVICES FURNISHED BY TSRI PURSUANT TO THIS AGREEMENT ARE ON AN "AS IS" BASIS. TSRI DOES NOT REPRESENT OR WARRANT THAT ALL ERRORS IN THE SOFTWARE WILL BE CORRECTED. THERE ARE NO WARRANTIES RESPECTING THE SOFTWARE OR SERVICES PROVIDED HEREUNDER, EITHER EXPRESS OR IMPLIED, INCLUDING BUT NOT LIMITED TO ANY WARRANTY OF DESIGN, MERCHANTABILITY, FITNESS FOR A PARTICULAR PURPOSE, EVEN IF TSRI HAS BEEN INFORMED OF SUCH PURPOSE OR THAT THE SOFTWARE WILL NOT INFRINGE ANY PATENT, COPYRIGHT, TRADEMARK, OR OTHER PROPRIETARY RIGHTS. NO AGENT OF TSRI IS AUTHORIZED TO ALTER OR EXCEED THE WARRANTY OBLIGATIONS OF TSRI AS SET FORTH HEREIN.

9. INDEMNIFICATION.
Except to the extent prohibited by law, Recipient shall indemnify and hold harmless, TSRI, its trustees, officers, agents and employees from and against any claims, demands, or causes of action whatsoever, including without limitation those arising on account of Recipient's modification or enhancement of the Software or otherwise caused by, or arising out of, or resulting from, the exercise or practice of the license granted hereunder by Recipient, its sublicensees, if any, its subsidiaries or their officers, employees, agents or representatives.

10. NONASSIGNABILITY. Any and all assignments of this Agreement or any rights granted hereunder by Recipient without the prior written consent of TSRI are void except (i) to an affiliate of Recipient or (ii) as expressly permitted hereunder.

11. GOVERNING LAW; JURISDICTION AND VENUE. The validity, interpretation, construction and performance of this Agreement shall be governed by the laws of the State of California.

12. SEVERABILITY. If any provision of this Agreement shall be held by a court of competent jurisdiction to be illegal, invalid or unenforceable, the remaining provisions shall remain in full force and effect. This Agreement and its exhibits contain the entire understanding and agreement between the parties respecting the subject matter hereof. This Agreement may not be supplemented, modified, amended, released or discharged except by an instrument in writing signed by each party's duly authorized representative. All captions and headings in this Agreement are for purposes of convenience only and
shall not affect the construction or interpretation of any of its provisions. Any waiver by either party of any default or breach hereunder shall not constitute a waiver of any provision of this Agreement or of any subsequent default or breach of the same or a different kind.
