# Supplementary material for: Dauricine Mitigates Hypoxia Through Targeting ESR1, PIK3CA, and MTOR: A Network Pharmacology and Molecular Dynamics Simulation Investigation
Source: Curr Issues Mol Biol. 2026 May 23;48(6):550. doi: 10.3390/cimb48060550 (PMC13297437; doi:10.3390/cimb48060550)
Supplement: Supplementary file 1 [file cimb-48-00550-s001.zip › cimb-4319076-supplementary/Supplementary File/Supplementary File-Initial Submission/Toxicology/Drug-likeness and toxicity.pdf]

Compound	MW	HBA	HBD	MLogP	Lipinski's violations	Bioavailability Score	TPSA
Dauricine	624.32	8	1	3.46	1	0.55	72.86

Toxicity Category	Assessment Endpoint	Prediction	Value / Probability
Cardiotoxicity	hERG Inhibition	Inactive	0.899
Carcinogenicity	Carcinogenicity	Inactive	0.59
Acute Toxicity	Oral LD50 (Rat)	Low Toxicity	1365 mg/kg
Mechanism-Based Toxicity	Mitochondrial Toxicity (MMP)	Inactive	0.86
	Oxidative Stress (ARE Pathway)	Inactive	0.98
	Endocrine Disruption (AR, ER, AhR)	Inactive	>0.90
Other Organ Toxicity	Nephrotoxicity	Inactive	0.56
