# Supplementary material for: Dauricine Mitigates Hypoxia Through Targeting ESR1, PIK3CA, and MTOR: A Network Pharmacology and Molecular Dynamics Simulation Investigation
Source: Curr Issues Mol Biol. 2026 May 23;48(6):550. doi: 10.3390/cimb48060550 (PMC13297437; doi:10.3390/cimb48060550)
Supplement: Supplementary file 1 [file cimb-48-00550-s001.zip › cimb-4319076-supplementary/Supplementary File/Supplementary File-Initial Submission/Toxicology/Drug-likeness and toxicity.pptx]

## Slide 1
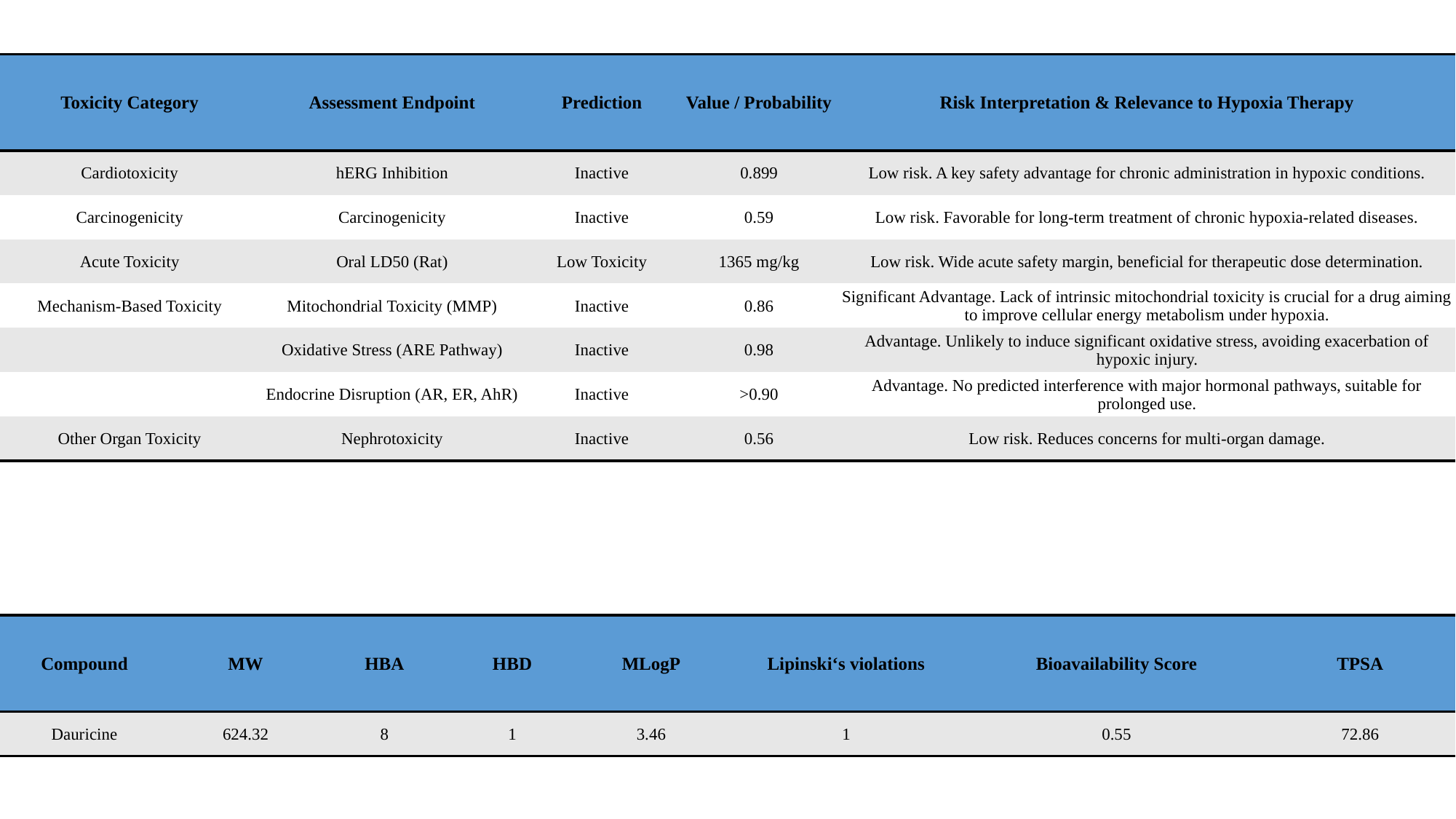

| Toxicity Category | Assessment Endpoint | Prediction | Value / Probability | Risk Interpretation & Relevance to Hypoxia Therapy |
| --- | --- | --- | --- | --- |
| Cardiotoxicity | hERG Inhibition | Inactive | 0.899 | Low risk. A key safety advantage for chronic administration in hypoxic conditions. |
| Carcinogenicity | Carcinogenicity | Inactive | 0.59 | Low risk. Favorable for long-term treatment of chronic hypoxia-related diseases. |
| Acute Toxicity | Oral LD50 (Rat) | Low Toxicity | 1365 mg/kg | Low risk. Wide acute safety margin, beneficial for therapeutic dose determination. |
| Mechanism-Based Toxicity | Mitochondrial Toxicity (MMP) | Inactive | 0.86 | Significant Advantage. Lack of intrinsic mitochondrial toxicity is crucial for a drug aiming to improve cellular energy metabolism under hypoxia. |
| | Oxidative Stress (ARE Pathway) | Inactive | 0.98 | Advantage. Unlikely to induce significant oxidative stress, avoiding exacerbation of hypoxic injury. |
| | Endocrine Disruption (AR, ER, AhR) | Inactive | >0.90 | Advantage. No predicted interference with major hormonal pathways, suitable for prolonged use. |
| Other Organ Toxicity | Nephrotoxicity | Inactive | 0.56 | Low risk. Reduces concerns for multi-organ damage. |
| Compound | MW | HBA | HBD | MLogP | Lipinski‘s violations | Bioavailability Score | TPSA |
| --- | --- | --- | --- | --- | --- | --- | --- |
| Dauricine | 624.32 | 8 | 1 | 3.46 | 1 | 0.55 | 72.86 |
