# Supplementary figures and images for: Dauricine Mitigates Hypoxia Through Targeting ESR1, PIK3CA, and MTOR: A Network Pharmacology and Molecular Dynamics Simulation Investigation
Source: Curr Issues Mol Biol. 2026 May 23;48(6):550. doi: 10.3390/cimb48060550 (PMC13297437; doi:10.3390/cimb48060550)

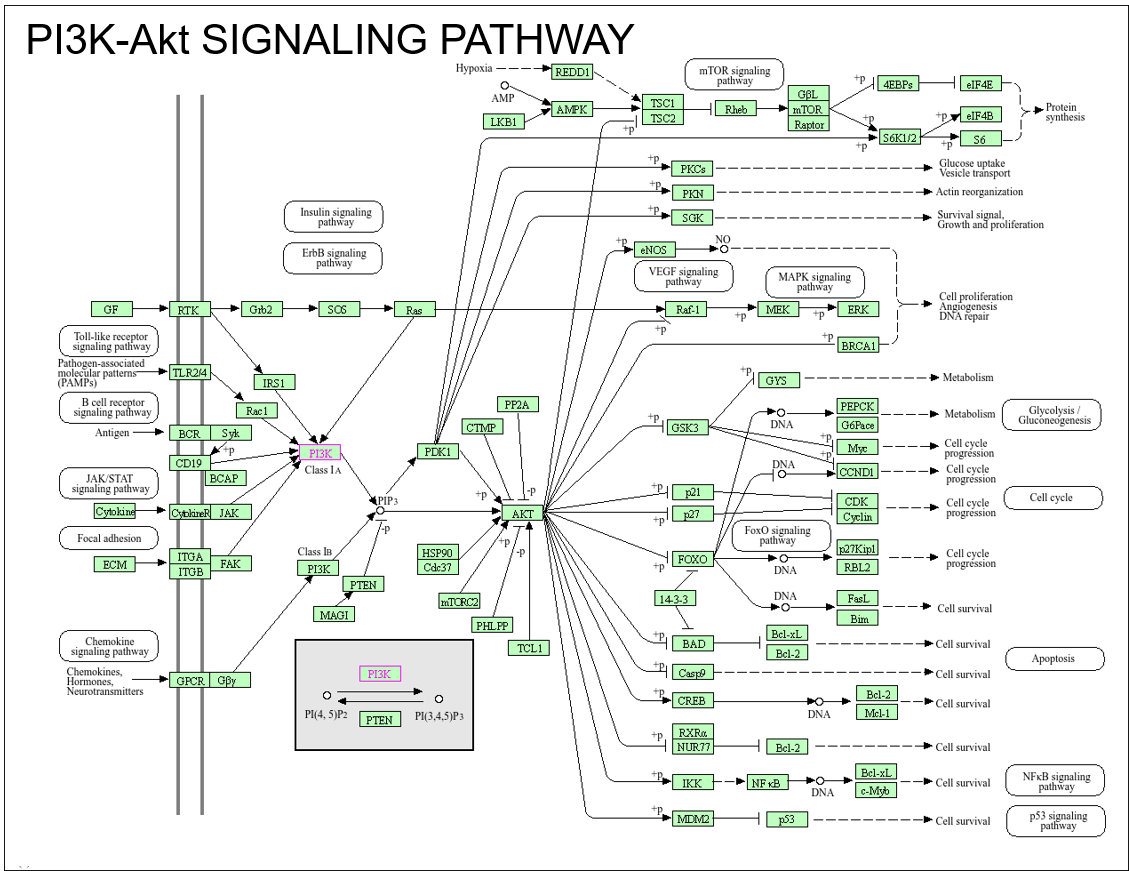

Supplement: Supplementary file 1 [file cimb-48-00550-s001.zip › cimb-4319076-supplementary/Supplementary File/Supplementary File--Additional Materials for Revision/Figure/Supplementary Figure S1. Core signaling pathway for hypoxia adaptation the PI3K-Akt signaling pathway..tif]

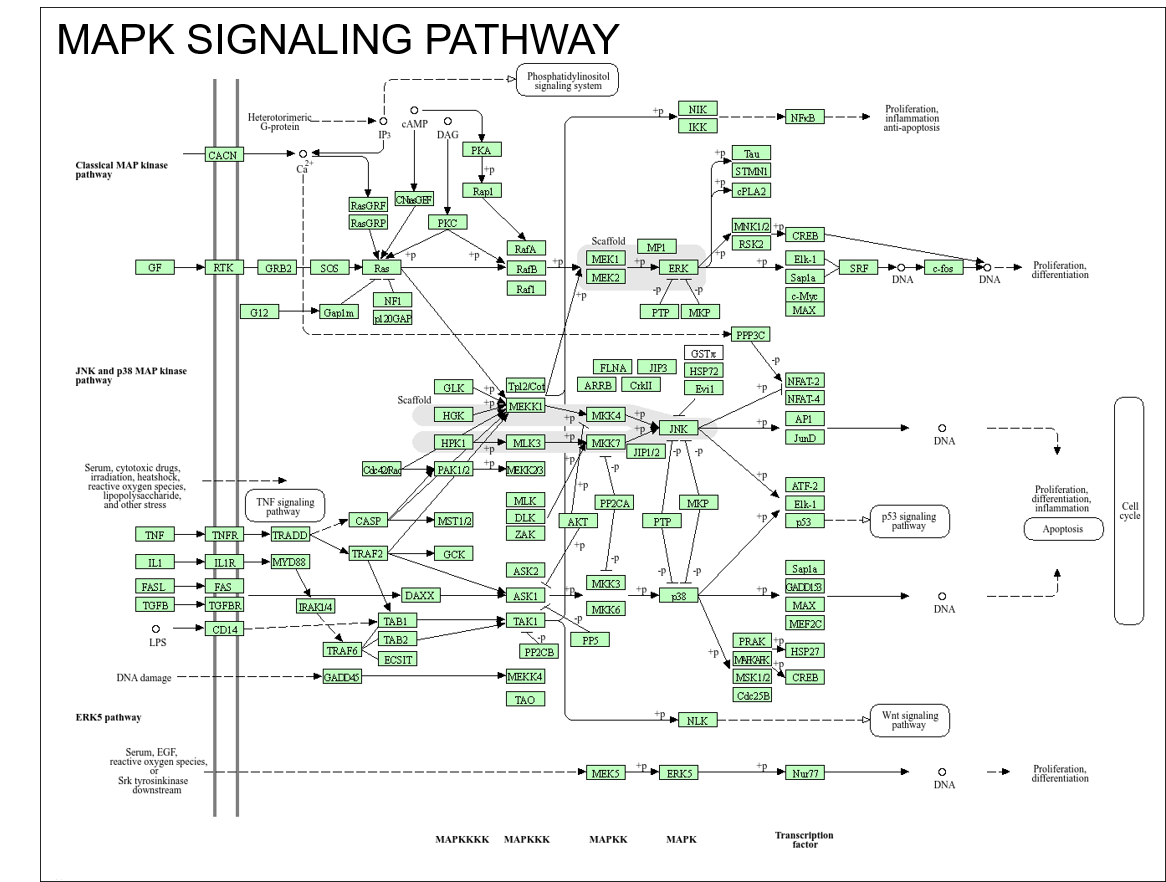

Supplement: Supplementary file 1 [file cimb-48-00550-s001.zip › cimb-4319076-supplementary/Supplementary File/Supplementary File--Additional Materials for Revision/Figure/Supplementary Figure S2. Core signaling pathway for hypoxia adaptation the MAPK signaling pathway..tif]

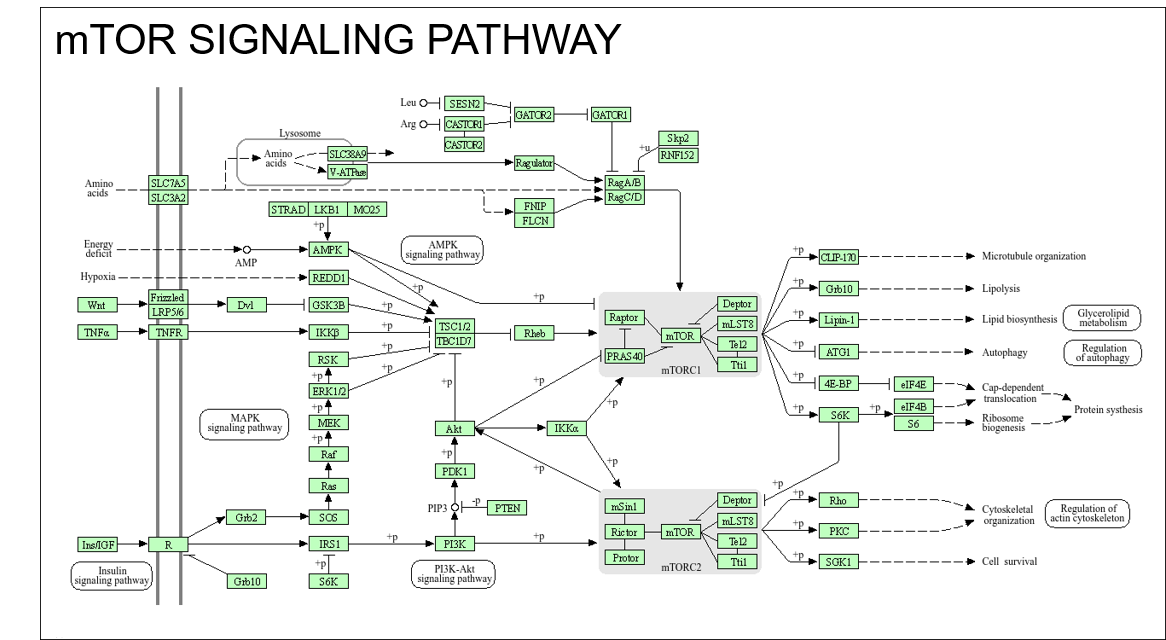

Supplement: Supplementary file 1 [file cimb-48-00550-s001.zip › cimb-4319076-supplementary/Supplementary File/Supplementary File--Additional Materials for Revision/Figure/Supplementary Figure S3. Core signaling pathway for hypoxia adaptation the mTOR signaling pathway..tif]

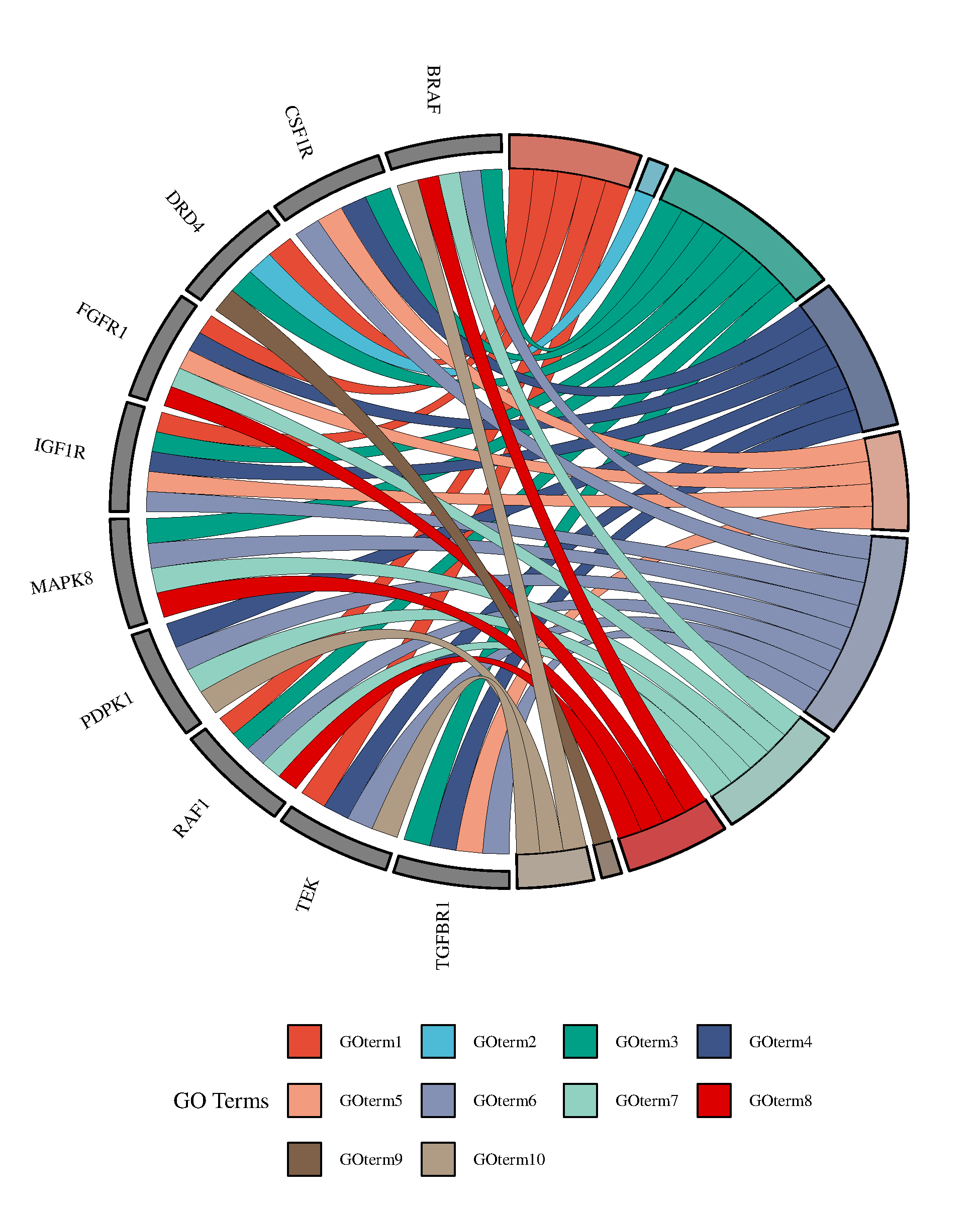

Supplement: Supplementary file 1 [file cimb-48-00550-s001.zip › cimb-4319076-supplementary/Supplementary File/Supplementary File-Initial Submission/Enrichment analysis/GO/Chord/BP/BP-Chord 300dpi.png]

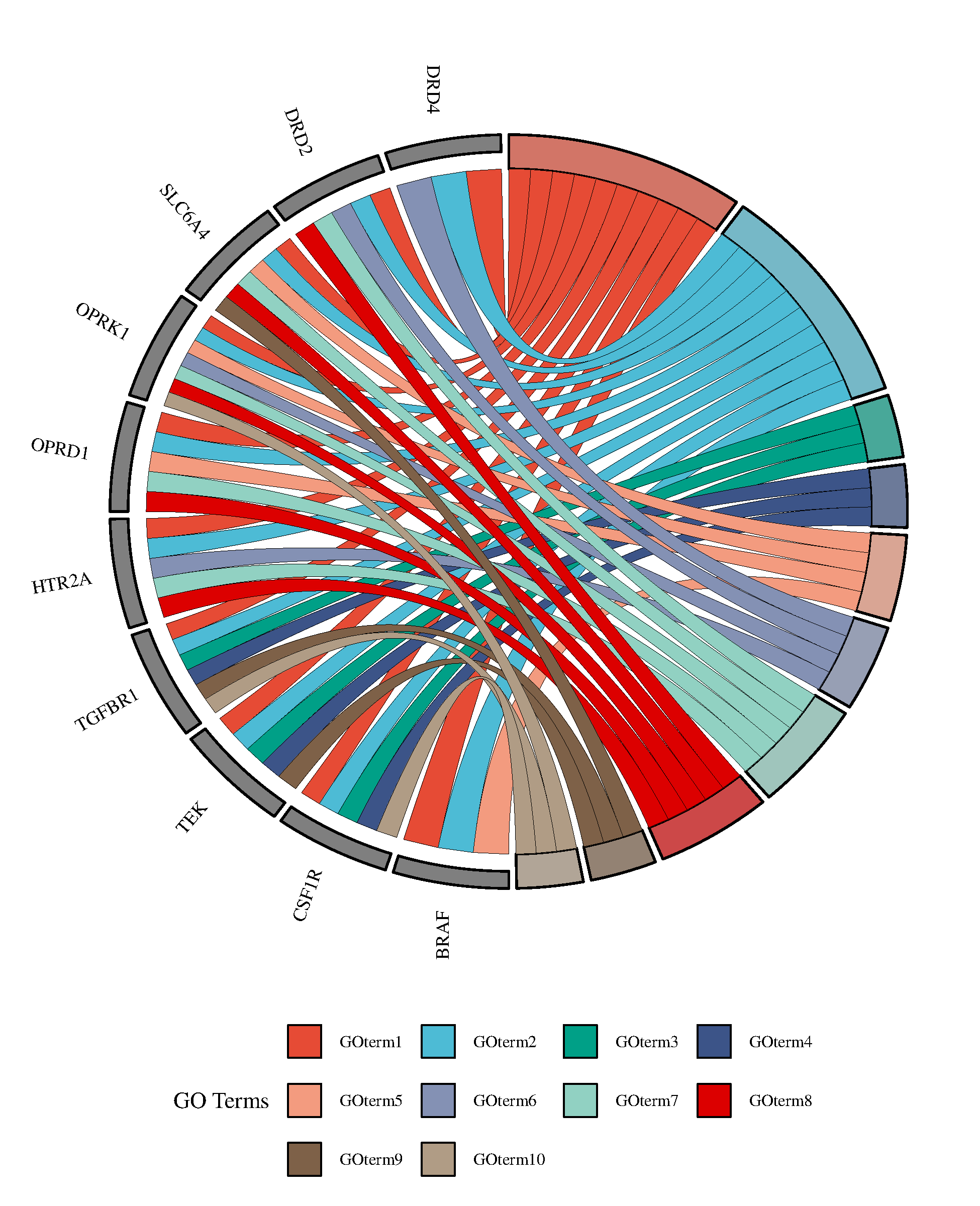

Supplement: Supplementary file 1 [file cimb-48-00550-s001.zip › cimb-4319076-supplementary/Supplementary File/Supplementary File-Initial Submission/Enrichment analysis/GO/Chord/CC/CC-Chord 300dpi.png]

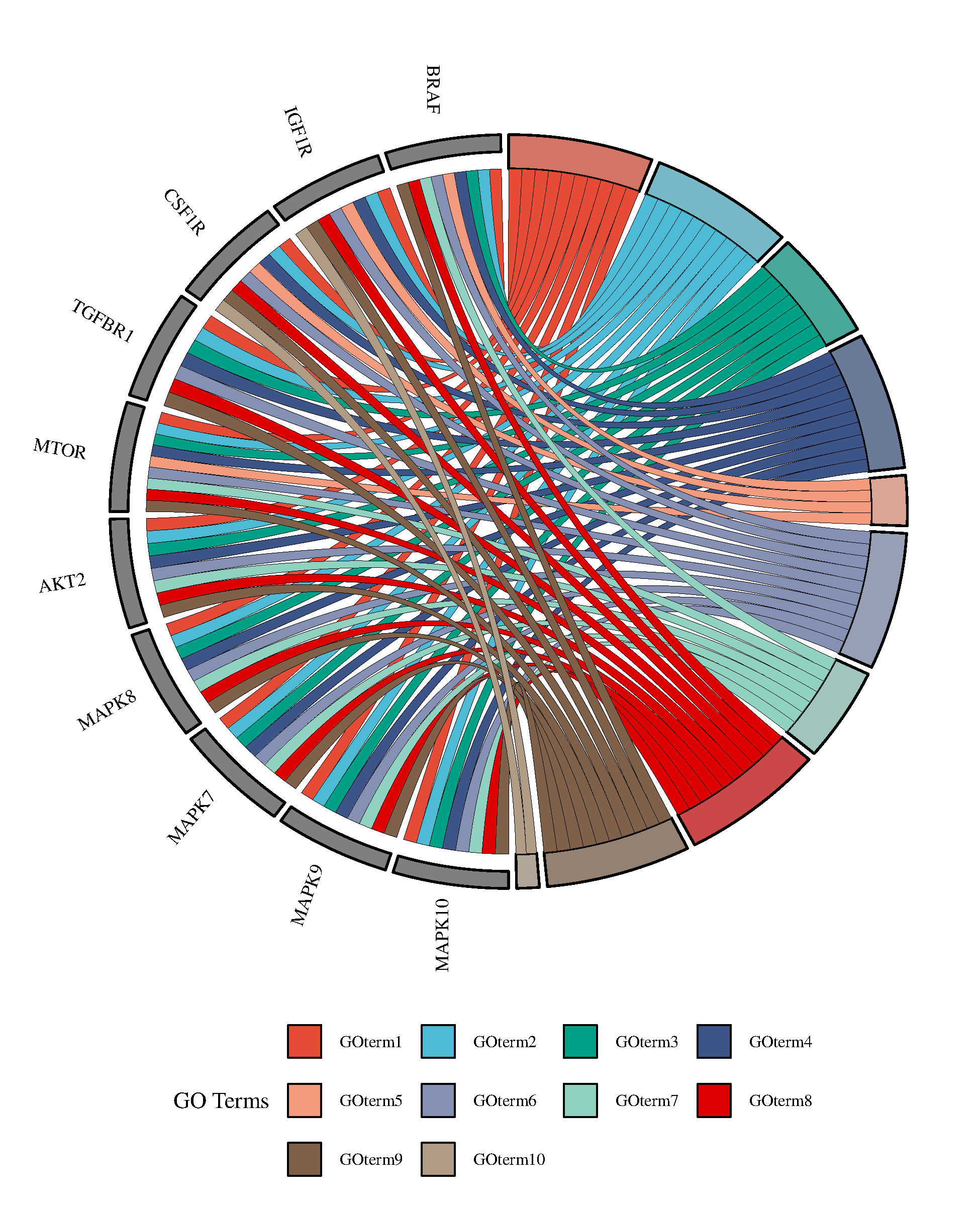

Supplement: Supplementary file 1 [file cimb-48-00550-s001.zip › cimb-4319076-supplementary/Supplementary File/Supplementary File-Initial Submission/Enrichment analysis/GO/Chord/MF/MF-Chord 300 dpi.png]

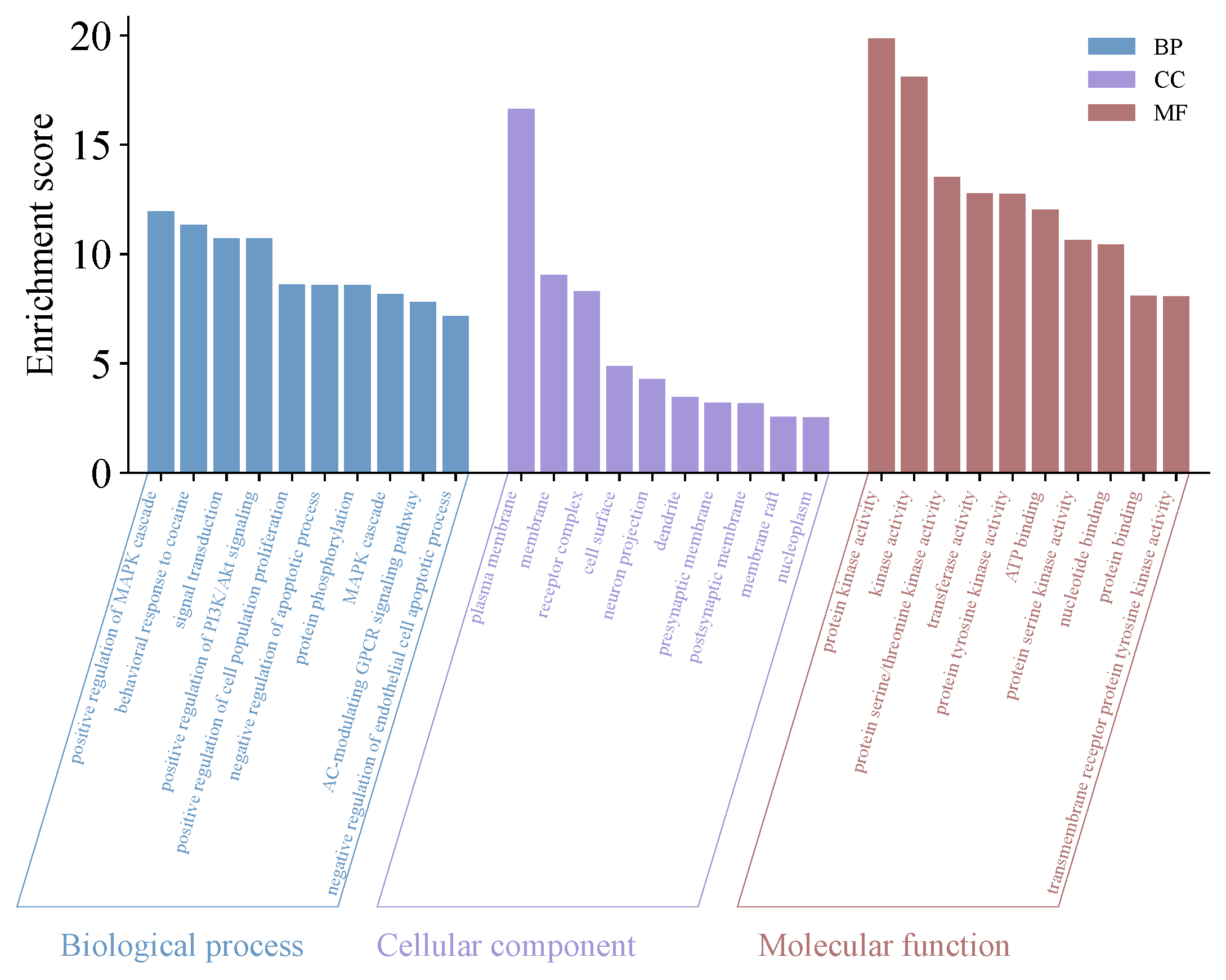

Supplement: Supplementary file 1 [file cimb-48-00550-s001.zip › cimb-4319076-supplementary/Supplementary File/Supplementary File-Initial Submission/Enrichment analysis/GO/Integrated three-part analysis/GO Integrated three-part analysis 300 dpi.png]

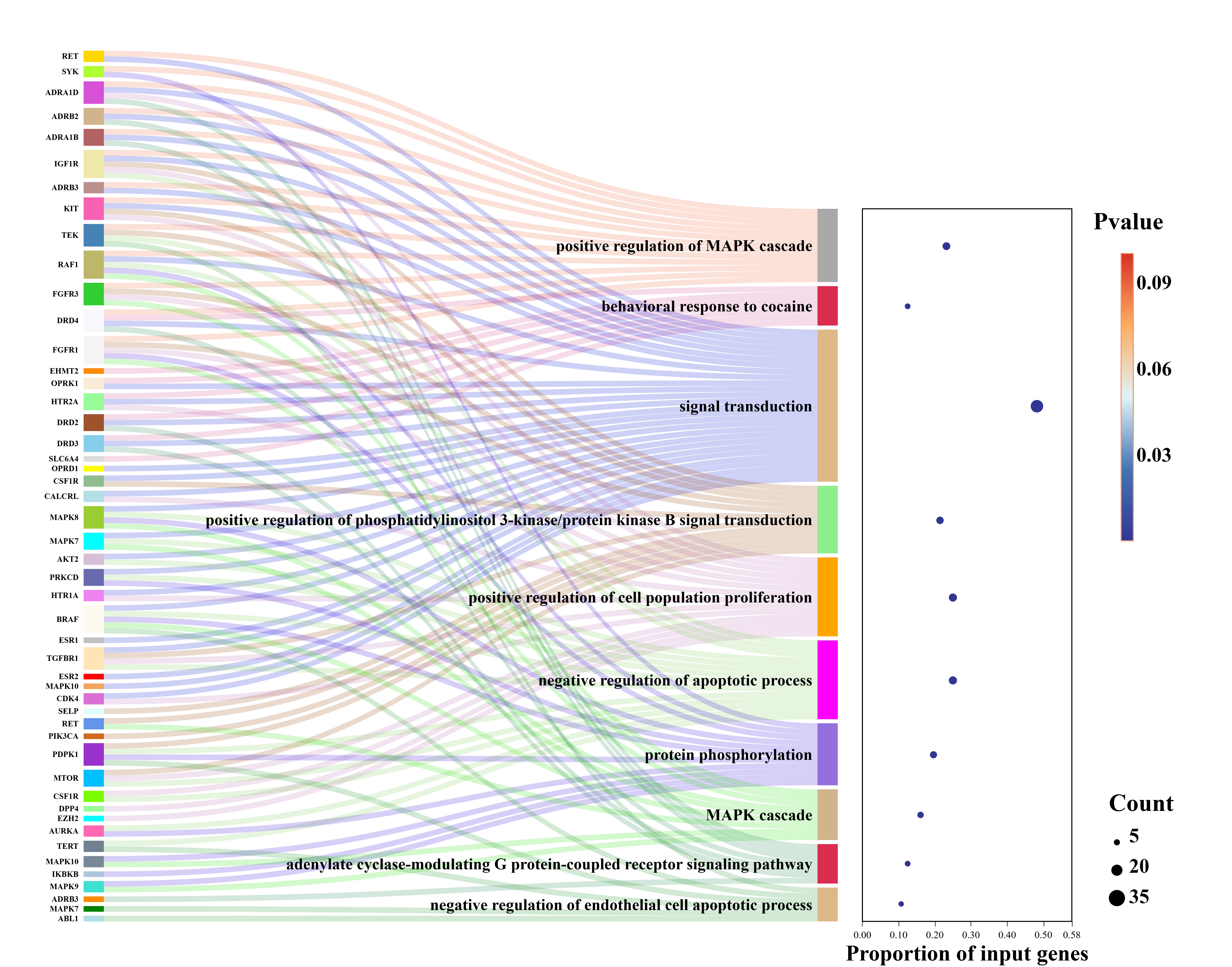

Supplement: Supplementary file 1 [file cimb-48-00550-s001.zip › cimb-4319076-supplementary/Supplementary File/Supplementary File-Initial Submission/Enrichment analysis/Sankey bubble diagram/BP Sankey bubble diagram/BP-900dpi.png]

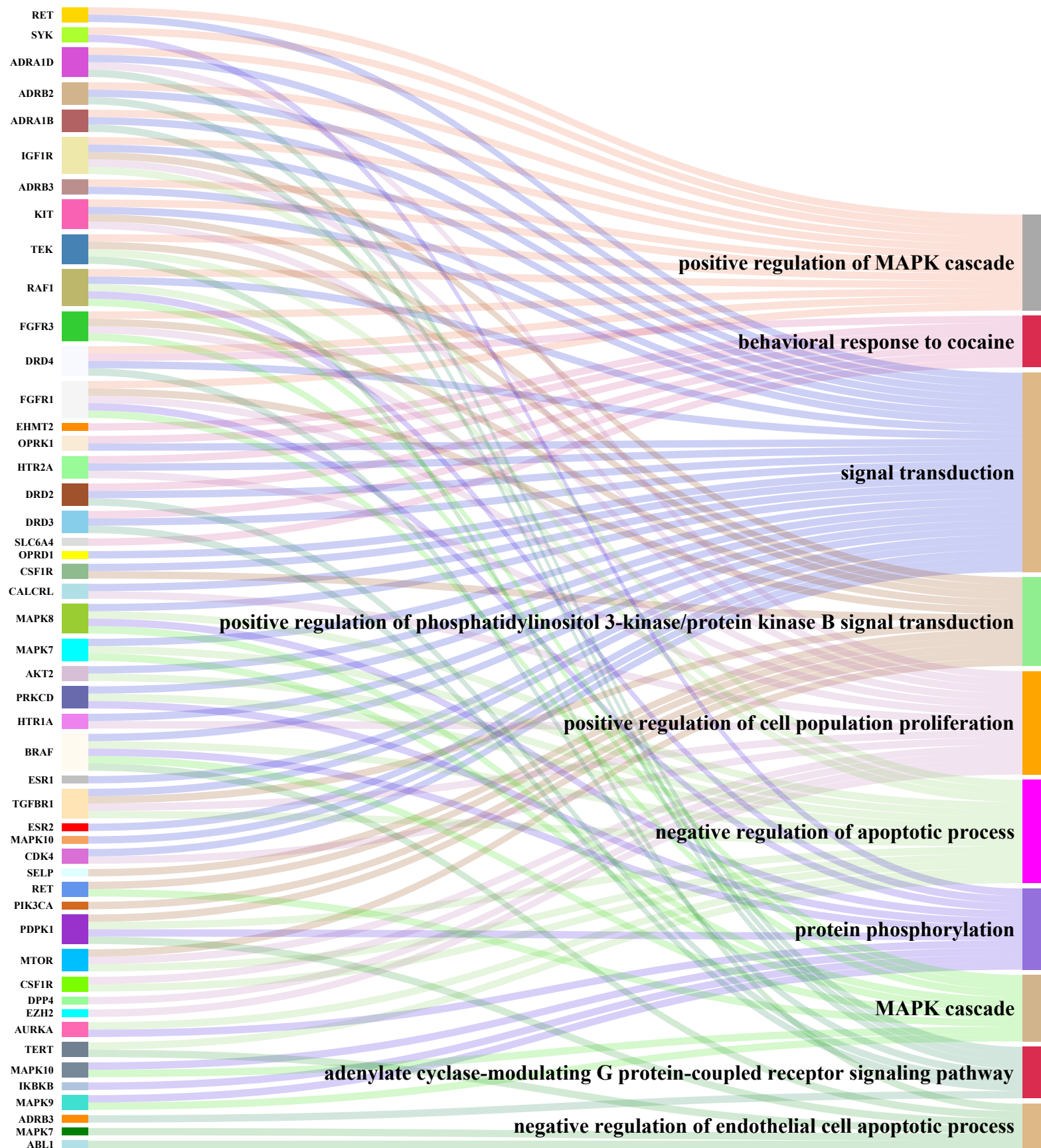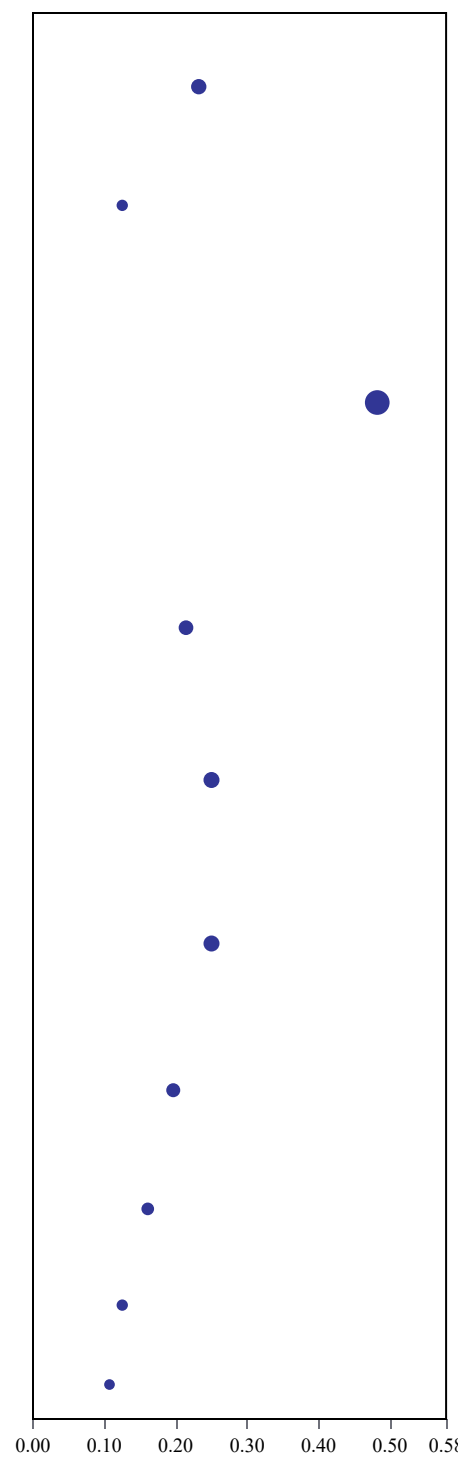

**Pvalue**

0.09

0.06

0.03

**Count**

• 5

• 20

• 35

**Proportion of input genes**

Supplement: Supplementary file 1 [file cimb-48-00550-s001.zip › cimb-4319076-supplementary/Supplementary File/Supplementary File-Initial Submission/Enrichment analysis/Sankey bubble diagram/BP Sankey bubble diagram/BP-PDF.pdf]

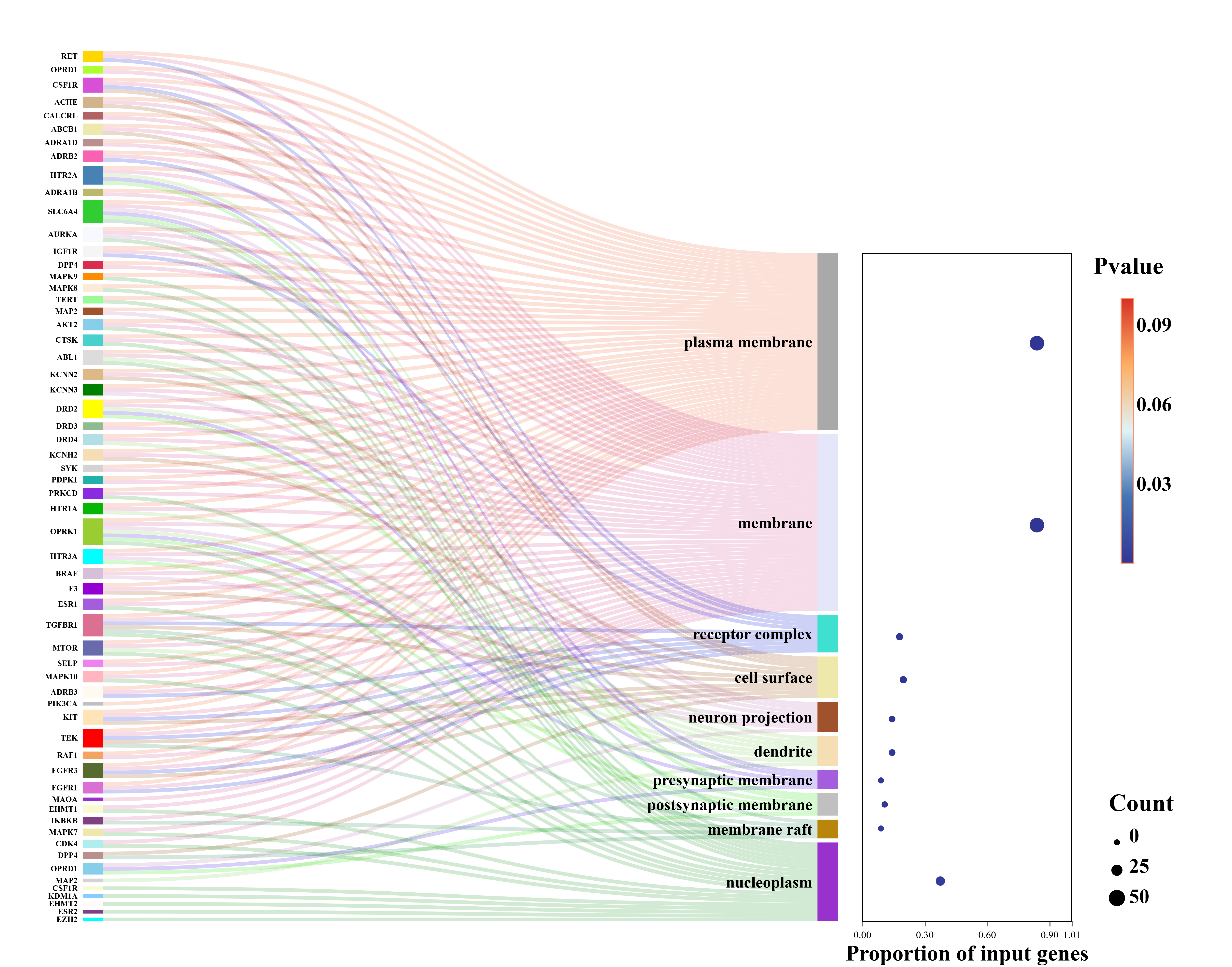

Supplement: Supplementary file 1 [file cimb-48-00550-s001.zip › cimb-4319076-supplementary/Supplementary File/Supplementary File-Initial Submission/Enrichment analysis/Sankey bubble diagram/CC Sankey bubble diagram/CC-900dpi.png]

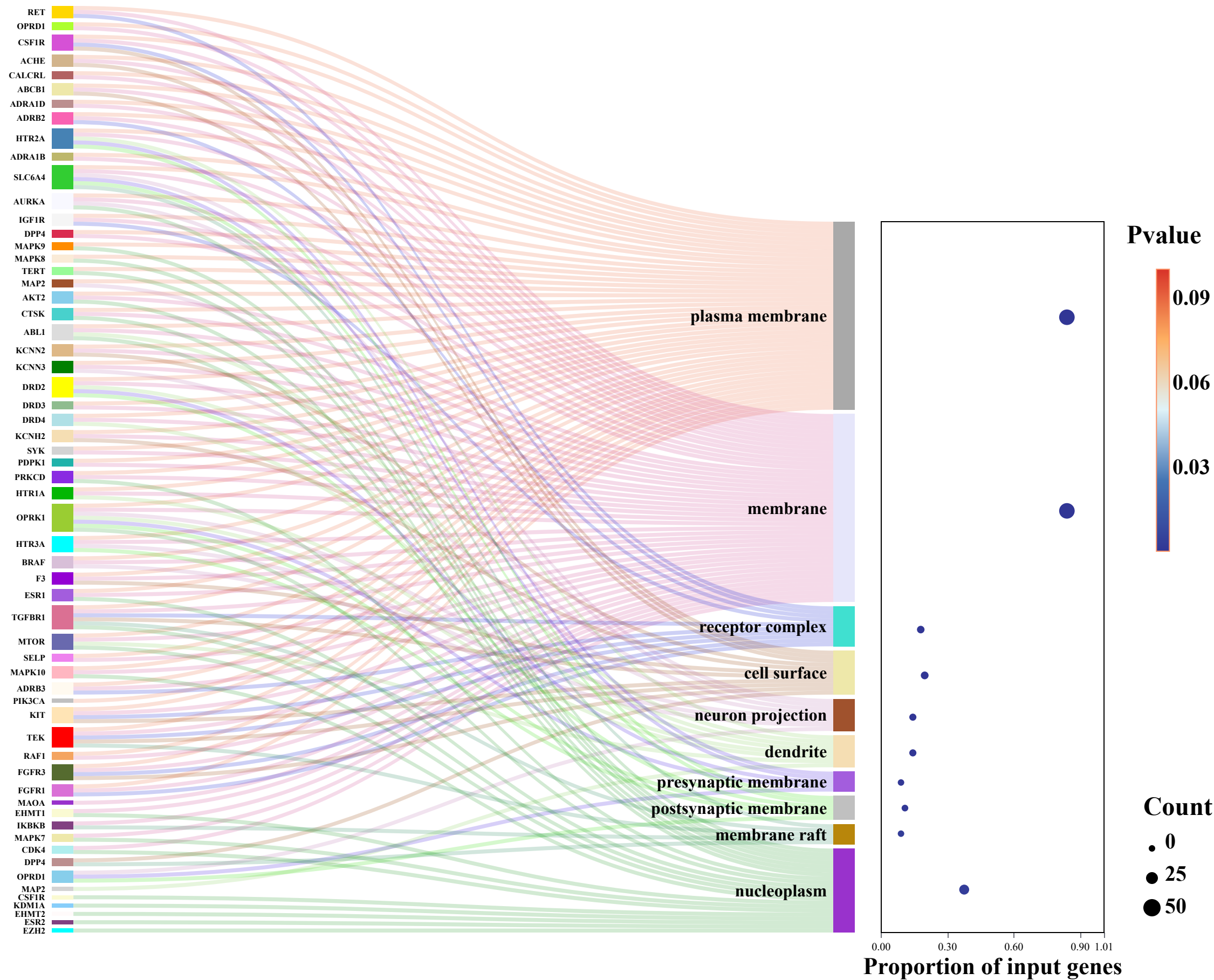

Supplement: Supplementary file 1 [file cimb-48-00550-s001.zip › cimb-4319076-supplementary/Supplementary File/Supplementary File-Initial Submission/Enrichment analysis/Sankey bubble diagram/CC Sankey bubble diagram/CC-PDF.pdf]

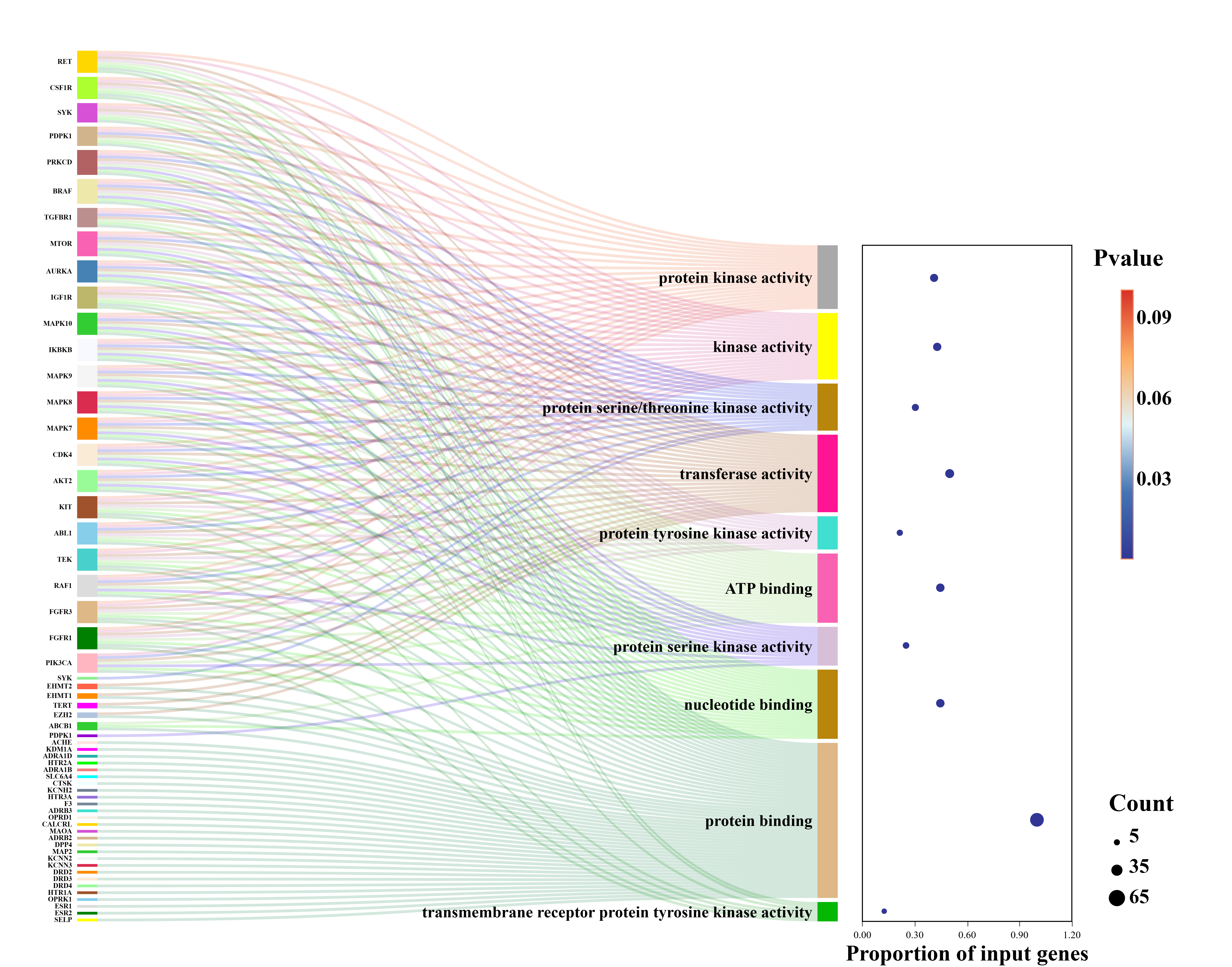

Supplement: Supplementary file 1 [file cimb-48-00550-s001.zip › cimb-4319076-supplementary/Supplementary File/Supplementary File-Initial Submission/Enrichment analysis/Sankey bubble diagram/MF Sankey bubble diagram/MF-900dpi.png]

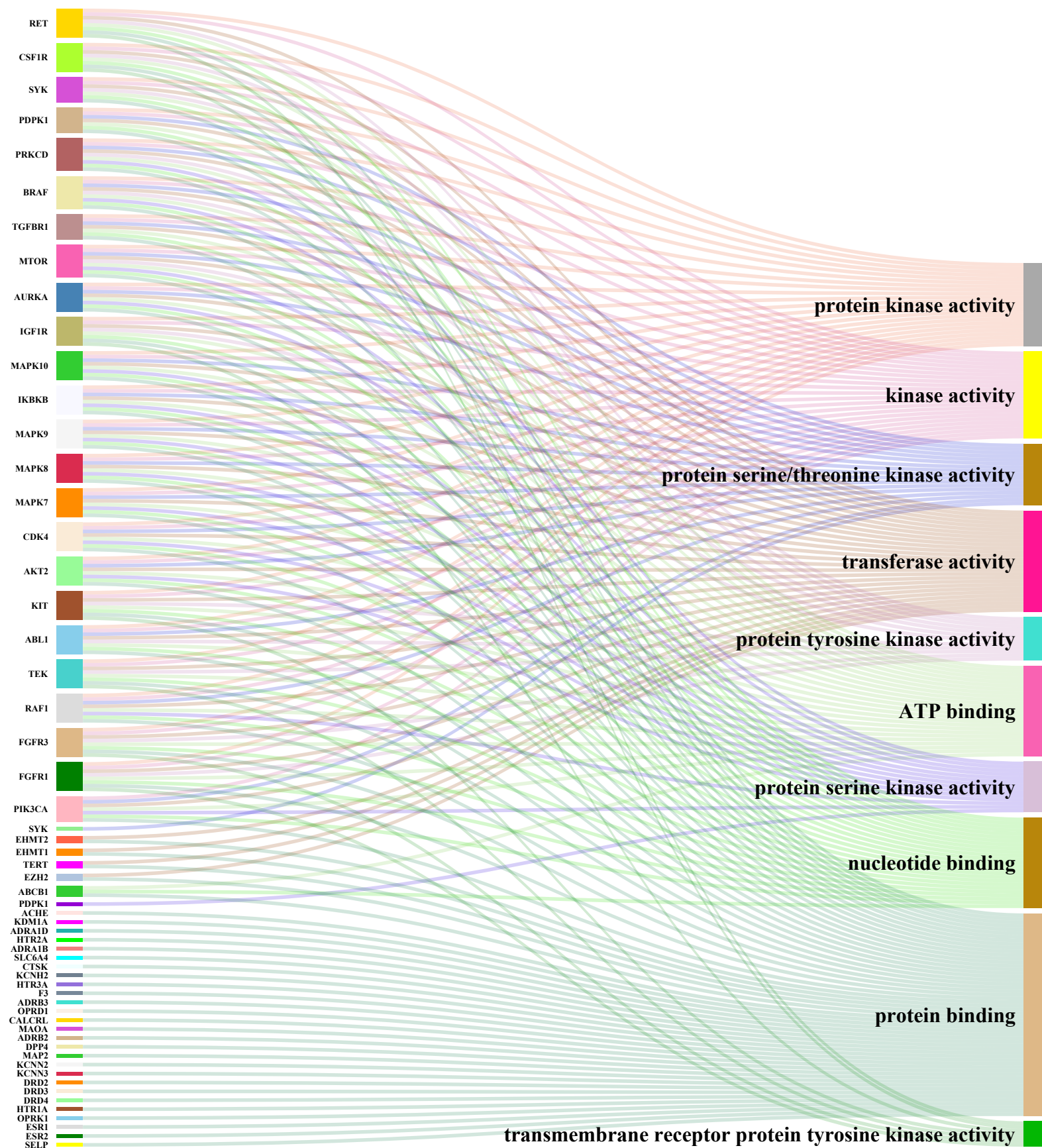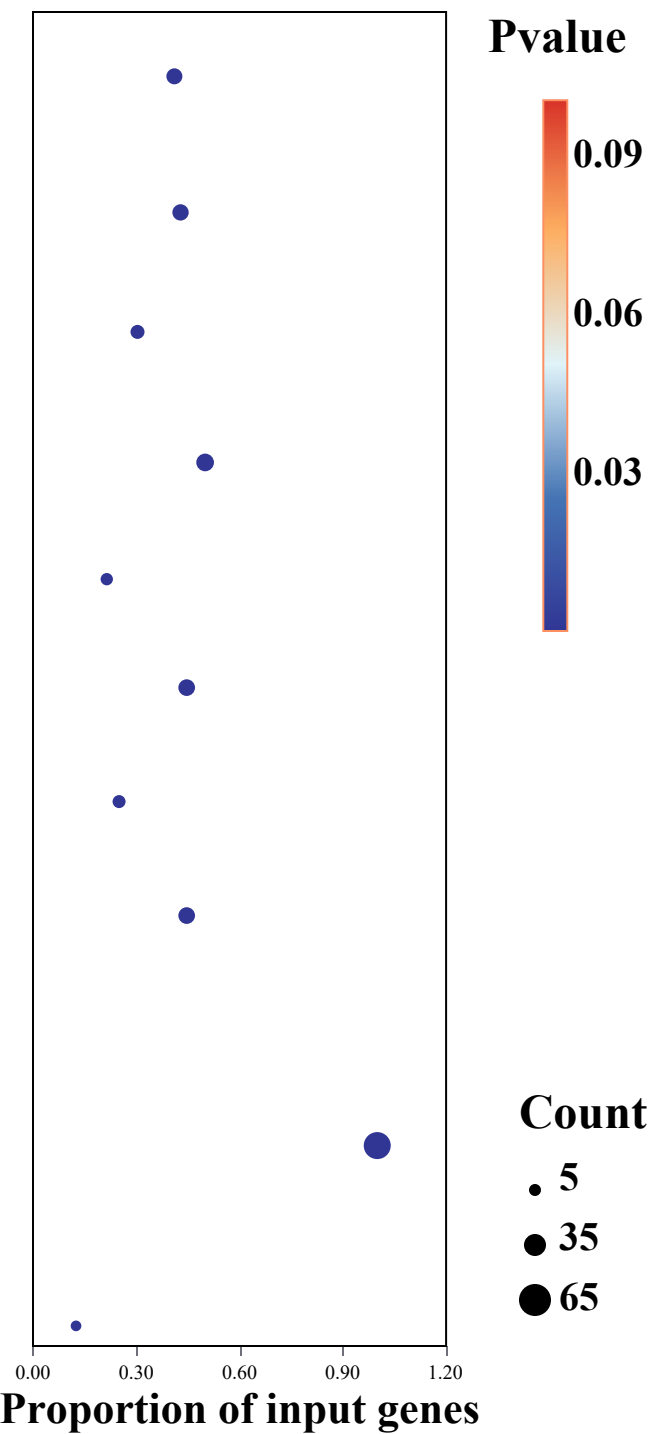

Supplement: Supplementary file 1 [file cimb-48-00550-s001.zip › cimb-4319076-supplementary/Supplementary File/Supplementary File-Initial Submission/Enrichment analysis/Sankey bubble diagram/MF Sankey bubble diagram/MF-PDF.pdf]

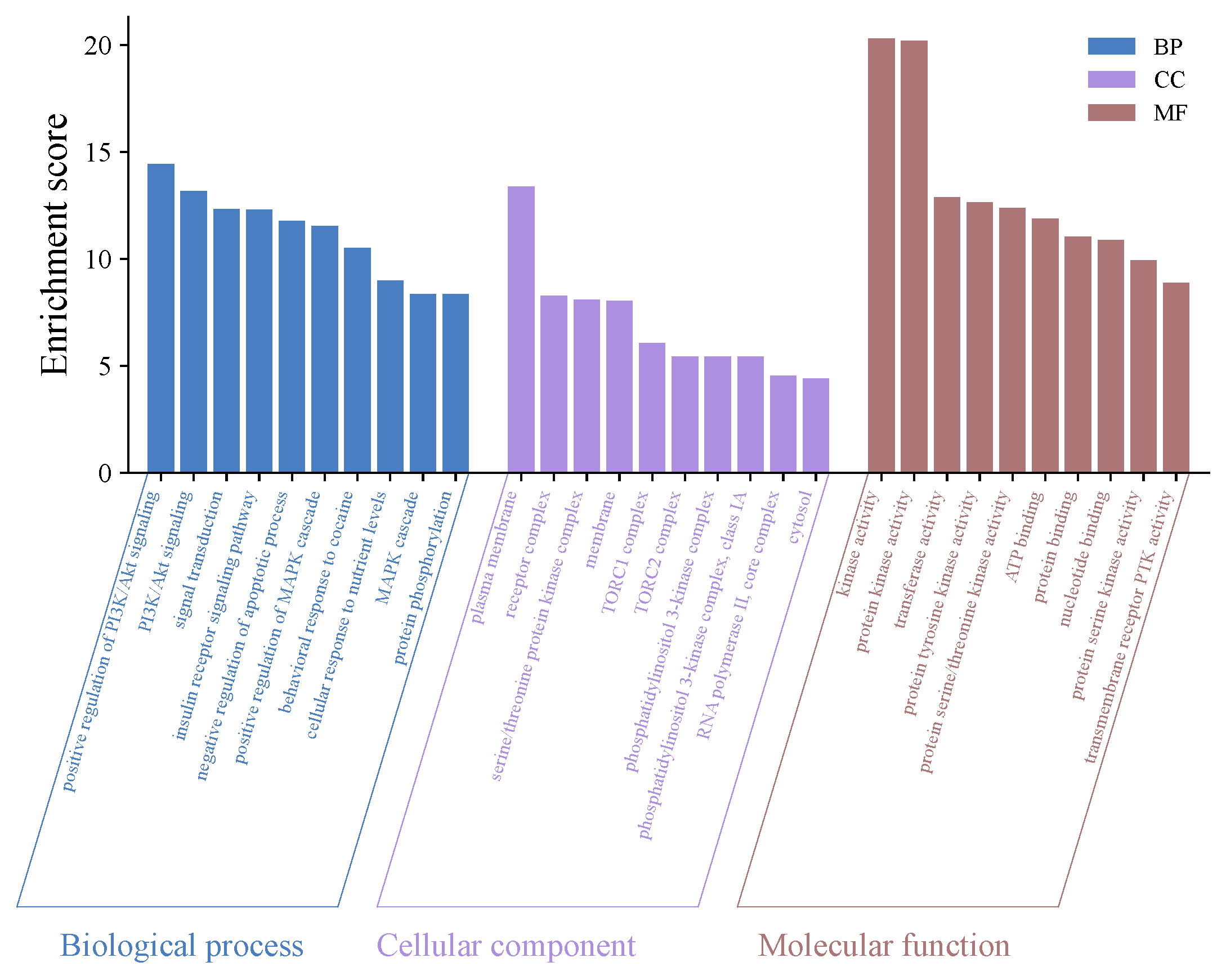

Supplement: Supplementary file 1 [file cimb-48-00550-s001.zip › cimb-4319076-supplementary/Supplementary File/Supplementary File-Initial Submission/GMFA-Expanded genes/Integrated three-part analysis/Integrated three-part analysis-Expanded genes.png]

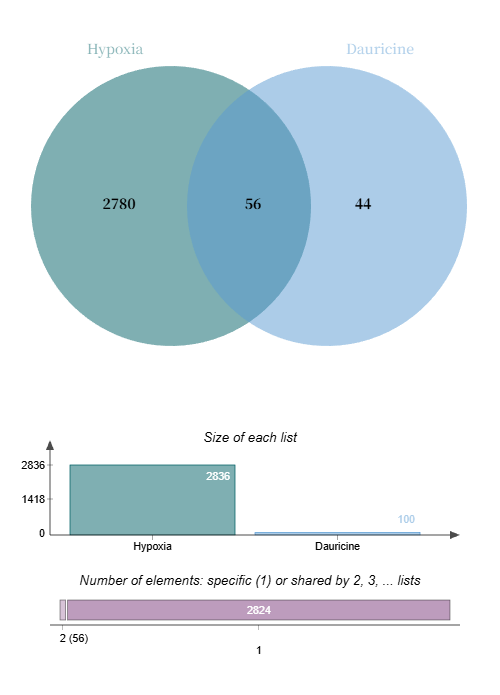

Supplement: Supplementary file 1 [file cimb-48-00550-s001.zip › cimb-4319076-supplementary/Supplementary File/Supplementary File-Initial Submission/Identify the overlapping genes between drug targets and disease genes/Drug-disease Venn.png]

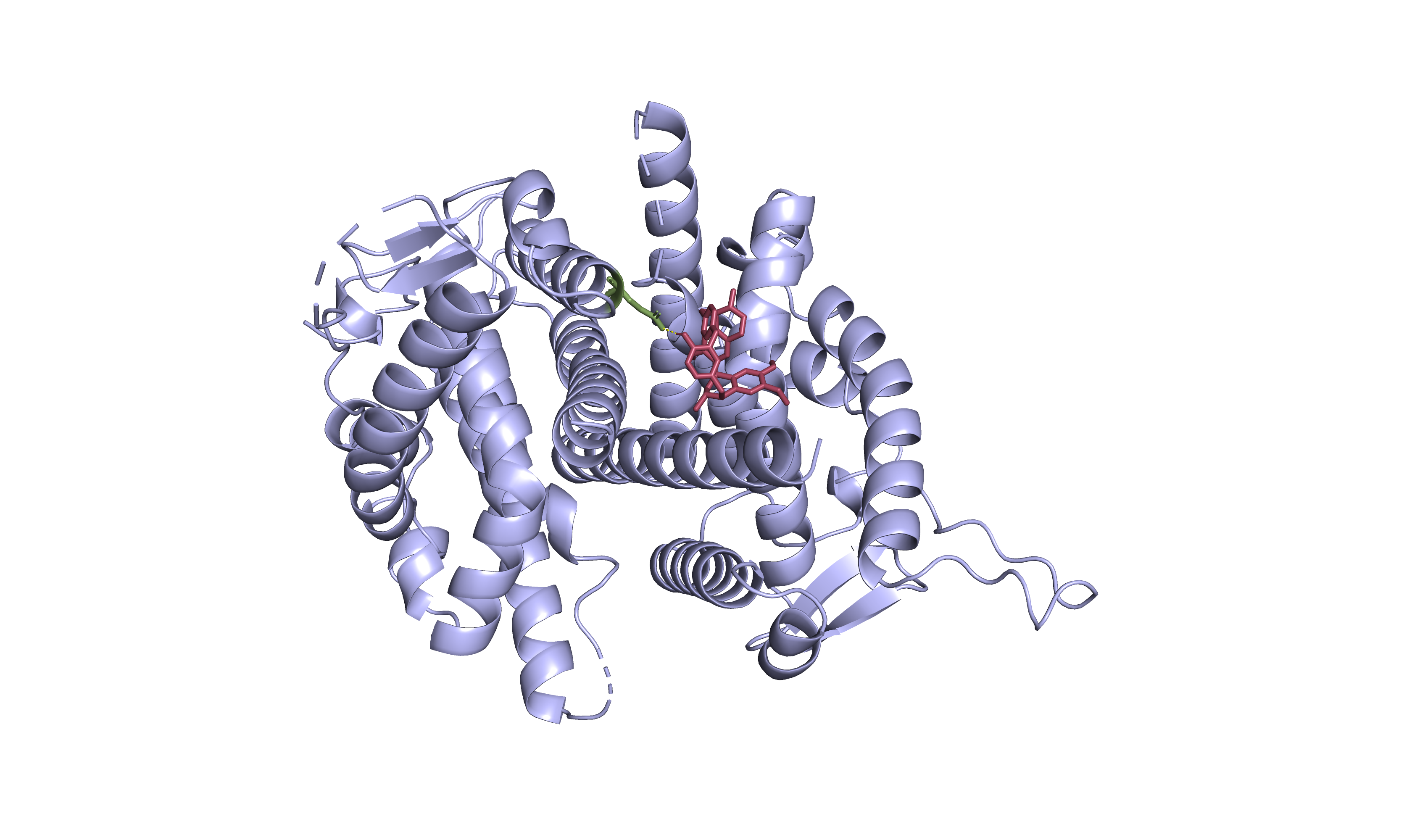

Supplement: Supplementary file 1 [file cimb-48-00550-s001.zip › cimb-4319076-supplementary/Supplementary File/Supplementary File-Initial Submission/Molecular Docking/Document preparation-ESR1/P1-ES.png]

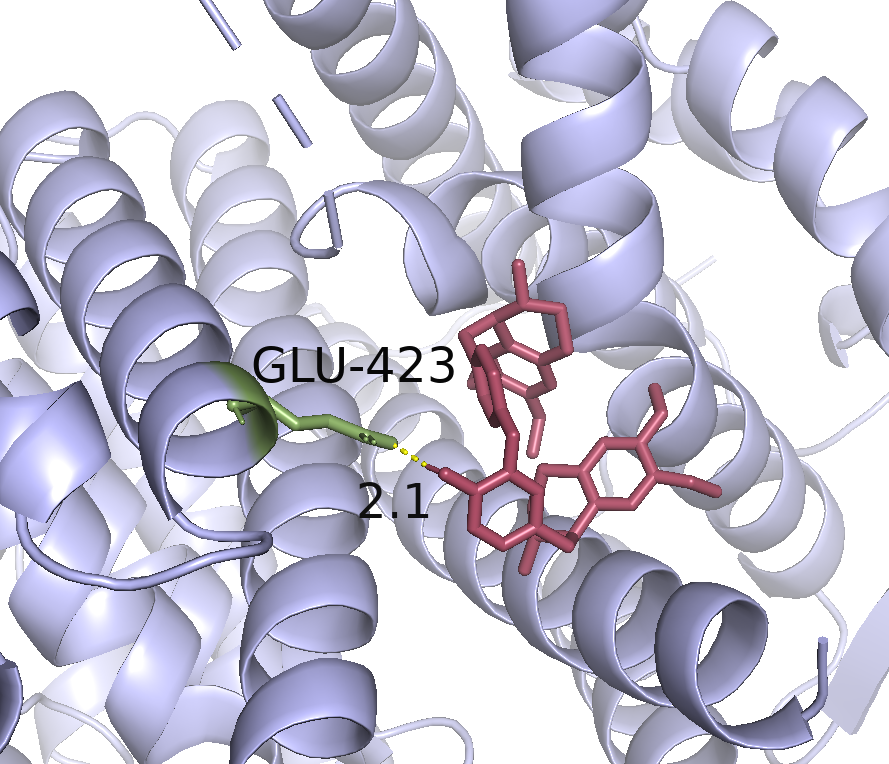

Supplement: Supplementary file 1 [file cimb-48-00550-s001.zip › cimb-4319076-supplementary/Supplementary File/Supplementary File-Initial Submission/Molecular Docking/Document preparation-ESR1/P2-ES.png]

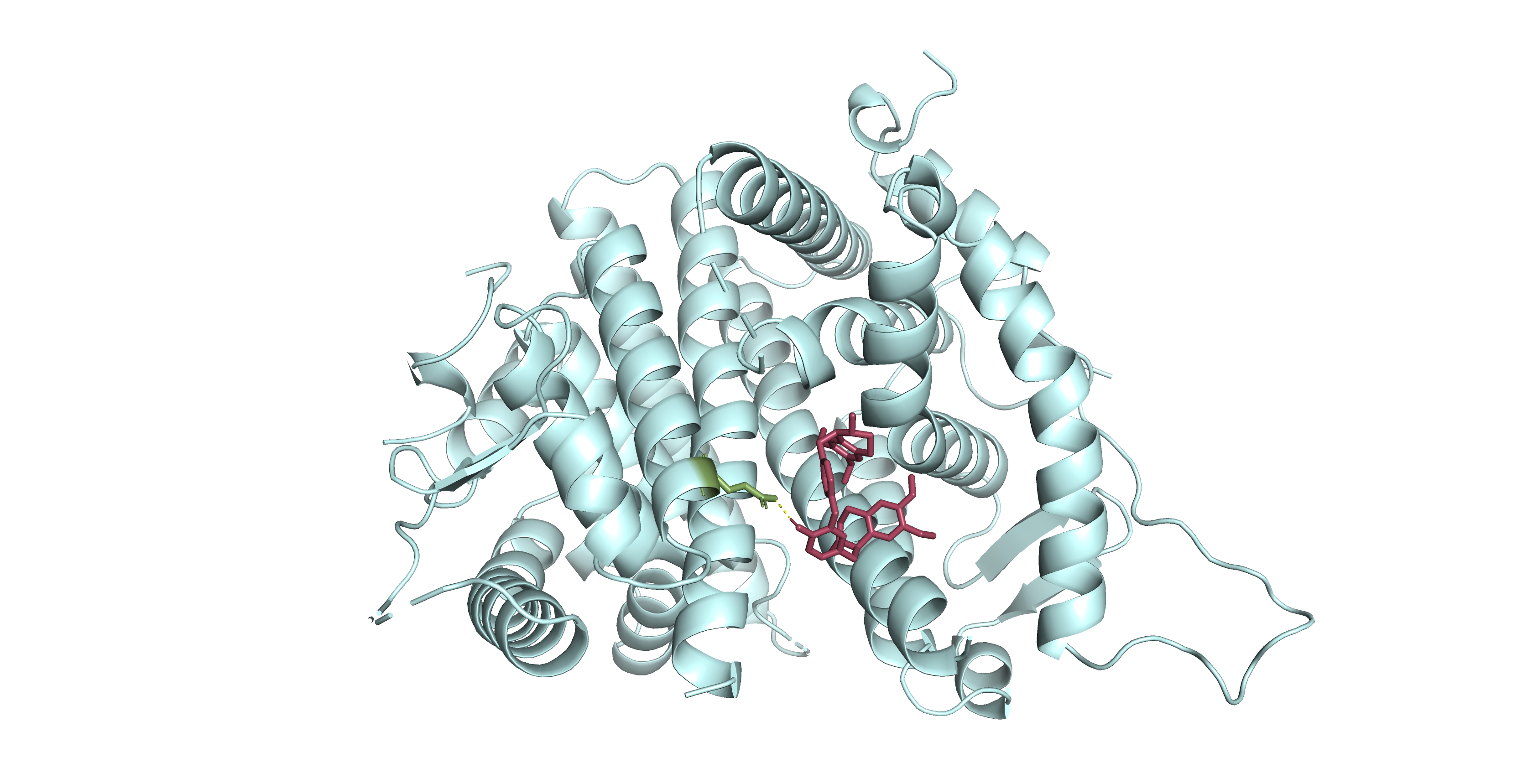

Supplement: Supplementary file 1 [file cimb-48-00550-s001.zip › cimb-4319076-supplementary/Supplementary File/Supplementary File-Initial Submission/Molecular Docking/Document preparation-ESR1/PPPA-ES.png]

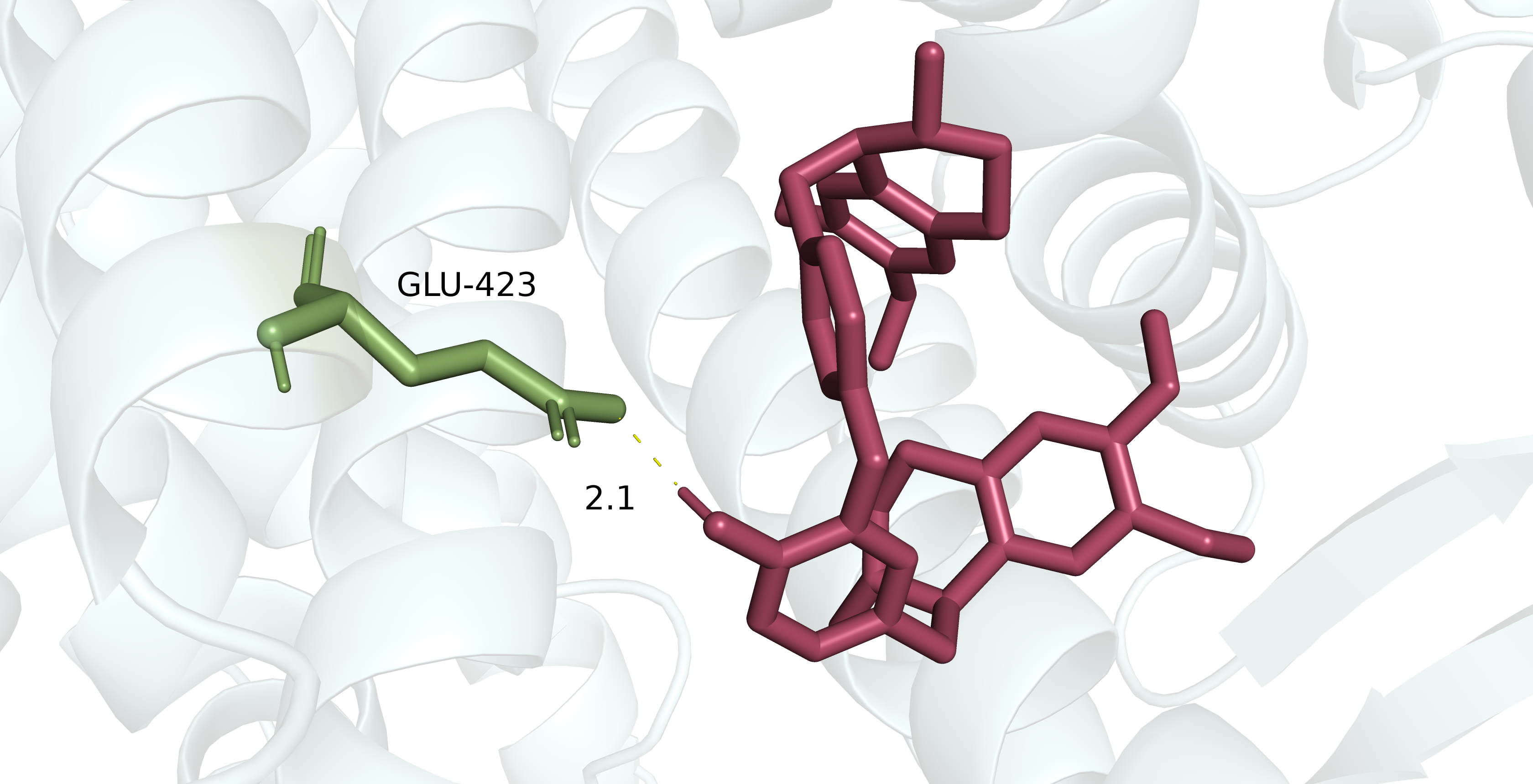

Supplement: Supplementary file 1 [file cimb-48-00550-s001.zip › cimb-4319076-supplementary/Supplementary File/Supplementary File-Initial Submission/Molecular Docking/Document preparation-ESR1/PPPB-ES.png]

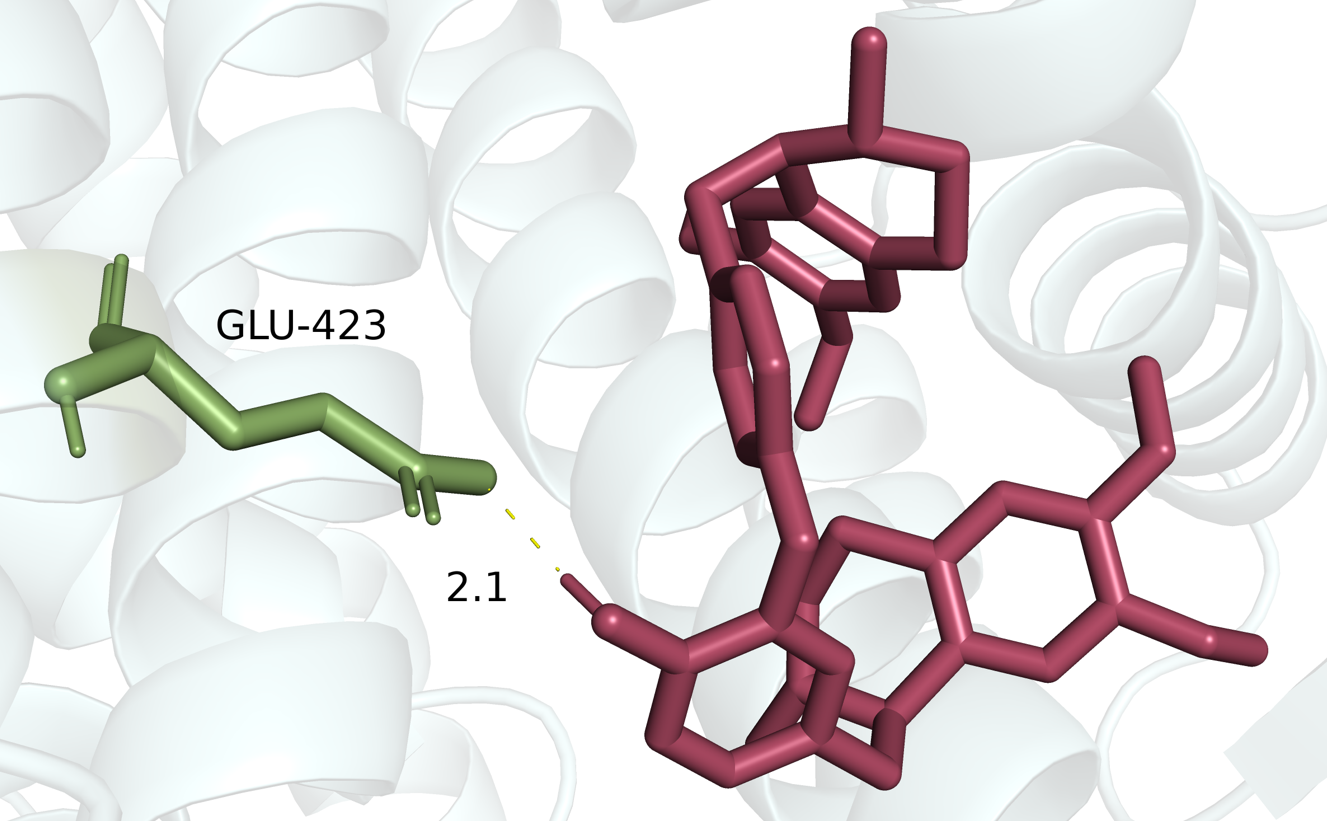

Supplement: Supplementary file 1 [file cimb-48-00550-s001.zip › cimb-4319076-supplementary/Supplementary File/Supplementary File-Initial Submission/Molecular Docking/Document preparation-ESR1/PPPC-ES.png]

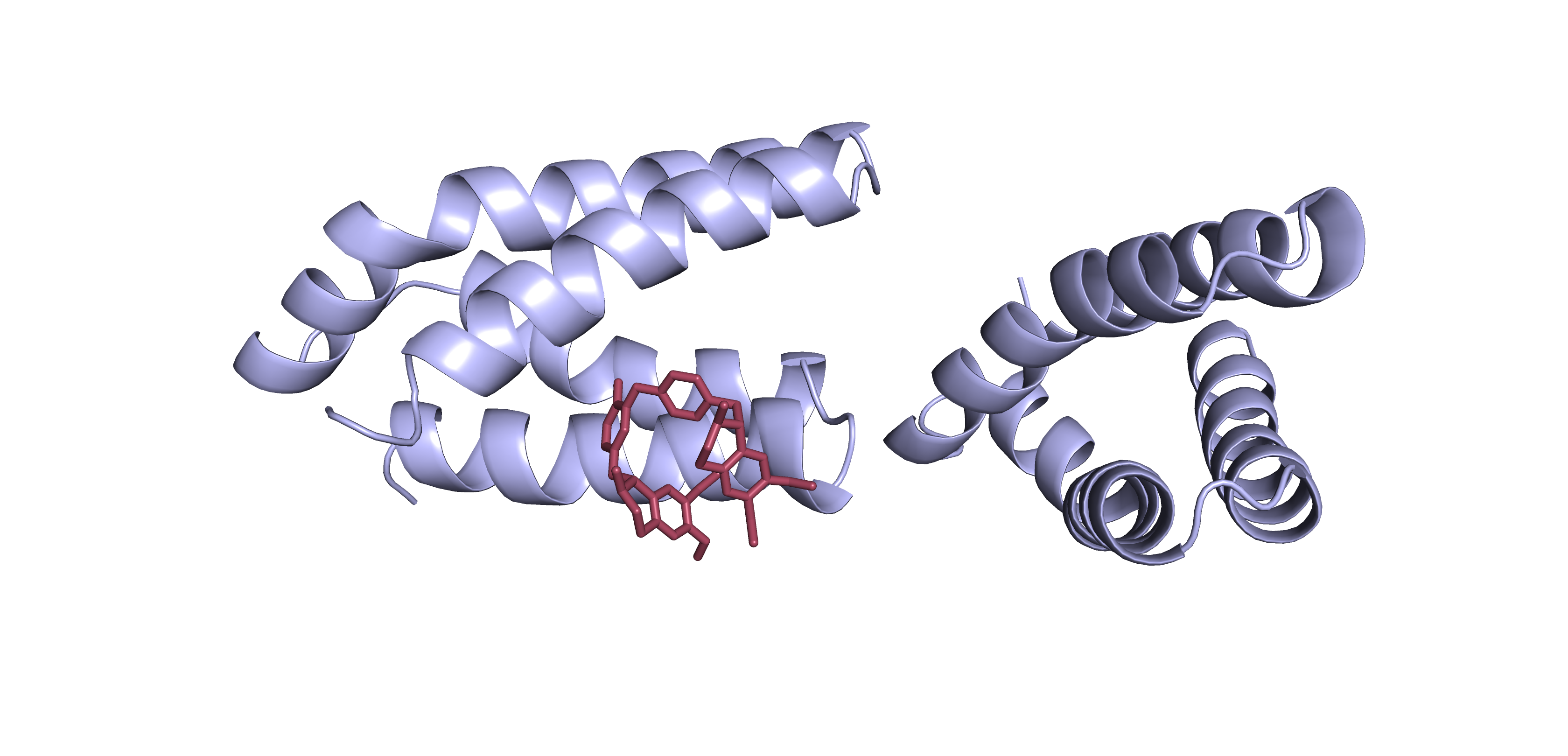

Supplement: Supplementary file 1 [file cimb-48-00550-s001.zip › cimb-4319076-supplementary/Supplementary File/Supplementary File-Initial Submission/Molecular Docking/Document preparation-MTOR/P0.png]

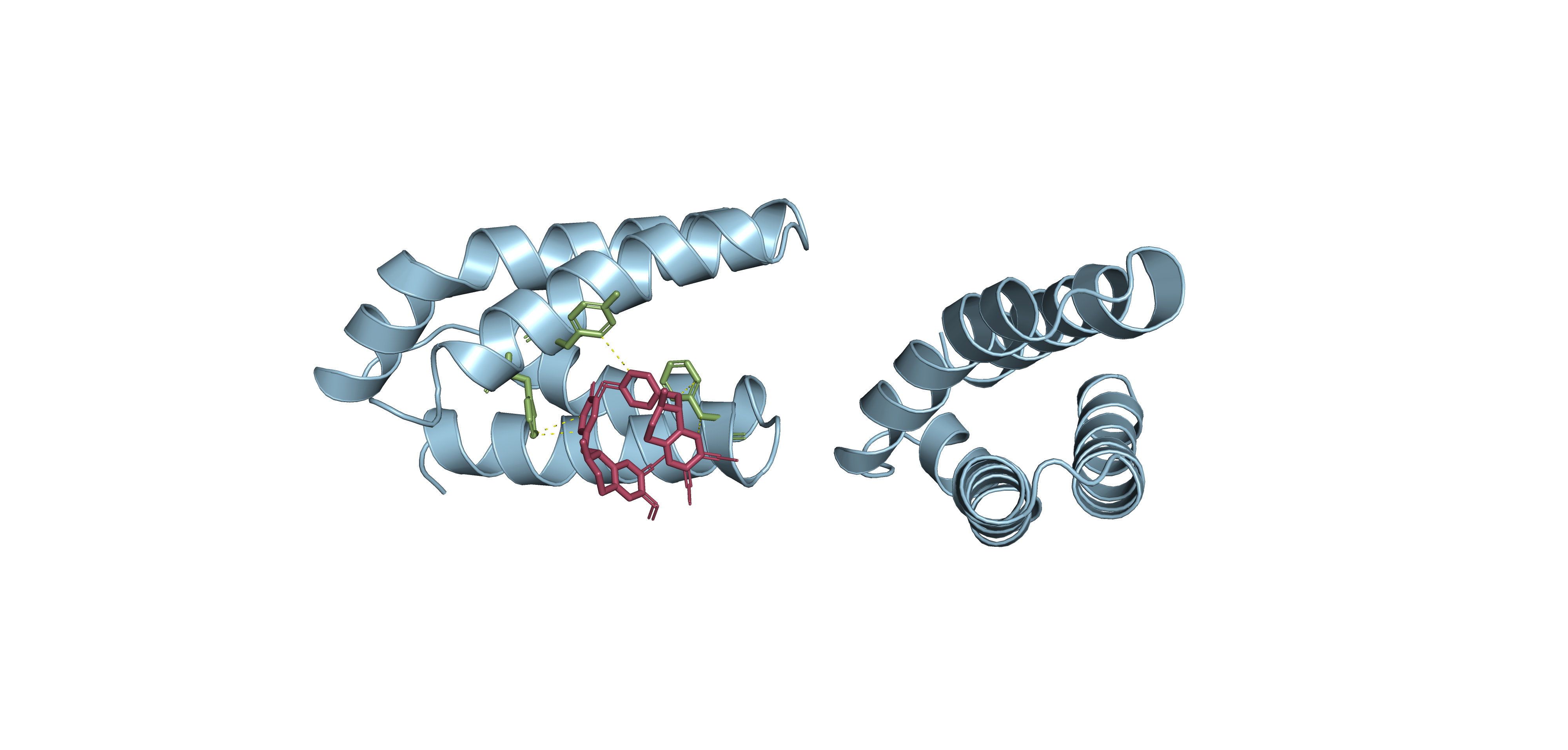

Supplement: Supplementary file 1 [file cimb-48-00550-s001.zip › cimb-4319076-supplementary/Supplementary File/Supplementary File-Initial Submission/Molecular Docking/Document preparation-MTOR/P1.png]

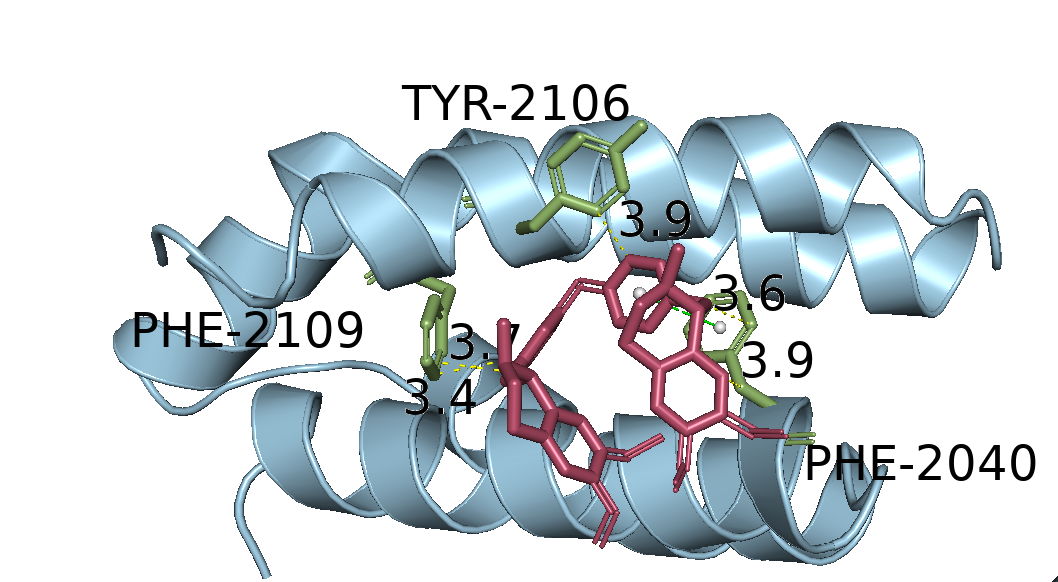

Supplement: Supplementary file 1 [file cimb-48-00550-s001.zip › cimb-4319076-supplementary/Supplementary File/Supplementary File-Initial Submission/Molecular Docking/Document preparation-MTOR/P2.png]

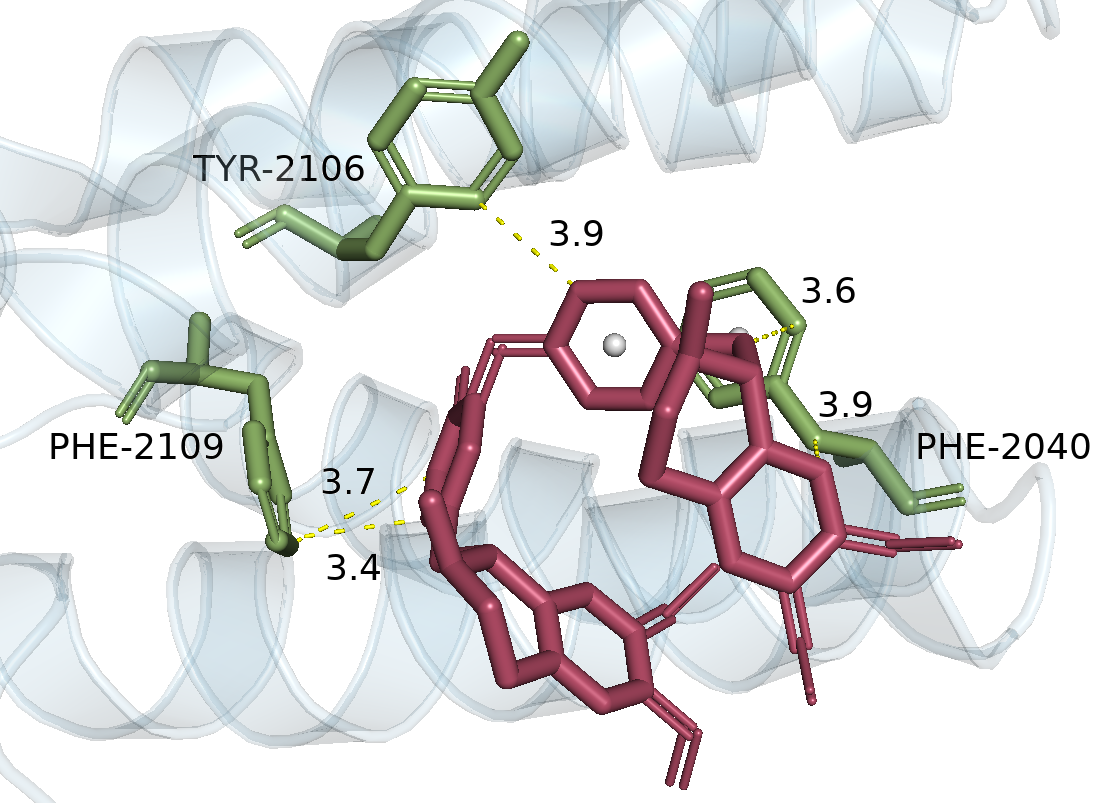

Supplement: Supplementary file 1 [file cimb-48-00550-s001.zip › cimb-4319076-supplementary/Supplementary File/Supplementary File-Initial Submission/Molecular Docking/Document preparation-MTOR/P3.png]

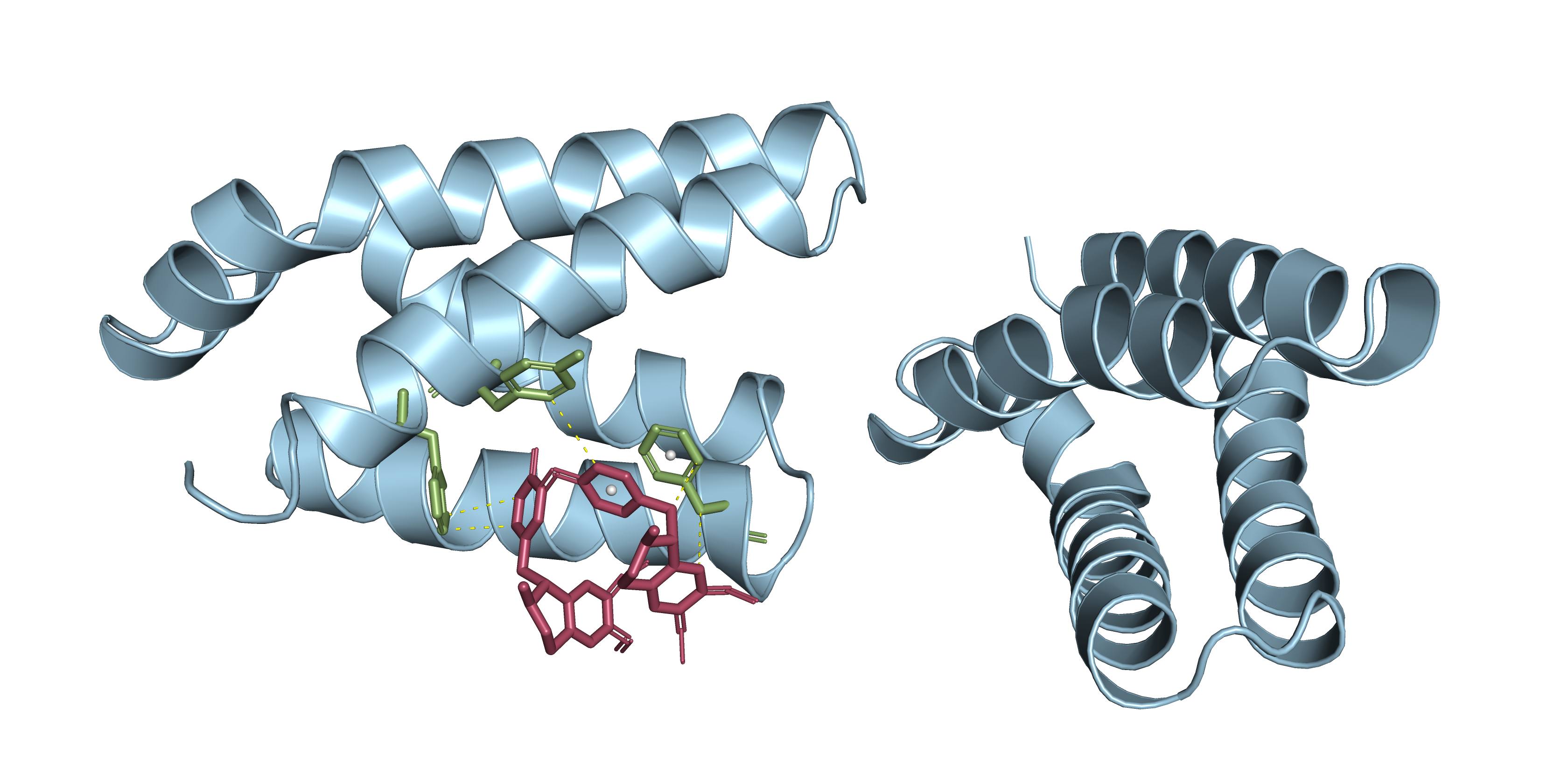

Supplement: Supplementary file 1 [file cimb-48-00550-s001.zip › cimb-4319076-supplementary/Supplementary File/Supplementary File-Initial Submission/Molecular Docking/Document preparation-MTOR/PPPA-MT.png]

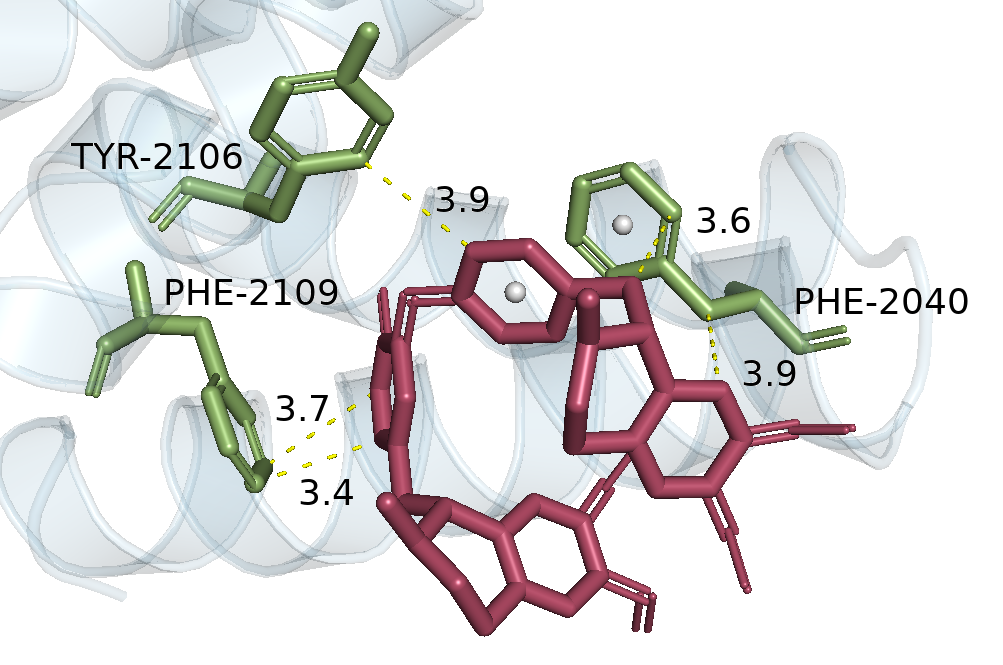

Supplement: Supplementary file 1 [file cimb-48-00550-s001.zip › cimb-4319076-supplementary/Supplementary File/Supplementary File-Initial Submission/Molecular Docking/Document preparation-MTOR/PPPB-MT.png]

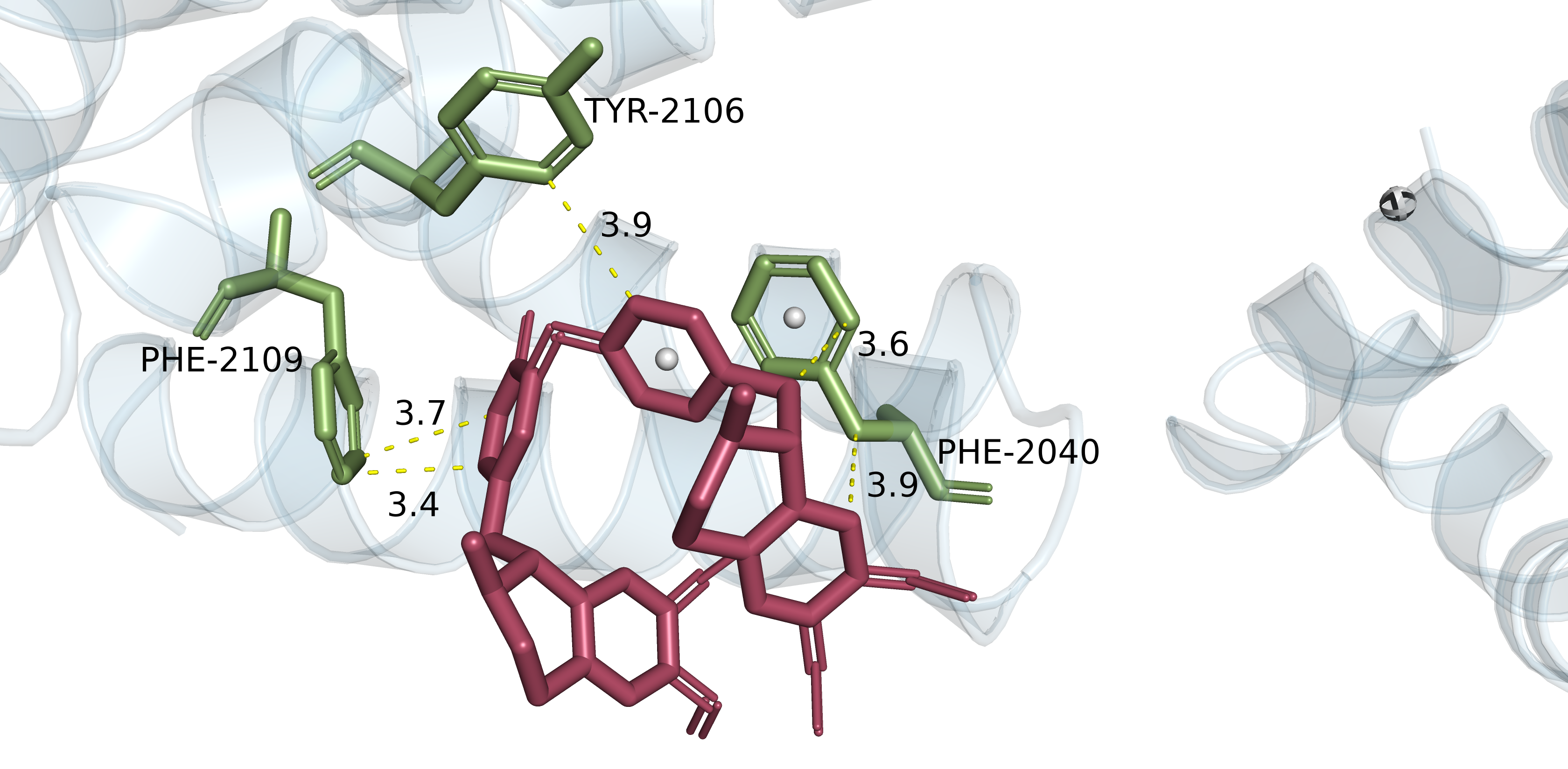

Supplement: Supplementary file 1 [file cimb-48-00550-s001.zip › cimb-4319076-supplementary/Supplementary File/Supplementary File-Initial Submission/Molecular Docking/Document preparation-MTOR/PPPC-MT.png]

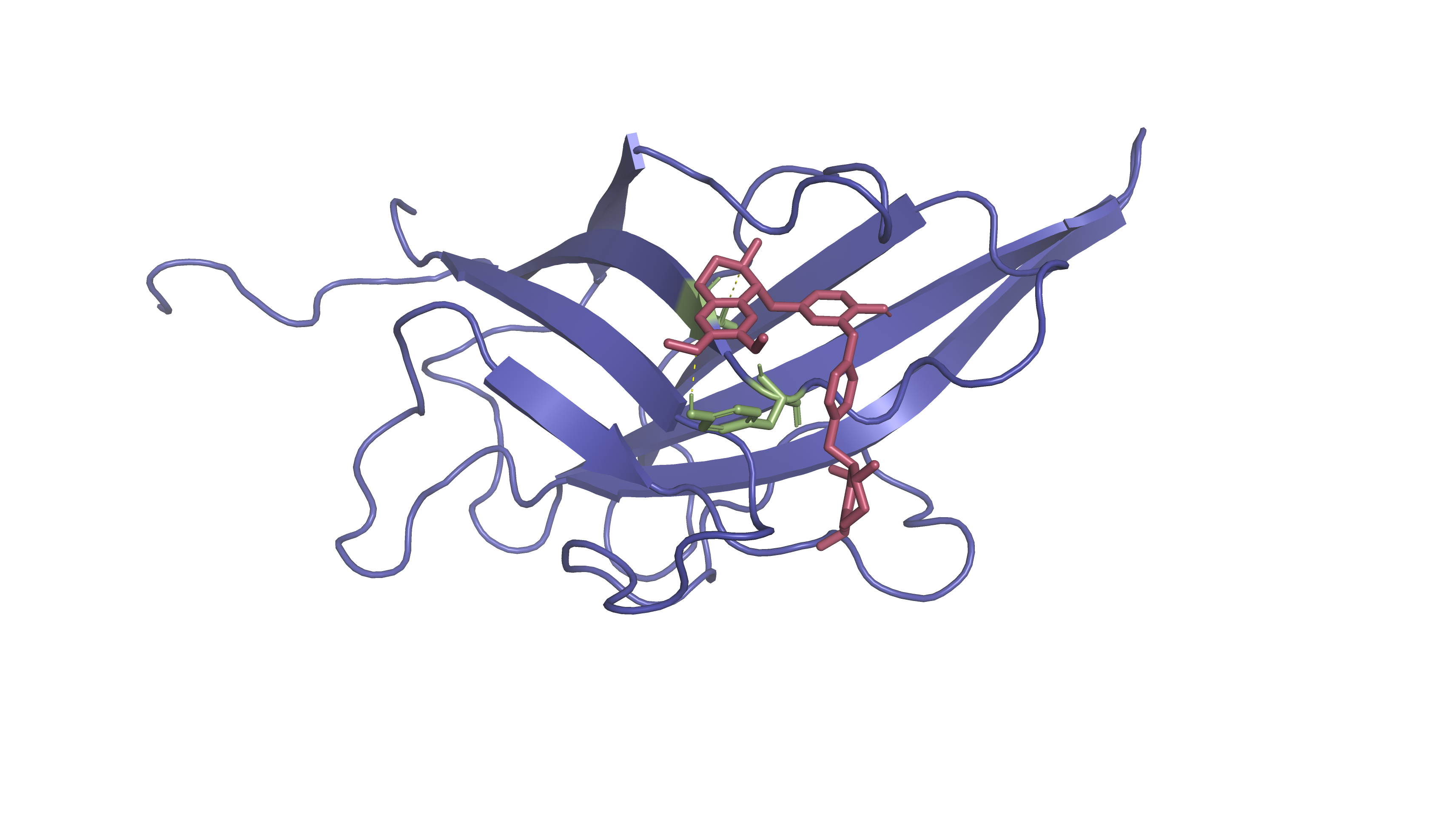

Supplement: Supplementary file 1 [file cimb-48-00550-s001.zip › cimb-4319076-supplementary/Supplementary File/Supplementary File-Initial Submission/Molecular Docking/Document preparation-PIK3CA/P1-PI.png]

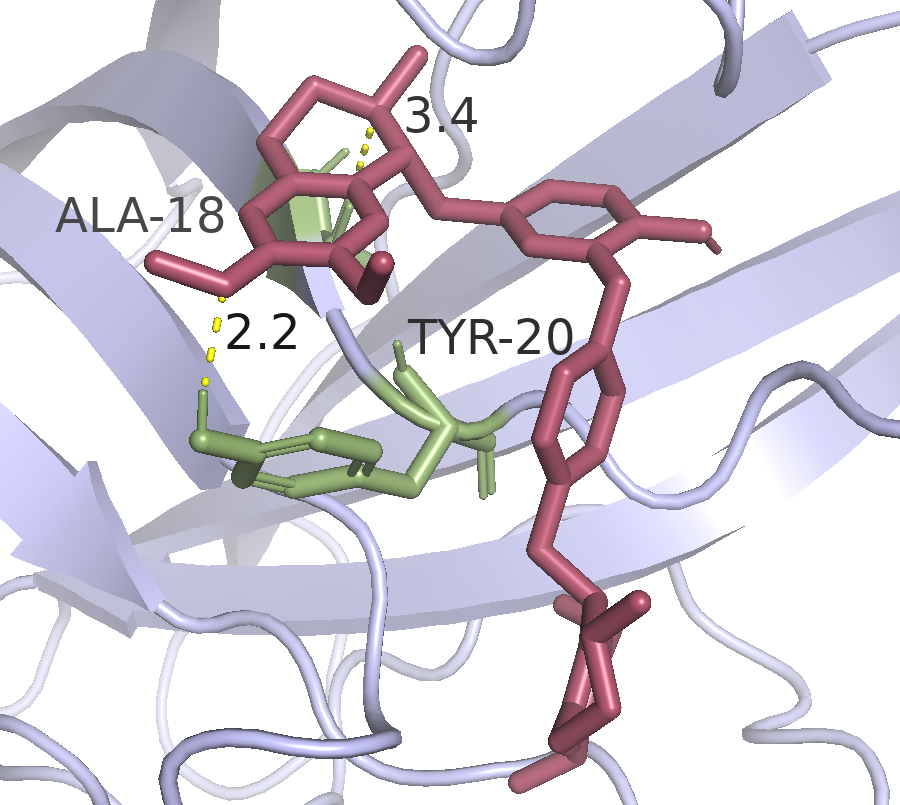

Supplement: Supplementary file 1 [file cimb-48-00550-s001.zip › cimb-4319076-supplementary/Supplementary File/Supplementary File-Initial Submission/Molecular Docking/Document preparation-PIK3CA/P2-PI.png]

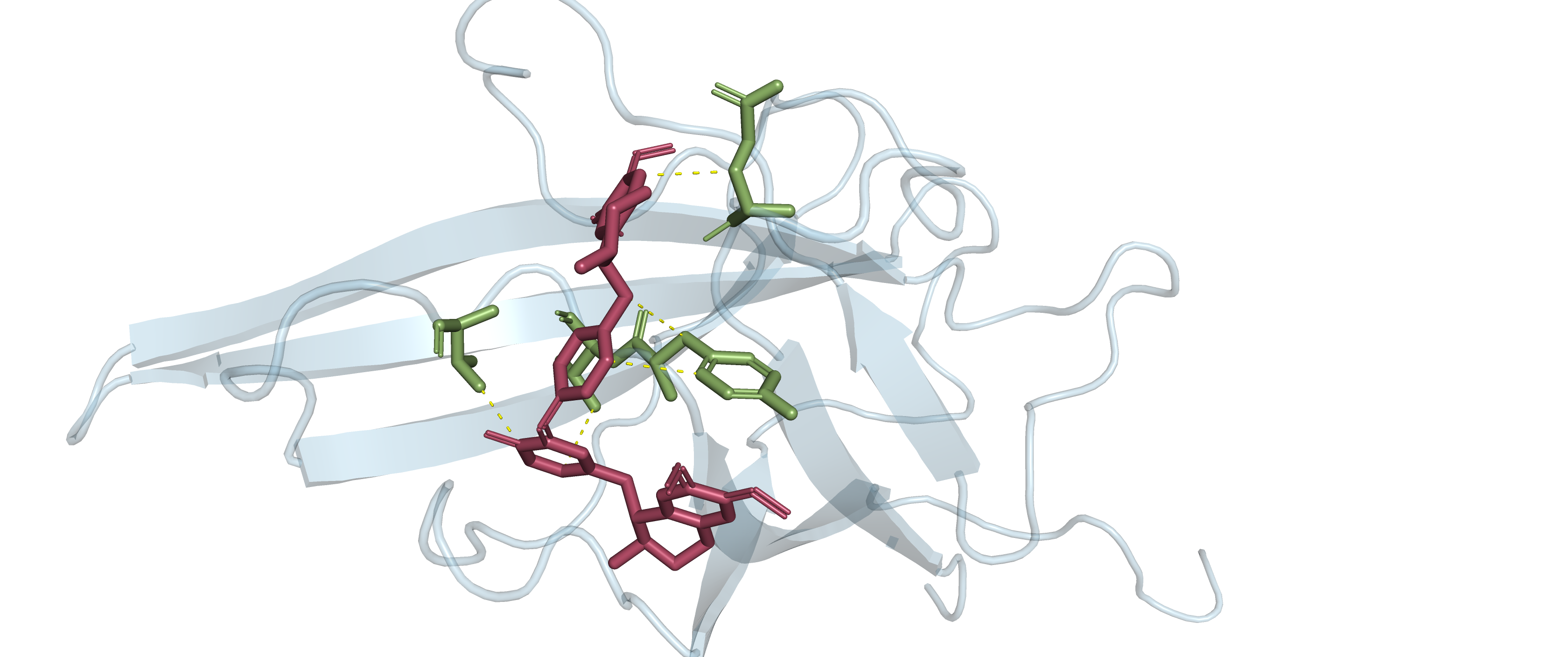

Supplement: Supplementary file 1 [file cimb-48-00550-s001.zip › cimb-4319076-supplementary/Supplementary File/Supplementary File-Initial Submission/Molecular Docking/Document preparation-PIK3CA/PA-PI.png]

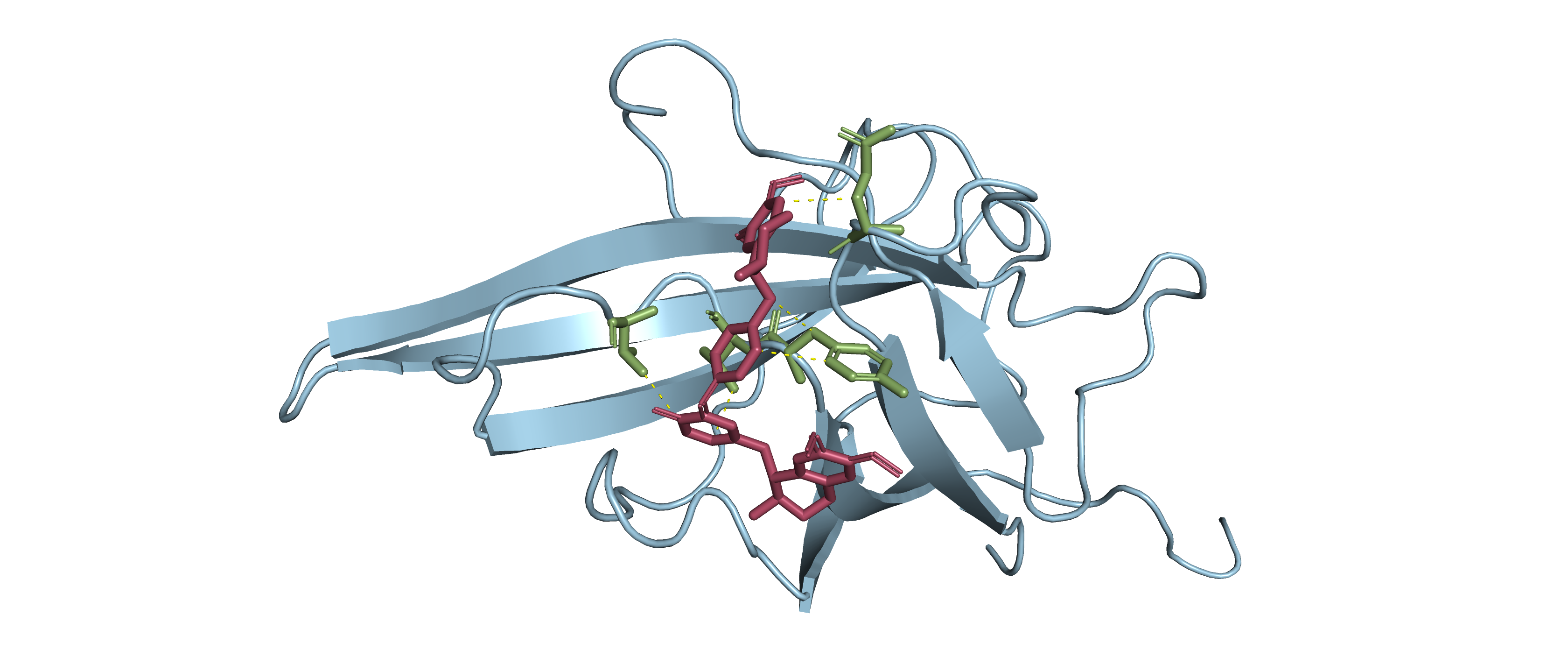

Supplement: Supplementary file 1 [file cimb-48-00550-s001.zip › cimb-4319076-supplementary/Supplementary File/Supplementary File-Initial Submission/Molecular Docking/Document preparation-PIK3CA/PPPA-PI.png]

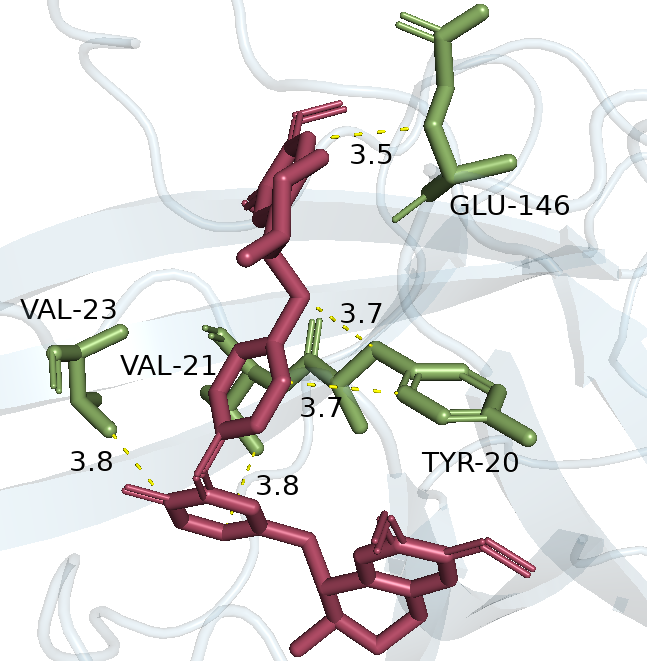

Supplement: Supplementary file 1 [file cimb-48-00550-s001.zip › cimb-4319076-supplementary/Supplementary File/Supplementary File-Initial Submission/Molecular Docking/Document preparation-PIK3CA/PPPB-PI.png]

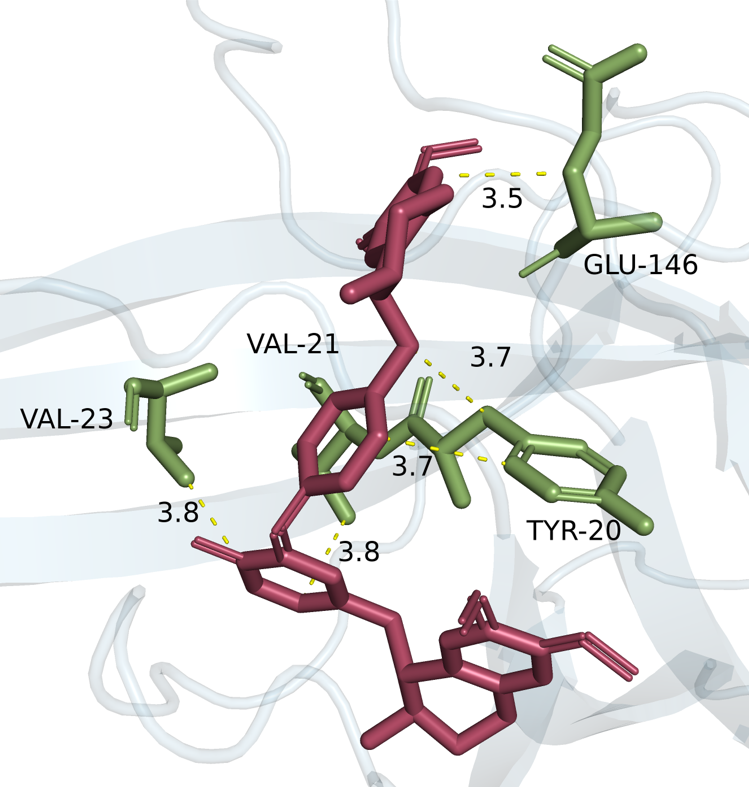

Supplement: Supplementary file 1 [file cimb-48-00550-s001.zip › cimb-4319076-supplementary/Supplementary File/Supplementary File-Initial Submission/Molecular Docking/Document preparation-PIK3CA/PPPC-PI.png]

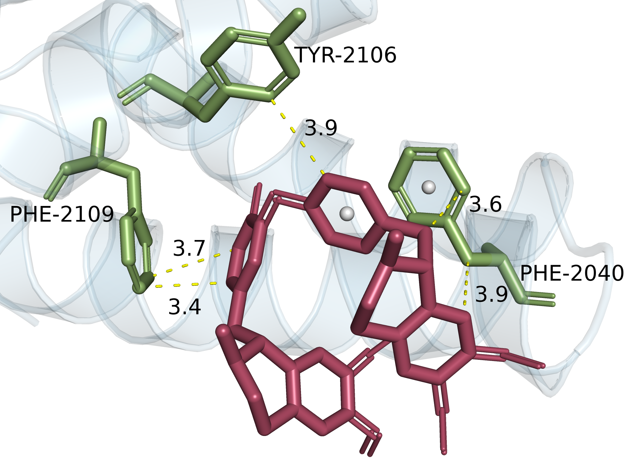

Supplement: Supplementary file 1 [file cimb-48-00550-s001.zip › cimb-4319076-supplementary/Supplementary File/Supplementary File-Initial Submission/Molecular Docking/Picture/PB-MT.png]

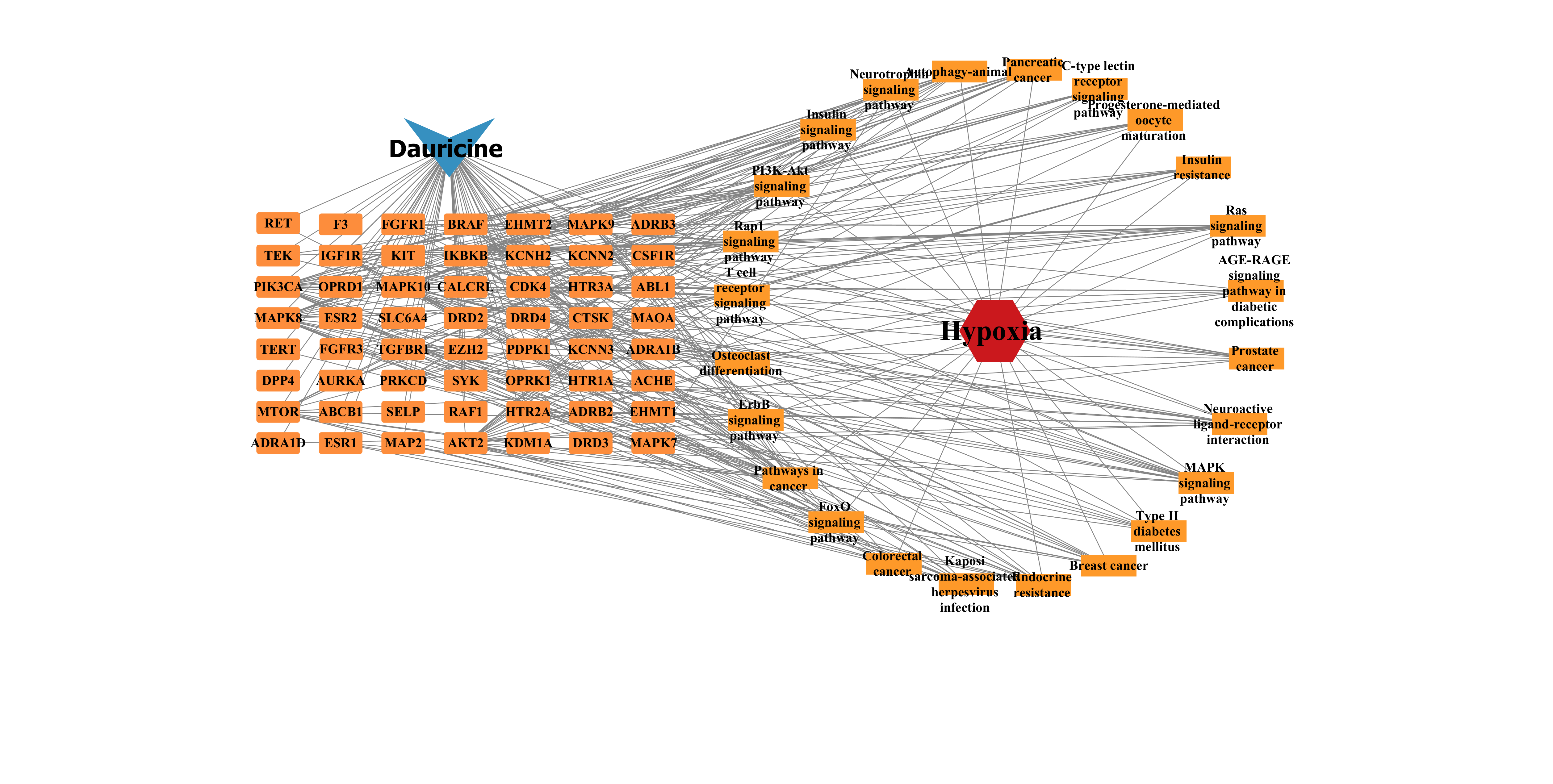

Supplement: Supplementary file 1 [file cimb-48-00550-s001.zip › cimb-4319076-supplementary/Supplementary File/Supplementary File-Initial Submission/Network/Final.png]

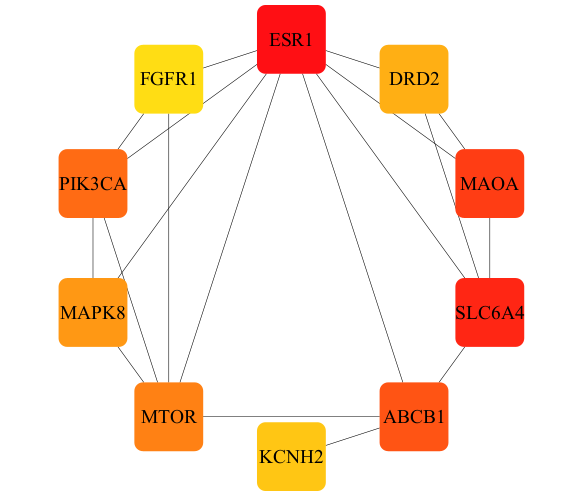

Supplement: Supplementary file 1 [file cimb-48-00550-s001.zip › cimb-4319076-supplementary/Supplementary File/Supplementary File-Initial Submission/PPI/cytoHubba/six algorithms/Degree.png]

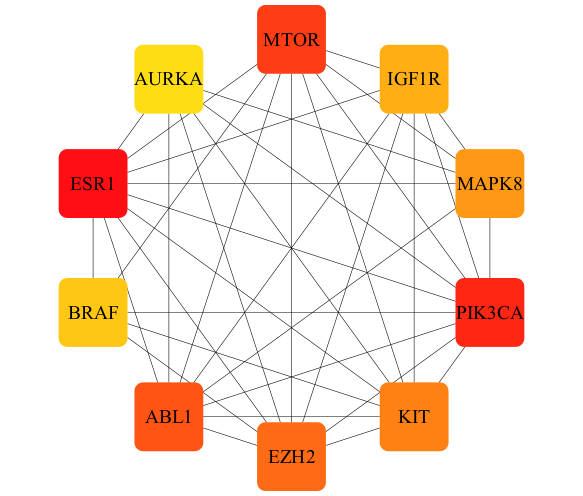

Supplement: Supplementary file 1 [file cimb-48-00550-s001.zip › cimb-4319076-supplementary/Supplementary File/Supplementary File-Initial Submission/PPI/cytoHubba/six algorithms/EPC.png]

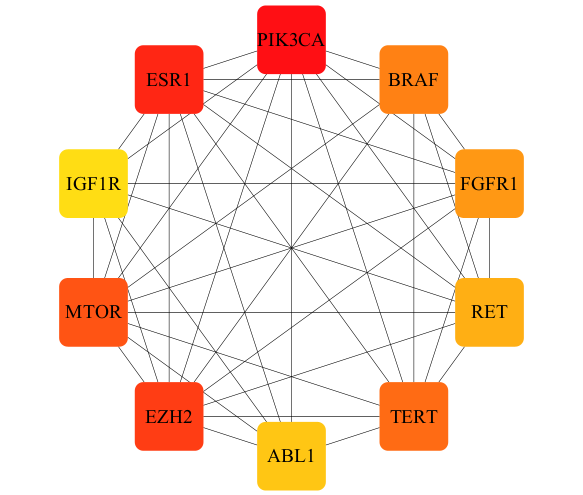

Supplement: Supplementary file 1 [file cimb-48-00550-s001.zip › cimb-4319076-supplementary/Supplementary File/Supplementary File-Initial Submission/PPI/cytoHubba/six algorithms/MCC.png]

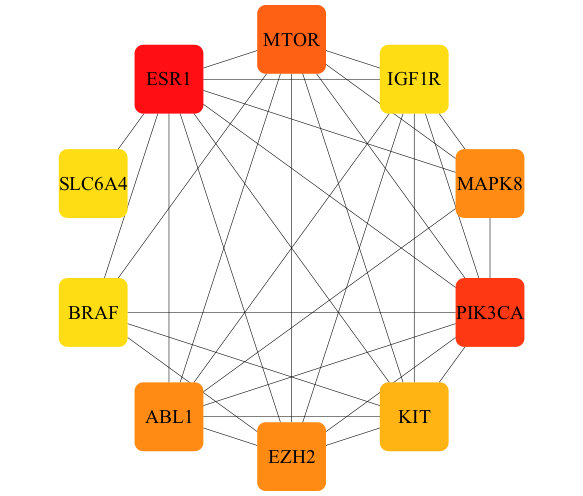

Supplement: Supplementary file 1 [file cimb-48-00550-s001.zip › cimb-4319076-supplementary/Supplementary File/Supplementary File-Initial Submission/PPI/cytoHubba/six algorithms/MNC.png]

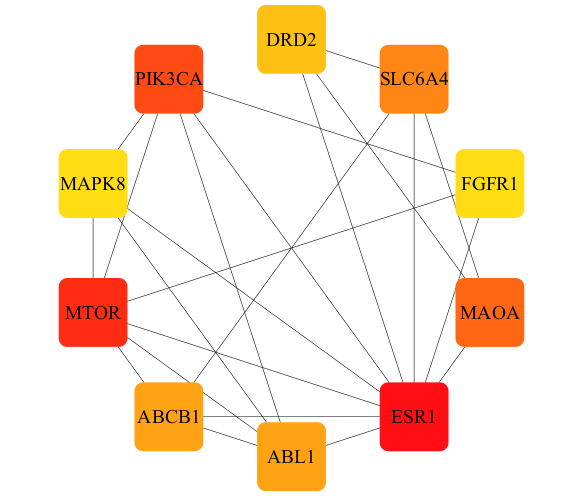

Supplement: Supplementary file 1 [file cimb-48-00550-s001.zip › cimb-4319076-supplementary/Supplementary File/Supplementary File-Initial Submission/PPI/cytoHubba/six algorithms/Radiality.png]

## Slide 1
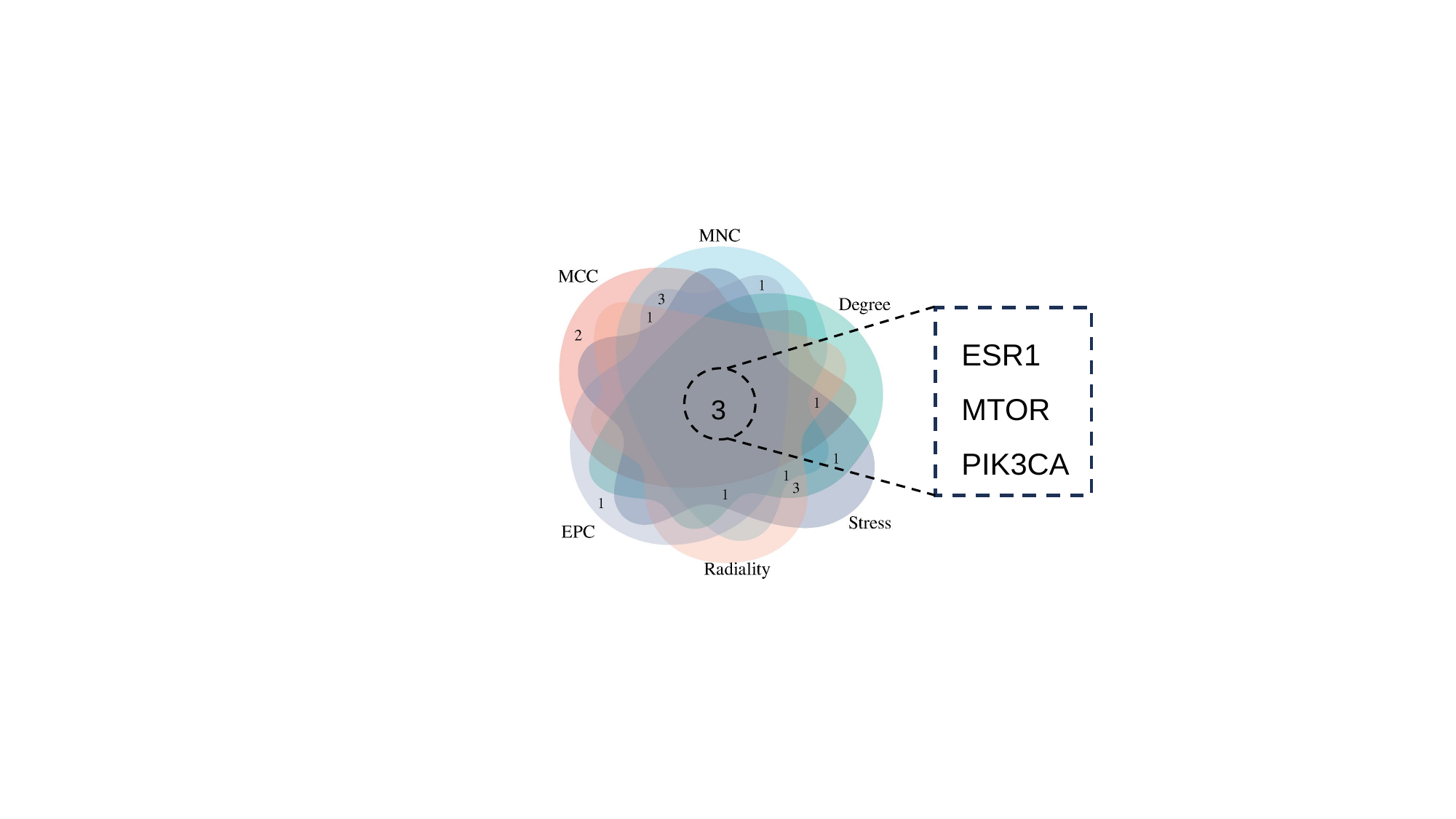

ESR1
MTOR
PIK3CA
3

Supplement: Supplementary file 1 [file cimb-48-00550-s001.zip › cimb-4319076-supplementary/Supplementary File/Supplementary File-Initial Submission/PPI/cytoHubba/six algorithms-core targets.pptx]

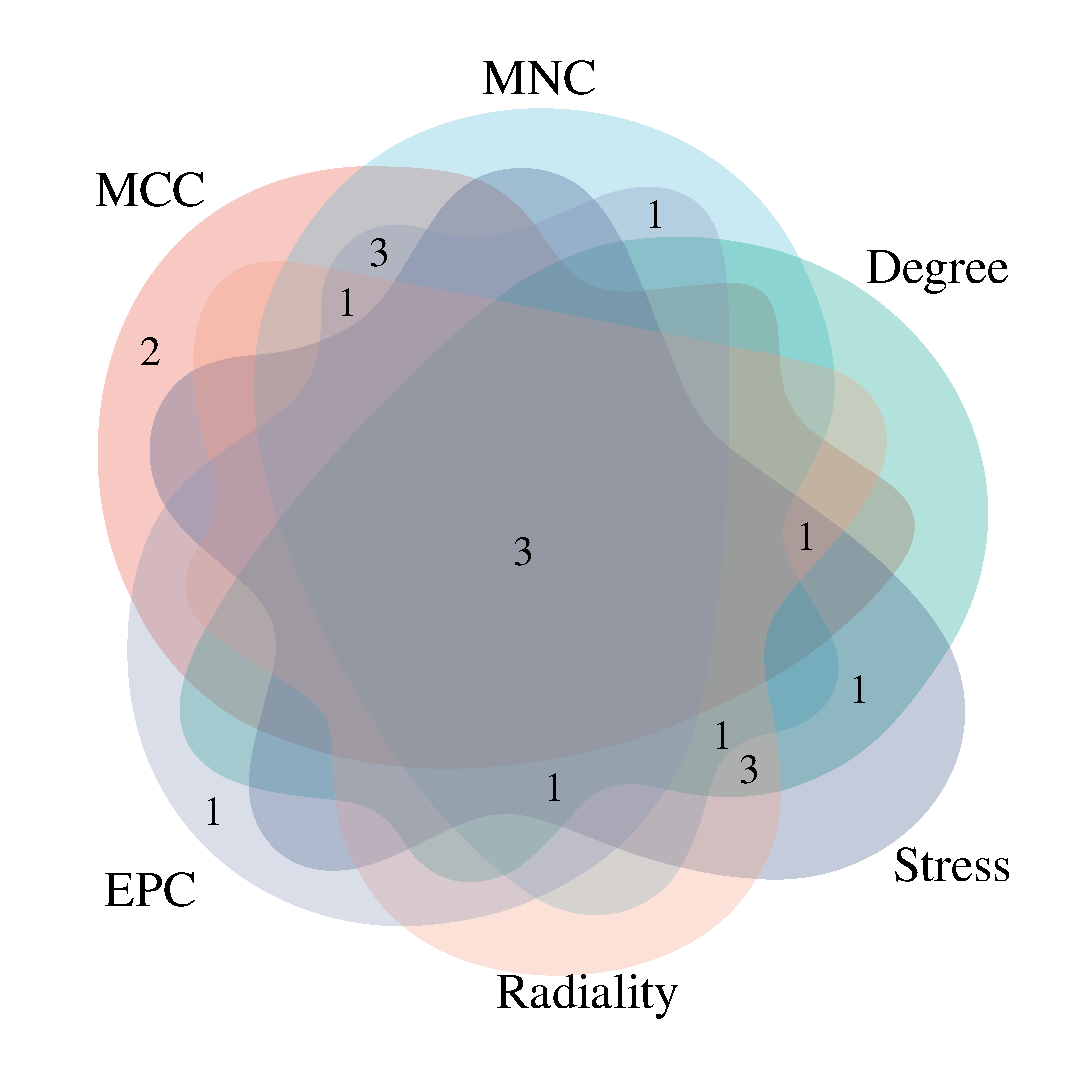

Supplement: Supplementary file 1 [file cimb-48-00550-s001.zip › cimb-4319076-supplementary/Supplementary File/Supplementary File-Initial Submission/PPI/cytoHubba/VENN 300dpi.png]

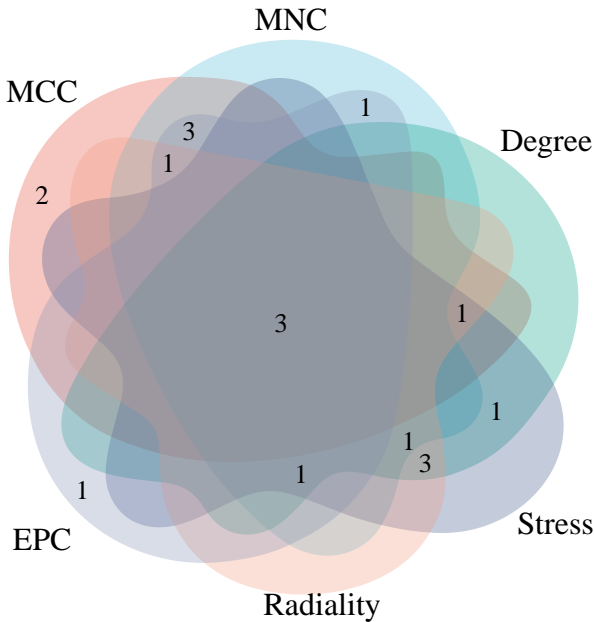

Supplement: Supplementary file 1 [file cimb-48-00550-s001.zip › cimb-4319076-supplementary/Supplementary File/Supplementary File-Initial Submission/PPI/cytoHubba/VENN PDF.pdf]

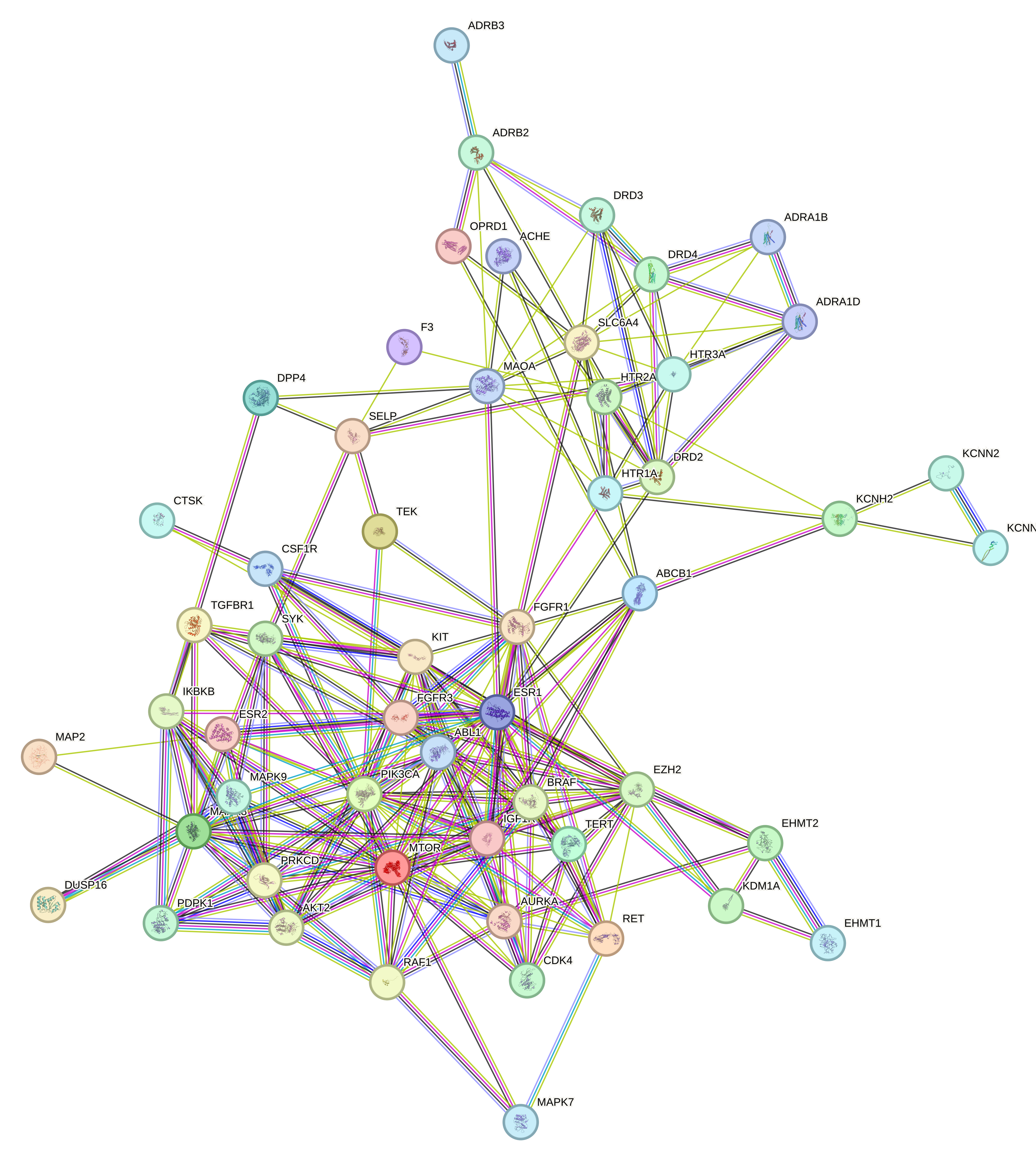

Supplement: Supplementary file 1 [file cimb-48-00550-s001.zip › cimb-4319076-supplementary/Supplementary File/Supplementary File-Initial Submission/PPI/string_hires_image 0.4.png]
